# METTL14 contributes to acute lung injury by stabilizing NLRP3 expression in an IGF2BP2-dependent manner

**DOI:** 10.1038/s41419-023-06407-6

**Published:** 2024-01-13

**Authors:** Fei Cao, Guojun Chen, Yixin Xu, Xintong Wang, Xiaole Tang, Wenyu Zhang, Xiong Song, Xiaohua Yang, Weian Zeng, Jingdun Xie

**Affiliations:** Department of Anesthesiology, State Key Laboratory of Oncology in South China, Guangdong Provincial Clinical Research Center for Cancer, Sun Yat-sen University Cancer Center, Guangzhou, 510060 P. R. China

**Keywords:** Inflammasome, Epigenetics

## Abstract

Acute lung injury (ALI) as well as its more severe form, acute respiratory distress syndrome (ARDS), frequently leads to an uncontrolled inflammatory response. N^6^-methyladenosine (m^6^A) modification was associated with the progression of several inflammatory diseases. However, the role of methyltransferase-like 14 (METTL14)-mediated m^6^A methylation in ALI/ARDS remains unclear. Here, we reported an increase in overall expression levels of m^6^A and METTL14 in circulating monocyte-derived macrophages recruited to the lung following ALI, which is correlated with the severity of lung injury. We further demonstrated the critical function of METTL14 in activating NOD-like receptor pyrin domain-containing protein 3 (NLRP3) inflammasome in vitro and in mouse models of ALI/ARDS, and validated NLRP3 as the downstream target of METTL14 by the m^6^A RNA immunoprecipitation (MeRIP) and RIP assays. Mechanistically, METTL14-methylated NLRP3 transcripts were subsequently recognized by insulin-like growth factor 2 mRNA-binding protein 2 (IGF2BP2), an m^6^A reader, which stabilized NLRP3 mRNA. Furthermore, we observed that IGF2BP2 knockdown diminished LPS-induced ALI in mice by downregulating NLRP3 expression. In summation, our study revealed that the molecular mechanism underlying the pathogenesis of ALI/ARDS involves METTL14-mediated activation of NLRP3 inflammasome in an IGF2BP2 dependent manner, thereby demonstrating the potential of METTL14 and IGF2BP2 as promising biomarkers and therapeutic targets for ALI/ARDS treatment.

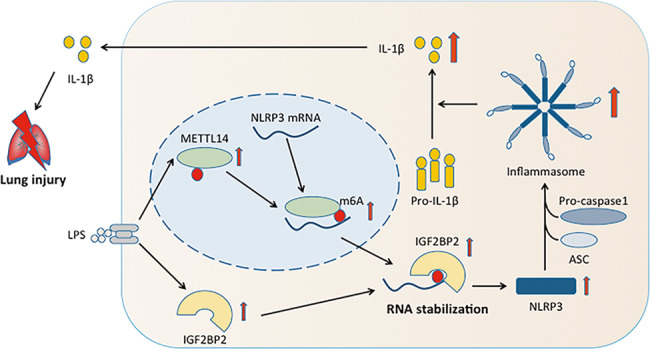

## Introduction

Acute lung injury (ALI) and its more severe form, acute respiratory distress syndrome (ARDS) are common, life-threatening critical illnesses that lead to significant morbidity and mortality [1]. Despite prominent breakthroughs in the pathophysiology of ALI/ARDS, the hospital mortality rate of these disorders remains high (46.1%), and effective pharmacological treatments are still lacking [2]. ALI/ARDS is characterized by dysregulated lung parenchymal inflammation, leading to diffuse alveolar damage and edema, ultimately contributing to acute hypoxemic respiratory failure [3]. Uncontrolled local or systemic inflammation is believed to be the predominant cause of ALI/ARDS [4]. Activated macrophages, especially recruited circulating monocyte-derived macrophages further release pro-inflammatory cytokines, which give rise to an inflammation cascade [5].

The NOD-like receptor pyrin domain-containing protein 3 (NLRP3) inflammasome is excessively activated in macrophages during ALI/ARDS progression [6]. The NLRP3 inflammasome, which consists of a sensor (NLRP3), an adaptor apoptosis-associated speck-like protein containing a caspase-recruitment domain (ASC), and an effector caspase (caspase-1), is involved in the production of pro-inflammatory cytokines, interleukin (IL)-1β and IL-18 [7]. NLRP3 inflammasome activation reportedly involves two steps: priming and activation [8]. The priming step of NLRP3 inflammasome activation is regulated via transcriptional and post-translational mechanisms [9]. NF-κB signaling induces the transcriptional activation of NLRP3 priming by upregulating the gene expression of NLRP3 inflammasome components [10, 11]. Post-translational modifications (PTMs) of NLRP3, such as ubiquitination, phosphorylation, and SUMOylation, may stabilize NLRP3 in an auto-suppressed inactive state [12]. The activation step is induced by various pathogen-associated molecular patterns (PAMPs) and damage-associated molecular patterns (DAMPs), including extracellular ATP, pore-forming toxins, RNA viruses, and particulate matter [13]. However, post-transcriptional regulation of NLRP3 inflammasome activation in ALI/ARDS remains unclear.

N^6^-methyladenosine (m^6^A) modification, the most abundant modification of messenger RNA (mRNA), may reversibly regulate target genes at the post-transcriptional level, thereby affecting almost all crucial biological processes [14]. This dynamic and reversible process is primarily regulated by the m^6^A methyltransferase complex, which contains methyltransferase-like 3 (METTL3), methyltransferase-like 14 (METTL14), Wilms tumor suppressor-1-associated protein (WTAP), and demethylases, including fat mass and obesity-related protein (FTO) and ALKB homolog 5 protein (ALKBH5) [15]. Meanwhile, RNA-binding proteins that identify and bind to m^6^A sites, such as the YT521-B homology domain (YTHD) family, and the insulin-like growth factor 2 mRNA-binding protein (IGF2BP) family, serve as m^6^A readers and direct the fate of target RNAs by influencing alternative pre-mRNA splicing, RNA stability, and translation efficiency [16].

m^6^A facilitates the progression of several inflammatory diseases, such as non-alcoholic fatty liver disease, autoimmune diseases, and infections [17–20]. Evidently, global m^6^A levels are significantly increased in alveolar epithelial cells, mediated by the upregulation of METTL3, which is closely associated with ALI [21]. Nonetheless, the effects of METTL14-regulated m^6^A methylation in ALI/ARDS remain unclear and the precise molecular targets of METTL14 in ALI/ARDS remain to be elucidated. Therefore, we sought to determine the functional role of METTL14 and its target in ALI/ARDS.

Herein, we first elucidated that RNA m^6^A modification in macrophages is involved in the progression of ALI/ARDS, and then verified the role of METTL14 in this process. Further mechanistic studies revealed that NLRP3 is the methylated target of METTL14 and that IGF2BP2 stabilizes NLRP3 mRNA during NLRP3 inflammasome activation in ALI/ARDS. These results indicated that METTL4 together with IGF2BP2 may be promising therapeutic targets in ALI/ARDS.

## Results

### Global m^6^A levels and METTL14 expression are increased in ALI mice

**Fig. 1:**
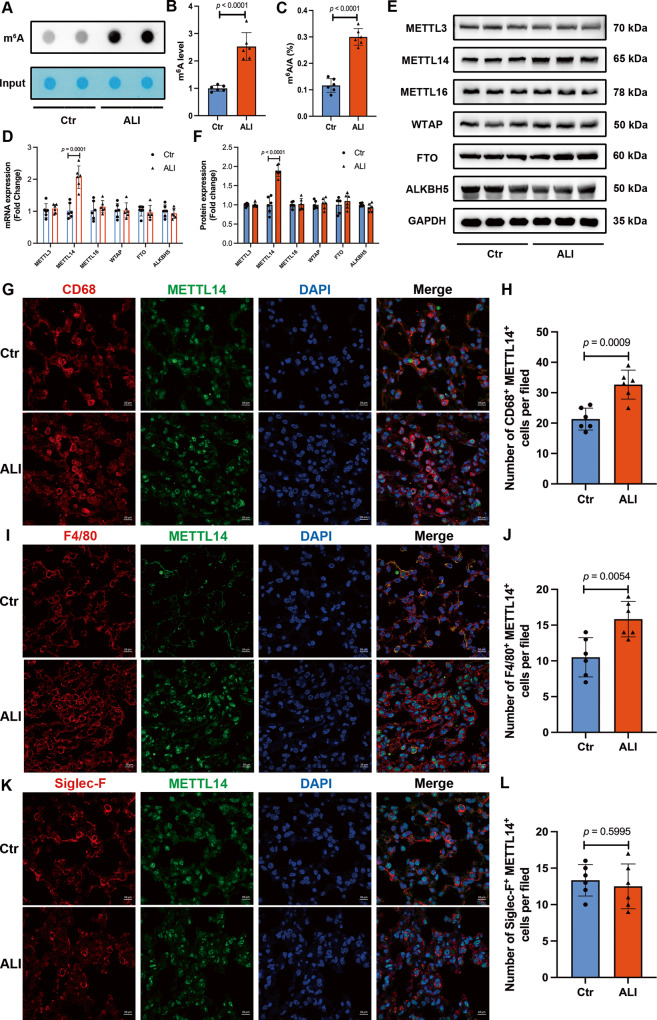
Global m^6^A levels and METTL14 expression are increased in ALI mice. Mice were injected intraperitoneally with LPS (15 mg/kg) or an equal volume of saline. **A**, **B** m^6^A dot-blot assays of lung tissues from Ctr and ALI mice. **C** A colorimetric assay measured m^6^A mRNA methylation in lung tissues of each group. **D** RT-qPCR analysis and (**E**, **F**) Western blot analysis of m^6^A regulators (METTL3, METTL14, METTL16, WTAP, FTO, and ALKBH5) in lung tissues of Ctr and ALI mice. **G**–**L** The immunofluorescence co-staining analysis of METTL14 (green) and CD68 (a macrophage marker, red) (**G**, **H**), F4/80 (a macrophage marker, red) (**I**, **J**), or Siglec-F (a resident alveolar macrophage marker, red) (**K**, **L**) in mouse lungs. The graphs depict mean ± SD, *n* = 6 mice per group.

To confirm the role of m^6^A modification in ALI/ARDS, we evaluated the global m^6^A levels in the lung tissues of control and ALI mice. Both dot blot assay and colorimetric quantification showed that global m^6^A levels in the total RNA were significantly increased in ALI lungs compared to the control group (Fig. 1A–C). We then detected the expression of m^6^A methyltransferases (METTL3, METTL14, METTL16, and WTAP) and demethylases (FTO and ALKBH5) in lung tissues. The expression of METTL14 mRNA and METTL14 protein was markedly upregulated in ALI mice, whereas no significant differences were found in the expression of other regulators (Fig. 1D–F). These results indicated that METTL14-mediated m^6^A methylation may play a functional role in ALI/ARDS. Subsequently, we employed immunofluorescence staining to identify the specific cell types involved in ALI/ARDS that express METTL14. Our findings revealed co-localization of METTL14 not only with CD68-labeled macrophages but also with CK7-labeled pulmonary epithelial cells. Interestingly, compared with sham group, ALI lungs exhibited an increased number of METTL14-expressed CD68^+^ macrophages, while the number of METTL14-expressed CK7^+^ epithelial cells did not show a significant change (Figs. 1G, H and S1A, B). To clarify the origins of elevated METTL14^+^ macrophages, quantitative analysis unveiled that METTL14^+^/ F4/80^+^, rather than METTL14^+^/Siglec-F^+^ (a marker for resident alveolar macrophage) cells increased in ALI mice compared with the corresponding sham mice (Fig. 1I–L). Collectively, these findings indicated that the expression level of METTL14 is elevated in LPS-induced ALI model, particularly in recruited circulating monocyte-derived macrophages within the lung.

### Global m^6^A levels and METTL14 expression are increased in LPS-activated macrophages

**Fig. 2:**
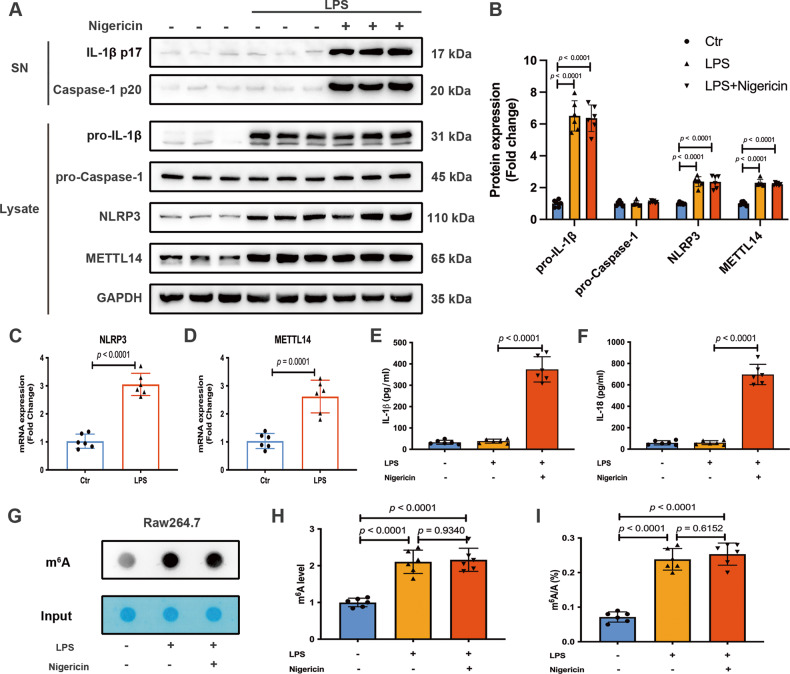
Global m^6^A levels and METTL14 expression are increased in LPS-activated macrophages. **A**, **B** Western blot analysis of IL-1β p17, Caspase-1 p20 in supernatants (SN) and pro-IL-1β, pro-Caspase-1, NLRP3, METTL14 in cell extracts (Lysate) of RAW264.7 cells treated with or without nigericin for 30 min after LPS pretreatment for 6 h, when compared with control. **C**, **D** RT-qPCR analysis of NLRP3 and METTL14 mRNA levels in RAW264.7 cells. **E**, **F** The IL-1β and IL-18 concentrations in supernatants of the indicated RAW264.7 cells. **G**, **H** m^6^A dot-blot assays and (**I**) A colorimetric assay measured m^6^A mRNA methylation in RAW264.7 cells treated with or without nigericin for 30 min after LPS pretreatment for 6 h, when compared with control. All data are represented as mean ± SD, *n* = 6 replicates of each condition.

The NLRP3 inflammasome is a crucial factor in triggering the activation of macrophages during this pathogenesis [22]. We subsequently assessed the expression level of METTL14 within a NLRP3 inflammasome activation model in RAW264.7 macrophages. As expected, treatment of RAW264.7 macrophages with LPS and nigericin significantly increased the release of NLRP3-inflammasome-dependent cytokines, including IL-1β p17, Caspase-1 p20, IL-1β and IL-18 (Fig. 2A, E, F). The mRNA and protein levels of METTL14 and NLRP3 in RAW264.7 macrophages were observably enhanced following stimulation with LPS whether combined with nigericin or not, indicating METTL14 may participate in the priming step (Fig. 2A–D). Both dot blot assay and colorimetric quantification showed that global m^6^A levels of total RNA in activated macrophages were notably elevated (Fig. 2G–I). Our findings pointed towards a possible role for METTL14-mediated m^6^A methylation in the process of NLRP3 inflammasome activation.

### Knocking down METTL14 inhibits the activation of NLRP3 inflammasome and alleviates lung injury in vitro and in vivo

**Fig. 3:**
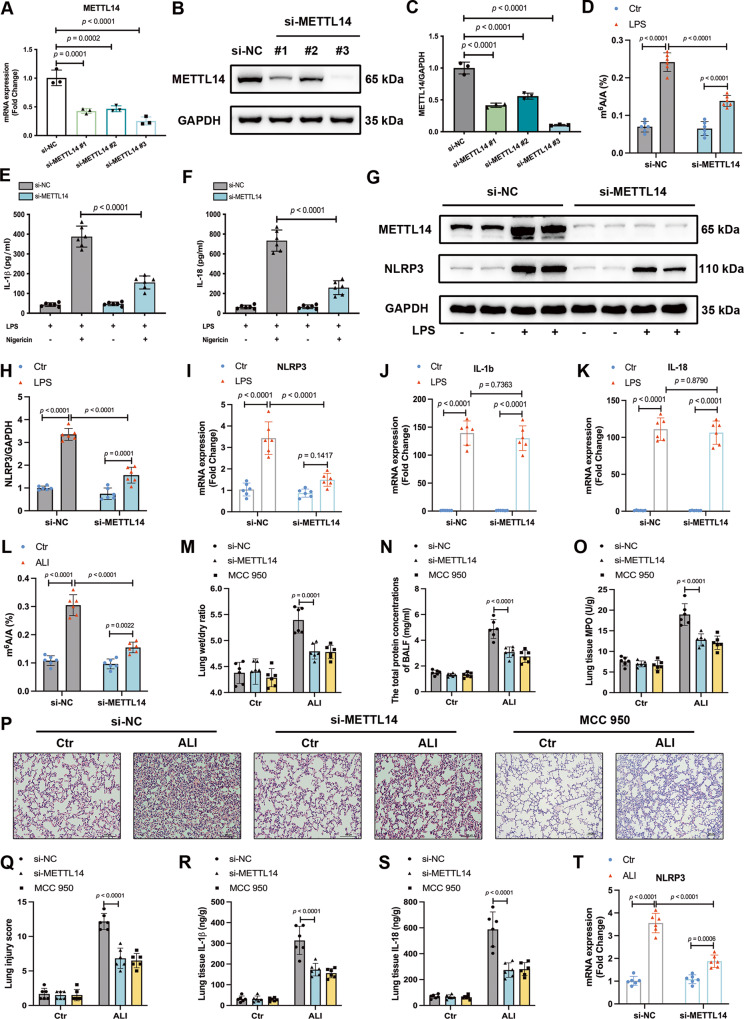
Knocking down METTL14 inhibits the activation of NLRP3 inflammasome and alleviates lung injury in vitro and in vivo. **A** RT-qPCR and (**B**, **C**) Western blot analysis of METTL14 in RAW264.7 cells following si-NC or si-METTL14 transfection to validated knockdown effect (*n* = 3). **D** A colorimetric assay measured the levels of m^6^A modification in RAW264.7 cells (*n* = 6). **E**, **F** The IL-1β and IL-18 concentrations in supernatants of si-NC and si-METTL14 RAW264.7 cells treated with or without nigericin for 30 min after LPS pretreatment for 6 h (*n* = 6). **G**, **H** Western blot analysis of NLRP3 in the indicated RAW264.7 cells (*n* = 6). **I**–**K** The mRNA expression of NLRP3, IL-1b, IL-18 were detected by RT-qPCR (*n* = 6). **L** The levels of m^6^A mRNA methylation in mouse lungs were measured by colorimetric assays (*n* = 6). **M** Pulmonary edema was assessed by lung wet/dry ratios (*n* = 6). **N** The total levels of protein in BALF. **O** Neutrophil accumulation measured by MPO activity assay in lung tissues. **P**, **Q** Representative H&E-stained lung sections (Scale bars, 100 μm) and histological injury score of each group. **R**, **S** The IL-1β, IL-18 concentrations in mouse lungs (*n* = 6) (**T**) NLRP3 mRNA expression in lung tissues (*n* = 6). All data are represented as mean ± SD. The unit for *n* is ‘replicates’(**A**–**K**) or ‘samples’ (**L**–**T**).

To determine the function of METTL14, we knocked down METTL14 expression in RAW264.7 macrophages using small interfering RNA (siRNA). Western blotting, real-time PCR, and colorimetric m^6^A quantification were used to verify the knockdown effect (Fig. 3A–D). Considering si-METTL14 #3 exhibited the superior knockdown efficiency, it was selected for further experiments. Compared with the negative control (si-NC) cells, knockdown of METTL14 exhibited a significant decrease in NLRP3 protein expression, as well as a reduced release of IL-1β and IL-18 cytokines in LPS-stimulated macrophages (Fig. 3E–H). Interestingly, METTL14 knockdown only downregulated the mRNA expression of NLRP3, but not that of IL-1b or IL-18 in LPS-treated cells (Fig. 3I–K). These results suggested that METTL14 may mediate NLRP3 inflammasome activation via regulating NLRP3.

We next employed METTL14 siRNA to determine the in vivo function of METTL14 in ALI. Total m^6^A levels in ALI mice were reduced after METTL14 was knocked down (Fig. 3L), suggesting that m^6^A modification occurring in ALI/ARDS was mainly mediated by METTL14. Both METTL14 siRNA and MCC950 (NLRP3 inhibitor) administration decreased the lung wet/dry ratio in ALI mice, indicating an alleviation of pulmonary edema associated with ALI/ARDS. (Fig. 3M). Compared with ALI group, the total protein concentrations in BALF and myeloperoxidase (MPO) activity in lung tissues of si-METTL14 + ALI and MCC950 + ALI group were notably lower (Fig. 3N, O). Similarly, H&E staining showed relatively intact alveolar structure and less inflammatory cell infiltration in si-METTL14 + ALI and MCC950 + ALI group than those in the ALI group (Fig. 3P, Q). Consistent with the in vitro results, we found that METTL14 knockdown inhibited the activation of NLRP3 inflammasome in ALI mice via regulating the mRNA levels of NLRP3, rather than IL-1b and IL-18 (Figs. 3R–T and S2A–D). Collectively, these results suggested that METTL14 knockdown may inhibit NLRP3 inflammasome activation and alleviate lung injury in vitro and in vivo, confirming that METTL14 plays a vital role in NLRP3 inflammasome activation in ALI/ARDS.

### Elevated METTL14 promotes the activation of NLRP3 inflammasome and aggravates lung injury in vitro and in vivo

**Fig. 4:**
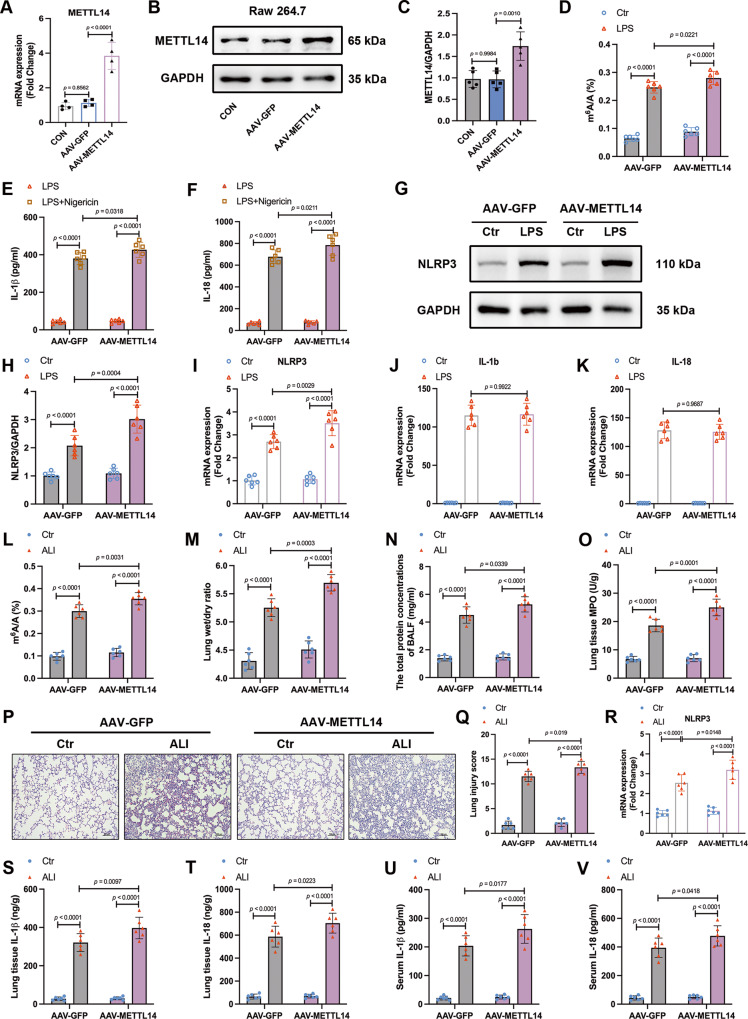
Elevated METTL14 promotes the activation of NLRP3 inflammasome and aggravates lung injury in vitro and in vivo. **A** RT-qPCR analysis (*n* = 4) and (**B**, **C**) Western blot analysis (*n* = 5) of METTL14 in RAW264.7 cells with or without METTL14 overexpression. **D** Quantification of m^6^A methylation in RAW264.7 cells (*n* = 6). **E**, **F** The IL-1β and IL-18 concentrations in supernatants of AAV-GFP and AAV-METTL14 RAW264.7 cells treated with or without nigericin for 30 min after LPS pretreatment for 6 h (*n* = 6). **G**, **H** Western blot analysis of NLRP3, and (**I**–**K**) RT-qPCR analysis of NLRP3, IL-1b, IL-18 in the indicated RAW264.7 cells (*n* = 6). **L** Quantification of m^6^A methylation in mouse lungs (*n* = 6). **M** Wet/dry ratios of mouse lungs were assessed (*n* = 6). **N**, **O** The levels of total protein in BALF and MPO activity in lung were assessed by biochemical kits. **P**, **Q** Representative H&E-stained sections (Scale bars, 100μm) and histological injury score of mouse lungs. **R** NLRP3 mRNA expression in lung tissues (*n* = 6). **S**–**V** The IL-1β, IL-18 concentrations in mouse lungs and serum (*n* = 6). All data are represented as mean ± SD. The unit for n is ‘replicates’(**A**–**K**) or ‘samples’ (**L**–**V**).

To further elucidate the function of METTL14 in ALI, we performed gain-of-function assay by overexpressing METTL14 in RAW264.7 macrophages (Fig. 4A–C). METTL14 overexpression in RAW264.7 macrophages increased m^6^A levels (Fig. 4D) and activated NLRP3 inflammasome in macrophages by upregulating the mRNA levels of NLRP3, rather than IL-1b and IL-18 (Fig. 4E–K). We subsequently explored the in vivo function of METTL14 in ALI using AAV9 that expressed full-length METTL14. AAV-GFP was used as a control. As shown in Fig. 4L, a marked increase in the level of m^6^A modification was detected in lung tissue of ALI mice. AVV-METTL14 aggravated pulmonary edema of ALI mice, as revealed by lung wet/dry ratio (Fig. 4M). METTL14 overexpression also increased the total protein concentrations in BALF and MPO activity (Fig. 4N, O). Likewise, H&E staining showed thicker alveolar walls and more inflammatory infiltration in AVV-METTL14 + ALI group than those in the ALI group (Fig. 4P, Q). Indeed, METTL14 activated NLRP3 inflammasome via upregulating the mRNA levels of NLRP3 (Fig. 4R–V). Taken together, these data supported that the elevation of METTL14 contributes to the activation of the NLRP3 inflammasome and the exacerbation of lung injury in vitro and in vivo.

### NLRP3 is the direct target of METTL14-mediated m^6^A modification

**Fig. 5:**
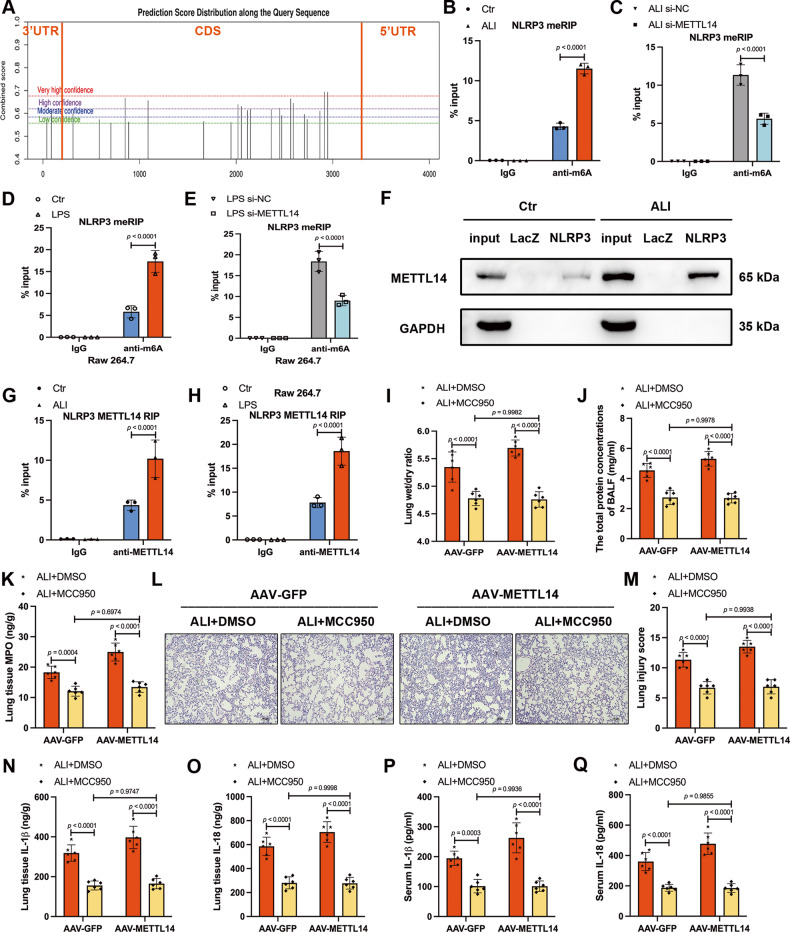
NLRP3 is the direct target of METTL14-mediated m6A modification. **A** Potential targeted m^6^A sites in NLRP3 mRNA according to SRAMP online website. **B**, **C** MeRIP analysis of m^6^A modified NLRP3 mRNA in (**B**) Ctr and ALI mice (*n* = 3), and (**C**) ALI mice with or without METTL14 knockdown (*n* = 3). **D**, **E** m^6^A enrichment of NLRP3 mRNA in (**D**) Ctr and LPS-treated RAW264.7 cells (*n* = 3), and (**E**) LPS-treated RAW264.7 cells with or without METTL14 knockdown (*n* = 3). **F** RNA pull-down assay was performed in ALI and Ctr mice, and the METTL14 pulled down by NLRP3 RNA probe in mouse lungs were detected by western blot, LacZ RNA probe served as the RNA control (*n* = 3). **G**, **H** RT-qPCR analysis of RIP assays showed the direct binding between the METTL14 protein and NLRP3 mRNA in (**G**) lung tissues of Ctr and ALI mice (*n* = 3), and (**H**) Ctr and LPS-treated RAW264.7 cells (*n* = 3). **I** Lung wet/dry ratios was used to assess lung fluid content (*n* = 6). **J** The concentrations of total protein in BALF and (**K**) MPO activity were detected in mouse lung. **L**, **M** Presentative H&E-stained sections (Scale bars, 100μm) and histological injury score for each group of mice were shown. **N**–**Q** The IL-1β, IL-18 concentrations in mouse lungs and serum (*n* = 6). All data are represented as mean ± SD. The unit for n is ‘replicates’ (**D**, **E**, **H**) or ‘samples’ (**B**, **C**, **F**, **G**, **I**–**Q**).

NLRP3 is present in low concentrations under resting conditions, which is insufficient to activate the inflammasome [11]. Based on previous results showing that METTL14 regulated the mRNA expression of NLRP3 both in vivo and in vitro, we surmised that NLRP3 may be a direct target of METTL14. To validate the role of m^6^A methylation modulated by METTL14 in NLRP3 transcript, we analyzed potential m^6^A targeting motifs using SRAMP (http://www.cuilab.cn/sramp). A total of 24 RRACH m^6^A-binding motifs were identified in NLRP3 mRNA sequence (Fig. 5A and Supplemental Table 3). The m^6^A RNA immunoprecipitation (MeRIP) assays confirmed that NLRP3 mRNA m^6^A modification was enhanced in ALI mice and LPS-treated macrophages (Fig. 5B, D), but significantly decreased after METTL14 knockdown (Fig. 5C, E). Next, we used RNA pull-down and RNA immunoprecipitation (RIP) assays to determine whether there is a direct interaction between NLRP3 mRNA and METTL14. RNA pull-down assays showed that METTL14 interacts with the NLRP3 transcript, and that this interaction was enhanced in ALI mice (Fig. 5F). RIP analysis with the METTL14 antibody further confirmed the interaction between METTL14 and NLRP3 mRNA both in vivo and in vitro (Fig. 5G, H). Moreover, rescue assays were performed by using MCC950, an NLRP3 inhibitor, in AAV-METTL14 mice. Our data revealed that the extent of lung injury in AAV-METTL14 ALI mice was restored by MCC950 treatment, as revealed by lung wet/dry ratio, BALF protein content, MPO activity and histological injury score (Fig. 5I–M). The over-release of IL-1β and IL-18 in lung tissues and serum in AAV-METTL14 ALI mice was reversed by MCC950 treatment (Fig. 5N–Q). These findings indicated that NLRP3 is a direct and functional target of METTL14-mediated m^6^A modification during NLRP3 inflammasome activation in ALI/ARDS.

### IGF2BP2 is upregulated and enhances the stability of NLRP3 mRNA in ALI

**Fig. 6:**
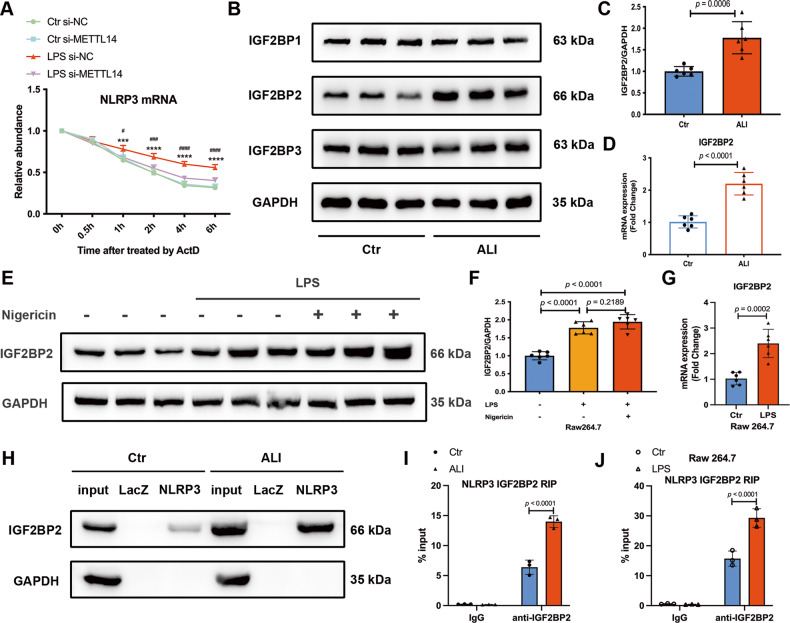
IGF2BP2 is upregulated and interacts with NLRP3 mRNA both in vivo and in vitro during ALI. **A** METTL14 siRNA reduced NLRP3 stability in LPS-treated RAW264.7 macrophages. Decay rate of NLRP3 mRNA in si-NC or si-METTL14 transfected RAW264.7 cells treated with or without LPS following actinomycin D (5 μg/ml) treatment (LPS si-NC compared with Ctr si-NC: ***P < 0.001, ****P < 0.0001; LPS si-METTL14 compared with LPS si-NC: ^#^*P* < 0.05, ^###^*P* < 0.001, ^####^*P* < 0.0001, *n* = 3). **B**, **C** Western blot analysis of m^6^A readers (IGF2BP1-3) in lung tissues of ALI and Ctr mice (*n* = 6). **D** RT-qPCR analysis of IGF2BP2 in lung tissues of ALI and Ctr mice (*n* = 6). **E**, **F** Western blot and (**G**) RT-qPCR analysis of IGF2BP2 in RAW264.7 cells (*n* = 6). **H** RNA pull-down assay was performed in lung tissues of ALI and Ctr mice, and the IGF2BP2 pulled down by NLRP3 RNA probe was significantly elevated in ALI mice, LacZ RNA probe was set as the RNA control (*n* = 3). RT-qPCR analysis of RIP assays in (**I**) lung tissues of ALI and Ctr mice and (J) LPS-treated RAW264.7 cells, showing the direct binding between the IGF2BP2 protein and NLRP3 mRNA was significantly increased during ALI (*n* = 3). All data are represented as mean ± SD. The unit for n is ‘replicates’ (**E**–**G**, **J**) or ‘samples’ (**A**-**D**, **H, I**).

Considering that METTL14 induces NLRP3 mRNA m^6^A methylation and that the loss of m^6^A in NLRP3 mRNA mediated by METTL14 knockdown leads to a decrease in NLRP3 mRNA and protein expression in ALI mice, we sought to determine whether METTL14-mediated m^6^A modification affects the NLRP3 mRNA stability. We treated RAW264.7 macrophages with the transcription inhibitor actinomycin D (ActD) and found that NLRP3 decay in si-METTL14-treated macrophages was faster than that in corresponding controls when stimulated with LPS (Fig. 6A), suggesting that METTL14 regulates NLRP3 expression via an m^6^A-dependent mRNA decay mechanism. Therefore, we identified m^6^A readers that may participate in the regulation of NLRP3 mRNA stability. The IGF2BP family regulates the stability of methylated mRNA by acting as m^6^A readers [23]. First, we detected the protein expression of IGF2BP1, IGF2BP2, and IGF2BP3 using western blotting. We found that protein expression of IGF2BP2 was distinctly upregulated in ALI mice (Fig. 6B, C), which was consistent with its mRNA expression (Fig. 6D). Similarly, the protein and mRNA expression levels of IGF2BP2 were notably augmented in LPS-treated RAW264.7 macrophages (Fig. 6E–G). To further validate the direct interaction between NLRP3 mRNA and IGF2BP2, we performed an in vivo RNA precipitation assay using a biotinylated NLRP3 probe. RNA pull-down assay detected that specific binding of IGF2BP2 was enhanced in ALI mice (Fig. 6H). RIP analysis with the IGF2BP2 antibody further confirmed that their interaction was facilitated in vivo and in vitro during ALI (Fig. 6I, J). These results implied that the stability of NLRP3 mRNA might be regulated by IGF2BP2 via METTL14-mediated m^6^A modification.

### IGF2BP2 knockdown decreases NLRP3 mRNA stability and inhibits NLRP3 inflammasome activation in LPS-activated macrophages

**Fig. 7:**
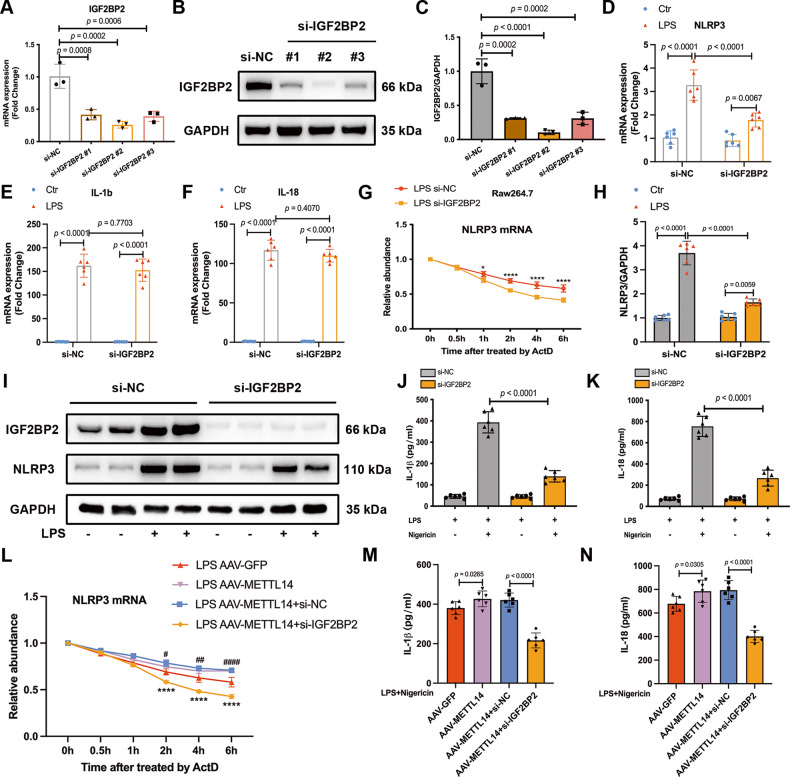
IGF2BP2 knockdown decreases NLRP3 mRNA stability and inhibits NLRP3 inflammasome activation in LPS-activated macrophage. **A** RT-qPCR analysis and (**B**, **C**) Western blot analysis of IGF2BP2 in RAW264.7 following si-NC or si-IGF2BP2 transfection to verify the knockdown effect (*n* = 3). **D**–**F** The mRNA expression of NLRP3, IL-1b, IL-18 in RAW264.7 cells by RT-qPCR (*n* = 6). **G** Decay rate of NLRP3 mRNA in LPS-treated RAW264.7 with or without IGF2BP2 knockdown following actinomycin D (5 μg/ml) treatment (*P < 0.05, ***P < 0.001, ****P < 0.0001). **H**, **I** Western blot analysis of NLRP3 in RAW264.7 treated with or without LPS after siRNA transfection (*n* = 6). **J**, **K** The IL-1β and IL-18 concentrations in supernatants of si-NC or si-IGF2BP2 transfected RAW264.7 cells treated with or without nigericin for 30 min after LPS pretreatment for 6 h (*n* = 6). **L** Decay rate of NLRP3 mRNA, and (**M**, **N**) the IL-1β and IL-18 concentrations in AAV-GFP and AAV-METTL14 LPS-treated RAW264.7 cells with or without IGF2BP2 knockdown (*n* = 6). The graphs depict mean ± SD, *n* = 6 replicates of each condition.

To examine whether IGF2BP2 regulates NLRP3 expression, we used siRNA to knockdown IGF2BP2 in RAW264.7 macrophages (Fig. 7A–C), and si-IGF2BP2 #2 with the best knockdown effect was selected for further experiments. As shown, IGF2BP2 knockdown significantly downregulated the mRNA expression of NLRP3 (Fig. 7D), but not IL-1b (Fig. 7E) or IL-18 (Fig. 7F), in LPS-treated RAW264.7 macrophages. To further determine the mechanism underlying IGF2BP2-induced regulation of NLRP3, we examined the effect of IGF2BP2 knockdown on the lifetime of NLRP3 mRNA. We found that the stability of NLRP3 mRNA in LPS-treated RAW264.7 macrophages was reduced by IGF2BP2 knockdown (Fig. 7G). As expected, IGF2BP2 knockdown inhibited NLRP3 expression (Fig. 7H, I) and the release of IL-1β and IL-18 (Fig. 7J, K) in LPS-treated RAW264.7 macrophages. We proceeded to silence IGF2BP2 in METTL14-overexpressing cells. Our data showed that IGF2BP2 knockdown reduced NLRP3 mRNA lifespan (Fig. 7L) and restored the over-release of IL-1β and IL-18 (Fig. 7M, N) in METTL14-overexpressing macrophages. These results confirmed that IGF2BP2 participates in METTL14-mediated NLRP3 inflammasome activation by enhancing the stability of NLRP3 mRNA in macrophages.

### Knocking down IGF2BP2 inhibits the activation of NLRP3 inflammasome and alleviates lung injury in ALI mice

**Fig. 8:**
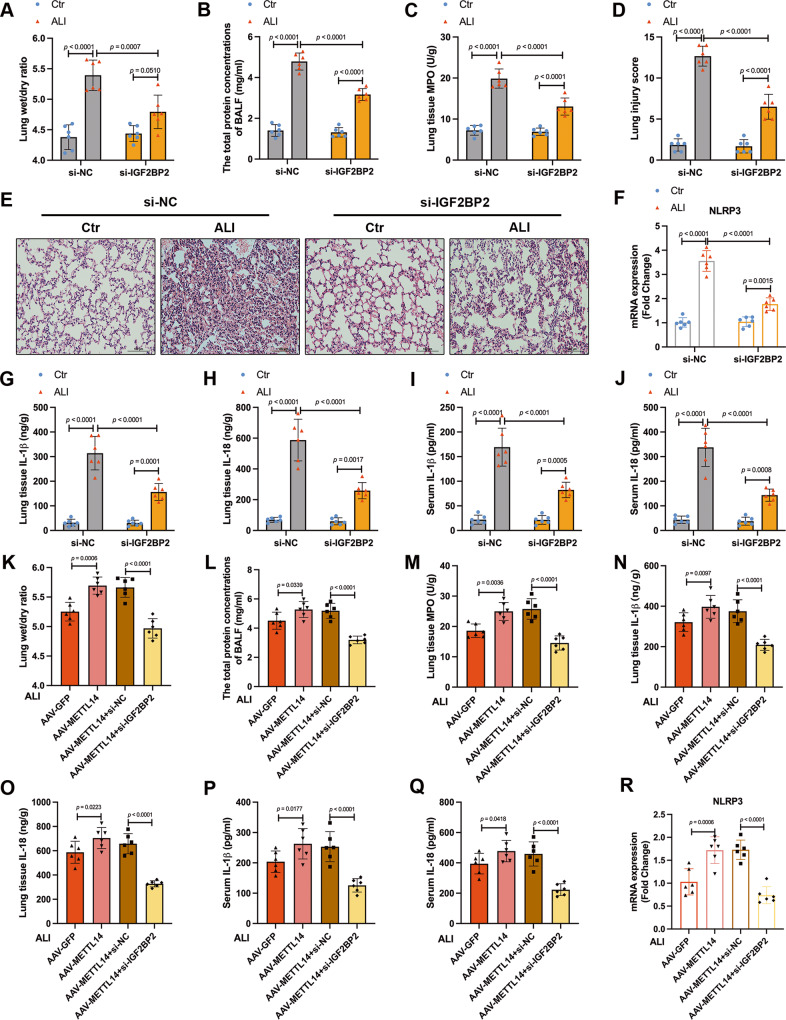
Knocking down IGF2BP2 inhibits the activation of NLRP3 inflammasome and alleviates lung injury in ALI mice. **A** Lung wet/dry ratios, (**B**) total protein content in BALF, and (**C**) MPO activity were detected in Ctr and ALI mice treated with si-NC or si-IGF2BP2 (*n* = 6). **D** Lung injury score and (**E**) presentative images from H&E staining-sections of lung tissues were shown (Scale bars, 100μm) (*n* = 6). **F** RT-qPCR analysis of NLRP3 mRNA expression (*n* = 6). **G**, **I** The IL-1β and (**H**, **J**) IL-18 concentrations in lung tissue and serum of Ctr and ALI mice treated with si-NC or si-IGF2BP2 is shown (*n* = 6). **K** Lung wet/dry ratios, (**L**) total protein content in BALF, and (**M**) MPO activity were detected in AAV-GFP and AAV-METTL14 ALI mice with or without IGF2BP2 knockdown (*n* = 6). The IL-1β and IL-18 concentrations in (**N**, **O**) lung tissue and (**P**, **Q**) serum were measured by ELISA (*n* = 6). **R** RT-qPCR analyzed NLRP3 mRNA expression level. All data are represented as mean ± SD, *n* = 6 mice per group.

We further determined the therapeutic potential of IGF2BP2 against mouse ALI models by applying siRNA to knock down IGF2BP2 in vivo. Compared to mice treated with control siRNA, the si-IGF2BP2 group showed significantly alleviated lung wet/dry ratio in ALI mice (Fig. 8A). IGF2BP2 inhibition also decreased the total protein levels in BALF and MPO activity in ALI lung (Fig. 8B, C). Similar effects of IGF2BP2 knockdown on alleviating lung injury in ALI mice were revealed H&E staining (Fig. 8D, E). Disruption of IGF2BP2 downregulated the mRNA expression of NLRP3 in the lung tissues of ALI mice (Fig. 8F). The dramatic increase in the IL-1β and IL-18 levels were efficiently diminished in ALI mice after treated with si-IGF2BP2 (Fig. 8G–J). We further performed IGF2BP2 inhibition in AAV-METTL14 mice. The deterioration of lung wet/dry ratio, BALF protein content, MPO activity in AAV-METTL14 ALI mice were restored by IGF2BP2 knockdown (Fig. 8K-M). IGF2BP2 inhibition also reduced the upregulation of IL-1β and IL-18 levels in lung tissues and serum (Fig. 8N–Q) and NLRP3 mRNA (Fig. 8R) in AAV-METTL14 ALI mice. These results manifested that IGF2BP2 knockdown may relieve ALI via inhibiting the NLRP3 inflammasome activation in vivo.

## Discussion

In this study, we discovered that the contents of m^6^A and METTL14 in lung tissues of ALI mice subjected to LPS were enhanced. METTL14-mediated NLRP3 mRNA m^6^A modification increases NLRP3 mRNA stability in injured lungs in an IGF2BP2-dependent manner. Thus, knocking down METTL14 or IGF2BP2 may play a protective role in ALI by inhibiting NLRP3 inflammasome activation. This finding may provide new pathophysiological insights into potential therapeutic strategies for ALI/ARDS.

N^6^-Methyladenosine (m^6^A) is the most abundant epigenetic mRNA modification and exerts different biological effects in various diseases via post-transcriptional regulation [24–26]. Emerging evidence indicates that m^6^A may play an indispensable role in some inflammatory diseases [27]. RNA m^6^A modification is mediated by m^6^A writers (methyltransferases), erasers (demethylases), and readers. METTL14, a key component of the m^6^A methyltransferase complex, stabilizes the structure of METTL3 and enhances its enzymatic activity by binding to RNA, which ultimately increases m^6^A level [28]. Our study showed that m^6^A modification and the m^6^A methyltransferase METTL14 were increased in ALI mice. Further analysis confirmed METTL14 is mainly elevated in recruited circulating monocyte-derived macrophages of ALI mice. Interestingly, some studies have shown that neutrophil extracellular traps (NETs) induced ferroptosis in alveolar epithelial cells of cecal ligation and puncture (CLP)-mouse model by activating METTL3, while a few studies showed METTL3-mediated m^6^A modification alleviated ALI via inhibiting endothelial injury, indicating that m^6^A may exert different effects on ALI/ARDS owing to cell types and challenges [29–31].

Alveolar macrophages (AMs) consist of two subpopulations, including resident AMs and recruited AMs [32]. The resident AMs serve as an immunosuppressive subpopulation and mainly present the M2 phenotype, whereas the recruited AMs, which are derived from circulating monocytes, prefer to differentiate into pro-inflammatory M1 phenotype [33, 34]. Consistent with the enrichment of METTL14 in recruited macrophages, we found the m^6^A levels and METTL14 expression were also increased in a RAW264.7 macrophage NLRP3 inflammasome activation model. This finding was in line with recent studies showing that METTL14 activated M1 polarization of macrophages in ischemic stroke and coronary heart disease, indicating that METTL14 may play a vital role in the functional regulation of macrophages [35, 36].

Uncontrolled inflammatory responses mediated by pulmonary macrophages are indeed crucial in the pathogenesis of ALI/ARDS [37]. In the present study, we found that METTL14 knockdown alleviated lung injury via inhibiting NLRP3 inflammasome activation in macrophages, consistent with the result in sepsis-associated myocardial dysfunction [38]. The NLRP3 inflammasome, which acts as the core of the inflammatory response, mediates caspase-1 activation and the secretion of proinflammatory cytokines, IL-1β/IL-18 [39]. Enhanced activation of the NLRP3 inflammasome in alveolar macrophage is involved in the pathogenesis of ALI/ARDS caused by various pathogenic factors [40, 41]. Inhibition NLRP3 inflammasome using the specific inhibitor MCC950 has achieved satisfactory therapeutic results not only in ALI model but also other inflammatory conditions including autoimmune diseases [42]. However, the liver toxicity of MCC950 was found in a phase II clinical trial for rheumatoid arthritis, which casts a shadow over the future clinical application of MCC950 [43]. In our study, we found that the protective effects of METTL14 knockdown were similar to MCC950 in ALI. Therefore, it is promising to develop a specific inhibitor of METTL14 for treatment on ALI/ARDS and other inflammatory diseases.

Although some studies have revealed an association between METTL14 and NLRP3 inflammasome activation [44, 45], whether METTL14 plays a direct role in regulating NLRP3 expression remains unclear. NLRP3 in low concentrations is inadequate for initiating inflammasome activation under resting conditions [9]. Our results revealed that METTL14 knockdown markedly downregulated the mRNA expression of NLRP3, but not that of IL-1b or IL-18, both in vivo and in vitro. Therefore, we suspected that NLRP3 mRNA may be the m^6^A methylation target of METTL14 in ALI/ARDS. Hence, we analyzed potential m^6^A targeting motifs in SRAMP and identified 24 RRACH m^6^A-binding motifs in NLRP3 mRNA. We further confirmed that the loss of METTL14 abolished the increase in m^6^A methylation levels of NLRP3 mRNA in ALI mice and RAW264.7 macrophages treated with LPS. RNA pull-down and RIP assays confirmed that METTL14 directly interacted with NLRP3 mRNA, and that such binding was enhanced in ALI mice. These results indicated that NLRP3 mRNA is a direct m^6^A methylation target of METTL14 during NLRP3 inflammasome activation in ALI/ARDS.

The biological functions of m^6^A modifications rely on m^6^A readers, which regulate RNA metabolism, including translation, splicing, export, and degradation [16]. Elevated m^6^A modification mediated by METTL14 increases NLRP3 mRNA and protein expression in ALI mice. Furthermore, an ActD RNA stability assay showed that the half-life of NLRP3 transcripts had decreased following METTL14 knockdown, indicating that NLRP3 expression was modulated via an m^6^A-dependent mRNA decay mechanism. The m^6^A readers, IGF2BP1/2/3, are involved in regulating the stability of methylated mRNA [46]. Based on our data indicating that only IGF2BP2 was upregulated in ALI mice and LPS-treated RAW264.7 macrophages, we hypothesized that IGF2BP2 may act as the potential binding protein of NLRP3 mRNA via an m^6^A-dependent mRNA decay mechanism. Indeed, RNA pull-down and RIP assays confirmed that IGF2BP2 directly binds to NLRP3 transcripts. Moreover, our findings suggested that IGF2BP2 knockdown may decrease the NLRP3 mRNA stability and inhibit NLRP3 inflammasome activation in ALI mice and LPS-treated RAW264.7 macrophages, thereby alleviating lung injury. Collectively, these results suggest that IGF2BP2 specifically binds to the NLRP3 transcripts and enhances NLRP3 mRNA stability in an m^6^A-dependent manner during ALI/ARDS.

This study has some limitations. We investigated the role of METTL14/IGF2BP2 in NLRP3 inflammasome activation in ALI mice and RAW264.7 macrophages. However, the role of METTL14/IGF2BP2 in clinical patients of ARDS remains to be elucidated. In addition, although we verified that m^6^A modification of NLRP3 mRNA was mediated by METTL14, the specific motif of NLRP3 transcripts methylated by METTL14 has not yet been elucidated and may have to be confirmed in future research. Third, although we found that METTL14 and IGF2BP2 were upregulated in ALI mice and RAW264.7 macrophages, upstream mechanisms underlying this process have not yet been explored and require further investigation via a follow-up study.

Overall, our study provides robust in vitro and in vivo evidence supporting the critical roles of METTL14/IGF2BP2 in NLRP3 inflammasome activation during ALI/ARDS. Mechanistically, METTL14-catalyzed NLRP3 mRNA m^6^A methylation enhances the stability of NLRP3 mRNA in an IGF2BP2-dependent manner in ALI/ARDS. Our findings indicate that METTL14/IGF2BP2 shows potential as therapeutic targets in the treatment of ALI/ARDS.

## Materials and methods

### Animals

Male specific-pathogen-free C57/BL6 mice (8–10 weeks old) were obtained from the Guangdong Medical Laboratory Animal Center (Guangzhou, China). All mice were housed under controlled, pathogen-free conditions at the Laboratory Animal Center of Sun Yat-sen University Cancer Center. All animals were housed in separate cages in a temperature- (24 ± 1 °C) and humidity-controlled (50–60%) room under a 12/12-h light/dark cycle. All experiments were conducted in accordance with the guidelines defined by the Sun Yat-sen University Cancer Center. The study was approved by the Animal Care and Ethics Committee of Sun Yat-sen University Cancer Center (Permit Number: 2021-000043).

### Animal models and treatments

A mouse LPS-induced ALI model was established as previously described [47]. Briefly, mice from the ALI groups were treated with a single intraperitoneal dose of 15 mg/kg LPS obtained from Escherichia coli 055: B5 (Sigma-Aldrich, St. Louis, MO, USA) in saline, whereas mice injected with the same volume of saline served as controls. After 24 h, the mice were killed and the lung lobes were harvested for further analysis. This in vivo study was performed via six series of experiments. Mice in the first series were randomly divided into control and ALI groups. Mice in the second series were randomly assigned to receive the following treatments: (1) control + si-NC, (2) control + si-METTL14, (3) control + MCC950, (4) ALI + si-NC, (5) ALI + si-METTL14 and (6) ALI + MCC950 (an NLRP3 inflammasome inhibitor, 50 mg/kg, i.p., Selleck, Shanghai, China). Mice in the third series were grouped as follows: (1) control + si-NC, (2) control + si-IGF2BP2, (3) ALI + si-NC, or (4) ALI + si-IGF2BP2. Each group received a dose of 20 nmol siRNA (either si-NC, si-METTL14 or si-IGF2BP2) in 200 μl of saline via the tail vein 2 d before being challenged with LPS or saline. Mice in the fourth series were grouped as follows: (1) control + AAV-GFP, (2) control + AAV-METTL14, (3) ALI + AAV-GFP, or (4) ALI + AAV-METTL14. Each group received a dose of 50 μl viral solution (either AAV-GFP or AAV-METTL14 with 10^12^ vg/ml titer) via intranasal instillation 4 weeks before being challenged with LPS or saline. Mice in the fifth series were grouped as follows: (1) ALI + AAV-GFP + DMSO, (2) ALI + AAV-METTL14 + DMSO, (3) ALI + AAV-GFP + MCC950, or (4) ALI + AAV-METTL14 + MCC950. Mice in the sixth series were grouped as follows: (1) ALI + AAV-GFP, (2) ALI + AAV-METTL14, (3) ALI + AAV-METTL14 + si-NC, or (4) ALI + AAV-METTL14 + si-IGF2BP2. The treatments of each group were performed according to the mentioned above.

### Histopathological analysis

Left lung lobes were fixed in 4% paraformaldehyde for 48 h, dehydrated, embedded in paraffin, and sliced into 5-µm-thick sections. The sections were then stained with hematoxylin and eosin (H&E) according to the manufacturer’s instructions, to evaluate lung histopathology. The damage to the lung tissues was scored using a previously described semiquantitative scoring system [47]. Images were captured using a microscope (NIKON Eclipse Ni-U; NIKON, Tokyo, Japan).

### Lung wet/dry ratio

Any blood present on the isolated right lungs was blotted with filter paper before the weights of these lungs were recorded as wet weight. Then, the lungs were then stored in an incubator at 60 °C for 48 h, following which the weight of the lungs was recorded as dry weight. The lung wet/dry (W/D) ratio was used to evaluate the degree of pulmonary edema.

### Bronchoalveolar lavage

At the time of lavage, the mice were anesthetized with an i.p. injection of 1% pentobarbital sodium (50 mg/kg). The chest cavity was exposed, then the trachea was intubated, and a whole lung lavage was performed by employed sterile PBS (1 mL). The collected lavage fluid was centrifuged at 1000×*g* for 10 min at 4 °C, and the cell-free supernatants were harvested and stored at −80^◦^C for further analysis. The total protein concentration of bronchoalveolar lavage fluid (BALF) was measured using the BCA Protein Assay Kit (Thermo Fisher Scientific).

### Cell culture and treatments

RAW264.7 cells, a mouse macrophage cell line, was obtained from ATCC and cultured in Dulbecco’s modified Eagle’s medium (Gibco from Thermo Fisher Scientific, Waltham, MA, USA) with 10% fetal bovine serum (Gibco) in an incubator at 37 °C and 5% CO_2_. To establish an NLRP3 inflammasome activation model in vitro, RAW264.7 cells were stimulated with LPS (1 μg/mL) for 6 h, then treated with nigericin (10 μM, InvivoGen, San Diego, CA, USA) for 30 min.

For transient transfection purpose, cells were seeded at 30–40% confluence and cultured overnight, following which si-METTL14, si-IGF2BP2, and negative control (si-NC) were diluted in Opti-MEM^®^ medium (Thermo Fisher Scientific, Waltham, MA, USA) and transfected using Lipofectamine 3000 transfection reagent (Invitrogen, Carlsbad, CA, USA) according to the manufacturer’s instructions. After 48 h of transfection, the cells were treated with or without LPS (1 μg/mL) for 6 h. Three siRNA sequences targeting METTL14 and IGF2BP2 were designed and synthesized by RiboBio (Guangzhou, China) and these were listed in Supplemental Table 1. Specific siRNA with the best knockdown effect was used for further research and in vivo assays.

### ELISA analysis and myeloperoxidase activity

Mice were anesthetized with an intraperitoneal injection of 1% pentobarbital sodium (50 mg/kg). Blood samples were collected from the retro-orbital sinus after the mice lost consciousness. Subsequently, blood samples were allowed to clot by leaving them undisturbed at 25 °C for 30 min. The clots were then removed to obtain serum via centrifugation at 1000 × g for 10 min at 4 °C. Part of the right lung from each mouse was homogenized with ELISA buffer and centrifuged to obtain lung tissue supernatants. Samples of murine serum, lung tissue supernatants, and cell culture supernatants were used to quantify the concentrations of IL-1β (R&D System, Minneapolis, MN, USA) and IL-18 (R&D System) by using murine ELISA kits, according to the manufacturer’s instructions. The MPO activity in the lung tissue was assessed by an MPO assay kit (R&D System).

### RNA m^6^A dot blot assay

Total RNA and poly-A RNA were isolated from the lung tissue or RAW264.7 macrophages using a RNeasy mini kit (Qiagen, Düsseldorf, Germany) and a Dynabeads^®^ mRNA purification kit (Ambion, Austin, TX, USA), according to the manufacturer’s instructions. RNA was quantified using a Nanodrop, and equal amounts of RNA were crosslinked onto Hybond-N discs (Cytiva, USA) using a UV crosslinker (Spectroliner, Long Island, NY, USA). The membrane was quickly washed and blocked using 5% nonfat dry milk in 0.1% phosphate-buffered saline with Tween-20 (PBST) supplemented with RNaseOUT (Thermo Fisher Scientific). The membrane was incubated overnight at 4 °C with rabbit anti-m^6^A antibody (1:500, cat#A-1802-100, EpiGentek, Farmingdale, NY, USA), followed by incubation with horseradish peroxidase (HRP)-conjugated secondary anti-rabbit antibody. Membranes were washed and visualized using an enhanced chemiluminescence detection system. Images were acquired using a ChemiDoc™ Touch Imaging System (Bio-Rad, Berkeley, CA, USA). Finally, the membranes were stained with methylene blue as a loading control. The signal intensity of the dot blot was analyzed using ImageJ software (NIH, Bethesda, MD, USA).

### RNA m^6^A modification quantification

The levels of m^6^A in lung tissues and RAW264.7 macrophages were quantified using an EpiQuik m^6^A RNA Methylation Quantification Kit (EpiGentek), according to the manufacturer’s recommendations.

### Western blotting

Mouse lung tissues or cells were lysed using RIPA lysis buffer (Beyotime, Shanghai, China) containing a protein inhibitor cocktail (Roche, Mannheim, Baden Württemberg, Germany). The total protein concentration was quantified using a BCA kit (Thermo Fisher Scientific). Samples were denatured at 100 °C for 10 min and separated on 10–12% SDS-PAGE gels with a molecular weight standard. Proteins from SDS-PAGE gel were transferred onto PVDF membranes (Merck Millipore, Darmstadt, Germany), blocked using 5% non-fat milk for 2 h, and incubated at 4 °C with the following primary antibodies overnight: METTL3 (1:1000, 15073-1-AP, Proteintech, Wuhan, China), METTL14 (1:1000, A8530, Abclonal, Boston, MA, USA), METTL16 (1:1000, 17676 S, Cell Signaling Technology, Danvers, MA, USA), WTAP (1:1000, 60188-1-Ig, Proteintech), FTO (1:1000, 45980 S, Cell Signaling Technology), ALKBH5 (1:1000, 16837-1-AP, Proteintech), NLRP3 (1:1000, AG-20B-0006-C100, AdipoGen, San Diego, CA, USA), Caspase-1 (1:1000, AG-20B-0042-C100, AdipoGen), IL-1β (1:500, AF-401-NA, R&D System), IGF2BP1 (1:1000, A1517, Abclonal), IGF2BP2 (1:1000, 11601-1-AP, Proteintech), IGF2BP3 (1:1000, A23295, Abclonal). After three washes, the membranes were incubated with the corresponding HRP-conjugated secondary antibody (1:1000, Abcam, Cambridge, UK) at room temperature for 1 h. Protein bands were detected using ECL and visualized using a ChemiDoc™ Touch Imaging System (Bio-Rad). The band intensities were analyzed by using ImageJ software.

### Immunofluorescence

Left lung lobes were fixed with 4% paraformaldehyde for 48 h, dehydrated, embedded in paraffin, and sectioned into 5-µm slices. After deparaffinated, dehydration, and antigen recovery, sections were incubated in blocking solution (Beyotime) for 1 h at room temperature and then incubated with primary antibodies overnight at 4 °C, followed by incubation at 25 °C for 1 h with fluorescently labeled secondary antibodies. Nuclei were stained for 10 min with DAPI. Images from six representative non-overlapping high-power fields (HPFs) of individual mice were taken on a fluorescent microscope (Leica, Wetzlar, Germany) in a blinded manner. The following antibodies were used: anti-METTL14 (1:200, A8530, Abclonal), anti-CD68 (18985-1-AP, 1:100, Proteintech), anti-F4/80 (18985-1-AP, 1:100, Proteintech), anti-Siglec F (18985-1-AP, 1:100, Proteintech), and anti-CK7 (16001- 1-AP, 1:100; Proteintech).

### Quantitative real-time RT-PCR

Total RNA was extracted using TRIzol reagent (Invitrogen), and subsequently reverse-transcribed to cDNA according to the instructions of manufacturer of the qPCR transcription kit (EZ Bioscience, Roseville, NM, USA). Quantitative PCR was performed using SYBR Green Mix (EZ Bioscience) and a CFX96 Real-Time PCR Detection System (Bio-Rad). Target mRNA expression was calculated via the 2^-ΔΔCt^ method using GAPDH as an endogenous control. Primer sequences are listed in Supplemental Table 2.

### RNA stability assay

RAW264.7 macrophages were cultured in six-well culture plates until they reached 80% confluence. Actinomycin D (Abmole, Houston, TX, USA) was added at a final concentration of 5 μg/ml. Cells were collected at 0, 0.5, 1, 2, 4, and 6 h. Total RNA was extracted, RT–qPCR was performed as described above, and GAPDH was used as the loading control for normalization. Then, the RNA half-life was calculated.

### RNA immunoprecipitation assay

RIP was performed according to the manufacturer’s instructions (Merck Millipore). Briefly, lung tissues of equal weight were mechanically sheared into a single-cell suspension using a homogenizer and resuspended in RIP lysis buffer containing protease inhibitor and RNase inhibitors. The mixture was centrifuged at 14 000 rpm at 4 °C for 10 min to obtain the supernatant, which was then divided into three fractions: Input, IP, and IgG. Each fraction was incubated overnight with the corresponding primary antibody at 4 °C, followed by protein A/G bead incubation at room temperature for 30 min. After six washes, the beads were incubated with 150 μl proteinase K buffer at 55 °C for 30 min. Total RNA was extracted, and the expression of related genes was detected via RT-qPCR.

### m^6^A RNA Immunoprecipitation assay

The MeRIP assay was performed using a Magna MeRIP m^6^A Kit (Merck Millipore). Briefly, total RNA was extracted from lung tissues and RAW264.7 macrophages using TRIzol reagent. Approximately 20 μg of purified RNA was incubated with RNA fragmentation buffer. Then, 1 μg of the fragmented mRNA was used as input, while the remaining RNA was incubated overnight with anti-m^6^A antibody (Synaptic Systems, Gottingen, Germany) or anti-IgG antibody in 500 μl of IP buffer at 4 °C. The following procedure was performed in the same manner as that described for the RIP assay.

### RNA pull-down assay

An RNA pull-down assay was performed using a Magnetic RNA-Protein Pull-down Kit (Thermo Fisher Scientific) according to the manufacturer’s instructions. The 3’-end Biotin-TEG modified-DNA probes against NLRP3 were synthesized by RiboBio. The biotinylated NLRP3 probe (50 pmol) was incubated with streptavidin beads to generate probe-coated beads. Lung tissue homogenates with probe-coated beads was incubated at 4 °C overnight. After three washes, proteins bound to the beads was boiled and used for the immunoblotting.

### Statistical analysis

All sample size information is shown (figure legends). Statistical analyses were conducted using GraphPad Prism 8.0 (GraphPad software Inc, San Diego, CA, USA). Quantitative data were assessed for normality test and presented as the means ± SD. Differences between two normally distributed groups were analyzed using a two-tailed unpaired Student’s *t* test. Multiple comparisons of parametric data were performed using one-way ANOVA. Exact *P* values are indicated in all figures, and statistical significance was set at *P* < 0.05.

### Supplementary information

Supplementary Table 1

Supplementary Table 2

Supplementary Table 3

Supplementary Fgure 1

Supplementary Fgure 2

Supplementary Figure Legends

Original western blots

## Data Availability

Data are available from the corresponding author on reasonable request.
