# Supplementary material for: METTL14 contributes to acute lung injury by stabilizing NLRP3 expression in an IGF2BP2-dependent manner
Source: Cell Death Dis. 2024 Jan 13;15(1):43. doi: 10.1038/s41419-023-06407-6 (PMC10787837; doi:10.1038/s41419-023-06407-6)
Supplement: Supplementary file 1 — Supplementary Table 1 [file 41419_2023_6407_MOESM1_ESM.docx]

**Table 1. Sequences of primers for qRT-PCR**

| **Gene name** | **Forward 5’-3’** | **Reverse 5’-3’** |
| --- | --- | --- |
| Nlrp3 | AAAGCTAAGAAGGACCAGCCAG | ATATCCCAGCAAACCCATCCAC |
| Il-1b | TGCCACCTTTTGACAGTGATGA | TGCCTGAAGCTCTTGTTGATGT |
| Il-18 | CGACCGAACAGCCAACGAAT | GGGTCACAGCCAGTCCTCTT |
| Mettl3 | CATCCGTCTTGCCATCTCTACG | AGGAGACCTCGCTTTACCTCAA |
| Mettl14 | GAAATGCTGGACCTGGGATGAT | TCTTCTGTAACCCCACTTTCGC |
| Mettl16 | GACATAGGTACTGGGGCATCCT | CATCCACTTCTGTTGCCAGGAA |
| Wtap | AGACAAACTGGAACAAGCCCAA | TCGACACTTCGCCATTAGCTTT |
| Fto | CTCTTCACCAGGGAGACTGCTA | GAGTGGAACTAAACCGAGGCTG |
| Alkbh5 | CAGTGGGTATGCTGCTGATGAA | ATCGCGGTGCATCTAATCTTGT |
| Igf2bp1 | GCGGCCAGTTCTTGGTCAA | TTGGGCACCGAATGTTCAATC |
| Igf2bp2 | TGGAAGCGCATATCAGAGTG | AGCAAAGAAGTGCCCGATAA |
| Igf2bp3 | TATATCGGAAACCTCAGCGAGA | GGACCGAGTGCTCAACTTCT |
| Gapdh | AGGTCGGTGTGAACGGATTTG | GGGGTCGTTGATGGCAACA |
