# Supplementary material for: METTL14 contributes to acute lung injury by stabilizing NLRP3 expression in an IGF2BP2-dependent manner
Source: Cell Death Dis. 2024 Jan 13;15(1):43. doi: 10.1038/s41419-023-06407-6 (PMC10787837; doi:10.1038/s41419-023-06407-6)
Supplement: Supplementary file 2 — Supplementary Table 2 [file 41419_2023_6407_MOESM2_ESM.docx]

**Table 2. Sequences of siRNA for gene knockdown**

| **siRNA** | **Sequence** |
| --- | --- |
| si-Mettl14 01 | GCATTGGTGCTGTGTTAAA |
| si-Mettl14 02 | GCAGCACCTCGGTCATTTA |
| si-Mettl14 03 | CCGGATGTACAGAGGAAAT |
| si-Igf2bp2 01 | GAATCCAGATTCGGAACAT |
| si-Igf2bp2 02 | CGGTTACTCAAGCGAACAA |
| si-Igf2bp2 03 | GCAATTTATACGAGGTTGT |
