# Supplementary material for: METTL14 contributes to acute lung injury by stabilizing NLRP3 expression in an IGF2BP2-dependent manner
Source: Cell Death Dis. 2024 Jan 13;15(1):43. doi: 10.1038/s41419-023-06407-6 (PMC10787837; doi:10.1038/s41419-023-06407-6)
Supplement: Supplementary file 3 — Supplementary Table 3 [file 41419_2023_6407_MOESM3_ESM.docx]

**Table 3. The predicted 24 m^6^A residues located across Nlrp3 sequence according to online SRAMP database.**

| Target | Position | Sequence context | Score  (binary) | Score  (knn) | Score  (spectrum) | Score  (combined) | Decision |
| --- | --- | --- | --- | --- | --- | --- | --- |
| 1 | 40 | CCCAAGGCUGCUAUCUGGAGGAACUUUUCUUCCAUGGCUCAGGAC | 0.665 | 0.571 | 0.435 | 0.568 | m6A site (Low confidence) |
| 2 | 87 | ACGUCUGGAUCAAGCUAAGAGAACUUUCUGUGUGGACCUAAGCCC | 0.728 | 0.561 | 0.448 | 0.607 | m6A site (Moderate confidence) |
| 3 | 311 | GAUCUAGCCACACUCAUGAUUGACUUCAAUGGCGAGGAGAAGGCC | 0.566 | 0.791 | 0.62 | 0.599 | m6A site (Moderate confidence) |
| 4 | 584 | AGCAGGUUCUACUCUAUCAAGGACAGGAACGCGCGUCUAGGUGAG | 0.646 | 0.507 | 0.482 | 0.573 | m6A site (Low confidence) |
| 5 | 703 | UGAACUCCUGACCAUCGGCCGGACUAAAAUGCGGGACAGCCCCAU | 0.684 | 0.379 | 0.405 | 0.557 | m6A site (Low confidence) |
| 6 | 851 | CUAGCCAGGAAGAUUAUGUUGGACUGGGCACUGGGAAAGCUCUUC | 0.76 | 0.741 | 0.533 | 0.668 | m6A site (High confidence) |
| 7 | 887 | AAGCUCUUCAAAGACAAAUUUGACUAUUUGUUCUUUAUCCACUGC | 0.496 | 0.584 | 0.65 | 0.562 | m6A site (Low confidence) |
| 8 | 1088 | CACAUUGGGGAGGUCUGCACAGACUGGCAAAAGGCUGUGCGGGGA | 0.688 | 0.399 | 0.644 | 0.656 | m6A site (High confidence) |
| 9 | 1664 | AACGUGUUCCAGAAGGAAGUGGACUGCGAGAGAUUCUACAGCUUC | 0.652 | 0.572 | 0.443 | 0.564 | m6A site (Low confidence) |
| 10 | 1947 | GCAAGAUCUCUCAGCAAGUCAGACUGGAACUACUGAAGUGGAUUG | 0.616 | 0.469 | 0.498 | 0.561 | m6A site (Low confidence) |
| 11 | 2019 | AGUGGCAGCCCAGCCAACUGGAACUGUUCUACUGCCUGUACGAGA | 0.695 | 0.649 | 0.561 | 0.639 | m6A site (High confidence) |
| 12 | 2054 | CUGUACGAGAUGCAGGAGGAAGACUUUGUGCAGAGUGCCAUGGAC | 0.651 | 0.415 | 0.628 | 0.63 | m6A site (High confidence) |
| 13 | 2117 | AUCAACCUCUCUACCAGAAUGGACCACGUGGUUUCCUCCUUUUGU | 0.665 | 0.343 | 0.575 | 0.613 | m6A site (Moderate confidence) |
| 14 | 2147 | GUUUCCUCCUUUUGUAUUAAGAACUGUCAUAGGGUCAAAACGCUU | 0.72 | 0.607 | 0.481 | 0.618 | m6A site (Moderate confidence) |
| 15 | 2364 | GCACCAACCGGAGCCUCACUGAACUGGACCUCAGUGACAAUACUC | 0.684 | 0.579 | 0.531 | 0.617 | m6A site (Moderate confidence) |
| 16 | 2451 | ACCCAGGCUGUAACAUUCAGAGACUGUGGUUGGGGCGCUGCGGAC | 0.751 | 0.567 | 0.453 | 0.623 | m6A site (High confidence) |
| 17 | 2472 | GACUGUGGUUGGGGCGCUGCGGACUGUCCCAUCAAUGCUGCUUCG | 0.697 | 0.58 | 0.448 | 0.591 | m6A site (Moderate confidence) |
| 18 | 2564 | CUCAGUGACAAUGCCCUGGGGGACUUUGGAAUCAGAUUGCUGUGU | 0.752 | 0.663 | 0.543 | 0.664 | m6A site (High confidence) |
| 19 | 2592 | GAAUCAGAUUGCUGUGUGUGGGACUGAAGCACCUGCUCUGCAACC | 0.727 | 0.694 | 0.527 | 0.645 | m6A site (High confidence) |
| 20 | 2706 | GCUCCAACCAUUCUCUGACCAGACUGUACAUUGGAGAAAAUGCCU | 0.668 | 0.671 | 0.489 | 0.596 | m6A site (Moderate confidence) |
| 21 | 2735 | AUUGGAGAAAAUGCCUUGGGAGACUCAGGAGUCCAAGUUUUGUGU | 0.518 | 0.495 | 0.662 | 0.574 | m6A site (Low confidence) |
| 22 | 2867 | UCUGUGCUCAAAACCAACCAGAACUUCACACACCUCUAUCUACGA | 0.605 | 0.626 | 0.614 | 0.61 | m6A site (Moderate confidence) |
| 23 | 2913 | GCAAUGCCCUUGGAGACACAGGACUCAGGCUCCUCUGUGAGGGGC | 0.699 | 0.774 | 0.677 | 0.694 | m6A site (Very high confidence) |
| 24 | 2948 | UGUGAGGGGCUUCUGCACCCGGACUGUAAACUACAGAUGCUGGAA | 0.718 | 0.755 | 0.65 | 0.693 | m6A site (Very high confidence) |
