# Supplementary figures and images for: METTL14 contributes to acute lung injury by stabilizing NLRP3 expression in an IGF2BP2-dependent manner
Source: Cell Death Dis. 2024 Jan 13;15(1):43. doi: 10.1038/s41419-023-06407-6 (PMC10787837; doi:10.1038/s41419-023-06407-6)

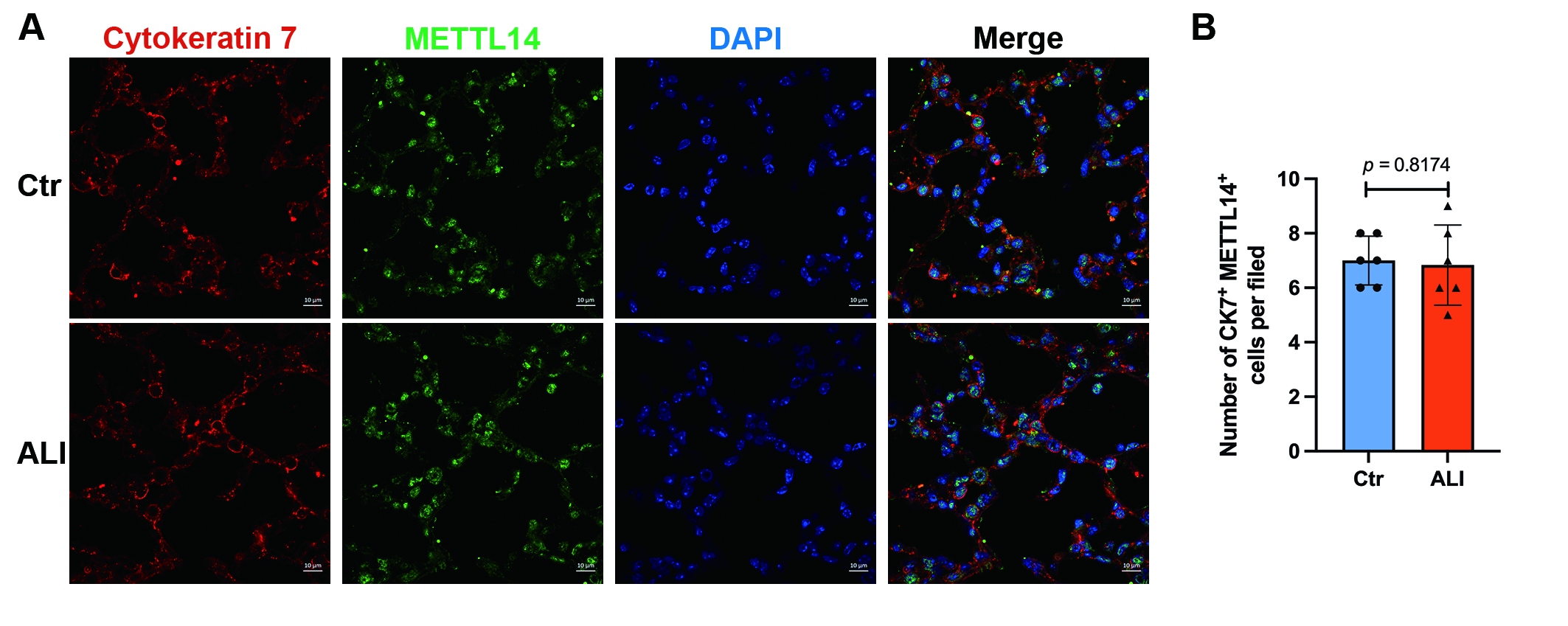

Supplement: Supplementary file 4 — Supplementary Fgure 1 [file 41419_2023_6407_MOESM4_ESM.tif]

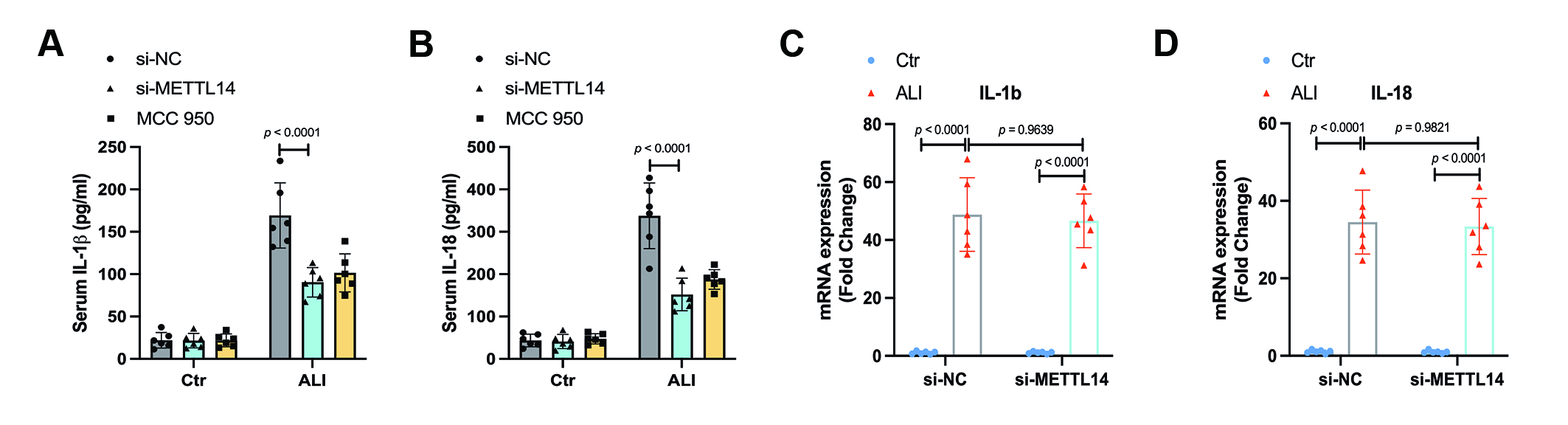

Supplement: Supplementary file 5 — Supplementary Fgure 2 [file 41419_2023_6407_MOESM5_ESM.tif]
