# Supplementary material for: METTL14 contributes to acute lung injury by stabilizing NLRP3 expression in an IGF2BP2-dependent manner
Source: Cell Death Dis. 2024 Jan 13;15(1):43. doi: 10.1038/s41419-023-06407-6 (PMC10787837; doi:10.1038/s41419-023-06407-6)
Supplement: Supplementary file 6 — Supplementary Figure Legends [file 41419_2023_6407_MOESM6_ESM.docx]

**Supplementary Fig.1 Expression of METTL14 in lung epithelial cells following ALI.** (A) Double-immunostaining and (B) quantification of METTL14 (green)/Cytokeratin 7 (CK7, a marker for lung epithelial cells, red) positive cells in mouse lungs from Ctr and ALI mice. Bars = 10μm. The graphs depict mean ± SD, n = 6 mice per group.

**Supplementary Fig.2 Effects of METTL14 knockdown on NLRP3-associated cytokines in ALI model.** (A) The IL-1β and (B) IL-18 concentrations in serum of Ctr and ALI mice following METTL14 knockdown or MCC950 treatment are shown (n = 6). (C, D) RT-qPCR analysis of IL-1b and IL-18 mRNA expression in lung tissue of Ctr and ALI mice treated with si-NC or si-METTL14 (n = 6). All data are represented as mean ± SD, n = 6 mice per group.

1. Jiao Y, Zhang T, Zhang C, Ji H, Tong X, Xia R, et al. Exosomal miR-30d-5p of neutrophils induces M1 macrophage polarization and primes macrophage pyroptosis in sepsis-related acute lung injury. Crit Care 2021, 25(1): 356.

1. Meyer NJ, Gattinoni L, Calfee CS. Acute respiratory distress syndrome. Lancet 2021, 398(10300): 622-637.
2. Jiao Y, Zhang T, Zhang C, Ji H, Tong X, Xia R, et al. Exosomal miR-30d-5p of neutrophils induces M1 macrophage polarization and primes macrophage pyroptosis in sepsis-related acute lung injury. Crit Care 2021, 25(1): 356.

1. Meyer NJ, Gattinoni L, Calfee CS. Acute respiratory distress syndrome. Lancet 2021, 398(10300): 622-637.

2. Gorman EA, O'Kane CM, McAuley DF. Acute respiratory distress syndrome in adults: diagnosis, outcomes, long-term sequelae, and management. Lancet 2022, 400(10358): 1157-1170.

3. Jiao Y, Zhang T, Zhang C, Ji H, Tong X, Xia R, et al. Exosomal miR-30d-5p of neutrophils induces M1 macrophage polarization and primes macrophage pyroptosis in sepsis-related acute lung injury. Crit Care 2021, 25(1): 356.

1. Meyer NJ, Gattinoni L, Calfee CS. Acute respiratory distress syndrome. Lancet 2021, 398(10300): 622-637.

2. Gorman EA, O'Kane CM, McAuley DF. Acute respiratory distress syndrome in adults: diagnosis, outcomes, long-term sequelae, and management. Lancet 2022, 400(10358): 1157-1170.

3. Bos LDJ, Ware LB. Acute respiratory distress syndrome: causes, pathophysiology, and phenotypes. Lancet 2022, 400(10358): 1145-1156.

4. Jiao Y, Zhang T, Zhang C, Ji H, Tong X, Xia R, et al. Exosomal miR-30d-5p of neutrophils induces M1 macrophage polarization and primes macrophage pyroptosis in sepsis-related acute lung injury. Crit Care 2021, 25(1): 356.

1. Meyer NJ, Gattinoni L, Calfee CS. Acute respiratory distress syndrome. Lancet 2021, 398(10300): 622-637.

2. Gorman EA, O'Kane CM, McAuley DF. Acute respiratory distress syndrome in adults: diagnosis, outcomes, long-term sequelae, and management. Lancet 2022, 400(10358): 1157-1170.

3. Bos LDJ, Ware LB. Acute respiratory distress syndrome: causes, pathophysiology, and phenotypes. Lancet 2022, 400(10358): 1145-1156.

4. Gilroy DW, De Maeyer RPH, Tepper M, O'Brien A, Uddin M, Chen J, et al. Treating exuberant, non-resolving inflammation in the lung; Implications for acute respiratory distress syndrome and COVID-19. Pharmacol Ther 2021, 221: 107745.

5. Jiao Y, Zhang T, Zhang C, Ji H, Tong X, Xia R, et al. Exosomal miR-30d-5p of neutrophils induces M1 macrophage polarization and primes macrophage pyroptosis in sepsis-related acute lung injury. Crit Care 2021, 25(1): 356.

1. Meyer NJ, Gattinoni L, Calfee CS. Acute respiratory distress syndrome. Lancet 2021, 398(10300): 622-637.

2. Gorman EA, O'Kane CM, McAuley DF. Acute respiratory distress syndrome in adults: diagnosis, outcomes, long-term sequelae, and management. Lancet 2022, 400(10358): 1157-1170.

3. Bos LDJ, Ware LB. Acute respiratory distress syndrome: causes, pathophysiology, and phenotypes. Lancet 2022, 400(10358): 1145-1156.

4. Gilroy DW, De Maeyer RPH, Tepper M, O'Brien A, Uddin M, Chen J, et al. Treating exuberant, non-resolving inflammation in the lung; Implications for acute respiratory distress syndrome and COVID-19. Pharmacol Ther 2021, 221: 107745.

5. Laffey JG, Matthay MA. Fifty Years of Research in ARDS. Cell-based Therapy for Acute Respiratory Distress Syndrome. Biology and Potential Therapeutic Value. Am J Respir Crit Care Med 2017, 196(3): 266-273.

6. Jiao Y, Zhang T, Zhang C, Ji H, Tong X, Xia R, et al. Exosomal miR-30d-5p of neutrophils induces M1 macrophage polarization and primes macrophage pyroptosis in sepsis-related acute lung injury. Crit Care 2021, 25(1): 356.

1. Meyer NJ, Gattinoni L, Calfee CS. Acute respiratory distress syndrome. Lancet 2021, 398(10300): 622-637.

2. Gorman EA, O'Kane CM, McAuley DF. Acute respiratory distress syndrome in adults: diagnosis, outcomes, long-term sequelae, and management. Lancet 2022, 400(10358): 1157-1170.

3. Bos LDJ, Ware LB. Acute respiratory distress syndrome: causes, pathophysiology, and phenotypes. Lancet 2022, 400(10358): 1145-1156.

4. Gilroy DW, De Maeyer RPH, Tepper M, O'Brien A, Uddin M, Chen J, et al. Treating exuberant, non-resolving inflammation in the lung; Implications for acute respiratory distress syndrome and COVID-19. Pharmacol Ther 2021, 221: 107745.

5. Laffey JG, Matthay MA. Fifty Years of Research in ARDS. Cell-based Therapy for Acute Respiratory Distress Syndrome. Biology and Potential Therapeutic Value. Am J Respir Crit Care Med 2017, 196(3): 266-273.

6. Xian H, Liu Y, Rundberg Nilsson A, Gatchalian R, Crother TR, Tourtellotte WG, et al. Metformin inhibition of mitochondrial ATP and DNA synthesis abrogates NLRP3 inflammasome activation and pulmonary inflammation. Immunity 2021, 54(7): 1463-1477 e1411.

7. Jiao Y, Zhang T, Zhang C, Ji H, Tong X, Xia R, et al. Exosomal miR-30d-5p of neutrophils induces M1 macrophage polarization and primes macrophage pyroptosis in sepsis-related acute lung injury. Crit Care 2021, 25(1): 356.

1. Meyer NJ, Gattinoni L, Calfee CS. Acute respiratory distress syndrome. Lancet 2021, 398(10300): 622-637.

2. Gorman EA, O'Kane CM, McAuley DF. Acute respiratory distress syndrome in adults: diagnosis, outcomes, long-term sequelae, and management. Lancet 2022, 400(10358): 1157-1170.

3. Bos LDJ, Ware LB. Acute respiratory distress syndrome: causes, pathophysiology, and phenotypes. Lancet 2022, 400(10358): 1145-1156.

4. Gilroy DW, De Maeyer RPH, Tepper M, O'Brien A, Uddin M, Chen J, et al. Treating exuberant, non-resolving inflammation in the lung; Implications for acute respiratory distress syndrome and COVID-19. Pharmacol Ther 2021, 221: 107745.

5. Laffey JG, Matthay MA. Fifty Years of Research in ARDS. Cell-based Therapy for Acute Respiratory Distress Syndrome. Biology and Potential Therapeutic Value. Am J Respir Crit Care Med 2017, 196(3): 266-273.

6. Xian H, Liu Y, Rundberg Nilsson A, Gatchalian R, Crother TR, Tourtellotte WG, et al. Metformin inhibition of mitochondrial ATP and DNA synthesis abrogates NLRP3 inflammasome activation and pulmonary inflammation. Immunity 2021, 54(7): 1463-1477 e1411.

7. Paik S, Kim JK, Silwal P, Sasakawa C, Jo EK. An update on the regulatory mechanisms of NLRP3 inflammasome activation. Cell Mol Immunol 2021, 18(5): 1141-1160.

8. Jiao Y, Zhang T, Zhang C, Ji H, Tong X, Xia R, et al. Exosomal miR-30d-5p of neutrophils induces M1 macrophage polarization and primes macrophage pyroptosis in sepsis-related acute lung injury. Crit Care 2021, 25(1): 356.

1. Meyer NJ, Gattinoni L, Calfee CS. Acute respiratory distress syndrome. Lancet 2021, 398(10300): 622-637.

2. Gorman EA, O'Kane CM, McAuley DF. Acute respiratory distress syndrome in adults: diagnosis, outcomes, long-term sequelae, and management. Lancet 2022, 400(10358): 1157-1170.

3. Bos LDJ, Ware LB. Acute respiratory distress syndrome: causes, pathophysiology, and phenotypes. Lancet 2022, 400(10358): 1145-1156.

4. Gilroy DW, De Maeyer RPH, Tepper M, O'Brien A, Uddin M, Chen J, et al. Treating exuberant, non-resolving inflammation in the lung; Implications for acute respiratory distress syndrome and COVID-19. Pharmacol Ther 2021, 221: 107745.

5. Laffey JG, Matthay MA. Fifty Years of Research in ARDS. Cell-based Therapy for Acute Respiratory Distress Syndrome. Biology and Potential Therapeutic Value. Am J Respir Crit Care Med 2017, 196(3): 266-273.

6. Xian H, Liu Y, Rundberg Nilsson A, Gatchalian R, Crother TR, Tourtellotte WG, et al. Metformin inhibition of mitochondrial ATP and DNA synthesis abrogates NLRP3 inflammasome activation and pulmonary inflammation. Immunity 2021, 54(7): 1463-1477 e1411.

7. Paik S, Kim JK, Silwal P, Sasakawa C, Jo EK. An update on the regulatory mechanisms of NLRP3 inflammasome activation. Cell Mol Immunol 2021, 18(5): 1141-1160.

8. Kelley N, Jeltema D, Duan Y, He Y. The NLRP3 Inflammasome: An Overview of Mechanisms of Activation and Regulation. Int J Mol Sci 2019, 20(13).

9. Jiao Y, Zhang T, Zhang C, Ji H, Tong X, Xia R, et al. Exosomal miR-30d-5p of neutrophils induces M1 macrophage polarization and primes macrophage pyroptosis in sepsis-related acute lung injury. Crit Care 2021, 25(1): 356.

1. Meyer NJ, Gattinoni L, Calfee CS. Acute respiratory distress syndrome. Lancet 2021, 398(10300): 622-637.

2. Gorman EA, O'Kane CM, McAuley DF. Acute respiratory distress syndrome in adults: diagnosis, outcomes, long-term sequelae, and management. Lancet 2022, 400(10358): 1157-1170.

3. Bos LDJ, Ware LB. Acute respiratory distress syndrome: causes, pathophysiology, and phenotypes. Lancet 2022, 400(10358): 1145-1156.

4. Gilroy DW, De Maeyer RPH, Tepper M, O'Brien A, Uddin M, Chen J, et al. Treating exuberant, non-resolving inflammation in the lung; Implications for acute respiratory distress syndrome and COVID-19. Pharmacol Ther 2021, 221: 107745.

5. Laffey JG, Matthay MA. Fifty Years of Research in ARDS. Cell-based Therapy for Acute Respiratory Distress Syndrome. Biology and Potential Therapeutic Value. Am J Respir Crit Care Med 2017, 196(3): 266-273.

6. Xian H, Liu Y, Rundberg Nilsson A, Gatchalian R, Crother TR, Tourtellotte WG, et al. Metformin inhibition of mitochondrial ATP and DNA synthesis abrogates NLRP3 inflammasome activation and pulmonary inflammation. Immunity 2021, 54(7): 1463-1477 e1411.

7. Paik S, Kim JK, Silwal P, Sasakawa C, Jo EK. An update on the regulatory mechanisms of NLRP3 inflammasome activation. Cell Mol Immunol 2021, 18(5): 1141-1160.

8. Kelley N, Jeltema D, Duan Y, He Y. The NLRP3 Inflammasome: An Overview of Mechanisms of Activation and Regulation. Int J Mol Sci 2019, 20(13).

9. Swanson KV, Deng M, Ting JP. The NLRP3 inflammasome: molecular activation and regulation to therapeutics. Nat Rev Immunol 2019, 19(8): 477-489.

10. Jiao Y, Zhang T, Zhang C, Ji H, Tong X, Xia R, et al. Exosomal miR-30d-5p of neutrophils induces M1 macrophage polarization and primes macrophage pyroptosis in sepsis-related acute lung injury. Crit Care 2021, 25(1): 356.

1. Meyer NJ, Gattinoni L, Calfee CS. Acute respiratory distress syndrome. Lancet 2021, 398(10300): 622-637.

2. Gorman EA, O'Kane CM, McAuley DF. Acute respiratory distress syndrome in adults: diagnosis, outcomes, long-term sequelae, and management. Lancet 2022, 400(10358): 1157-1170.

3. Bos LDJ, Ware LB. Acute respiratory distress syndrome: causes, pathophysiology, and phenotypes. Lancet 2022, 400(10358): 1145-1156.

4. Gilroy DW, De Maeyer RPH, Tepper M, O'Brien A, Uddin M, Chen J, et al. Treating exuberant, non-resolving inflammation in the lung; Implications for acute respiratory distress syndrome and COVID-19. Pharmacol Ther 2021, 221: 107745.

5. Laffey JG, Matthay MA. Fifty Years of Research in ARDS. Cell-based Therapy for Acute Respiratory Distress Syndrome. Biology and Potential Therapeutic Value. Am J Respir Crit Care Med 2017, 196(3): 266-273.

6. Xian H, Liu Y, Rundberg Nilsson A, Gatchalian R, Crother TR, Tourtellotte WG, et al. Metformin inhibition of mitochondrial ATP and DNA synthesis abrogates NLRP3 inflammasome activation and pulmonary inflammation. Immunity 2021, 54(7): 1463-1477 e1411.

7. Paik S, Kim JK, Silwal P, Sasakawa C, Jo EK. An update on the regulatory mechanisms of NLRP3 inflammasome activation. Cell Mol Immunol 2021, 18(5): 1141-1160.

8. Kelley N, Jeltema D, Duan Y, He Y. The NLRP3 Inflammasome: An Overview of Mechanisms of Activation and Regulation. Int J Mol Sci 2019, 20(13).

9. Swanson KV, Deng M, Ting JP. The NLRP3 inflammasome: molecular activation and regulation to therapeutics. Nat Rev Immunol 2019, 19(8): 477-489.

10. Bauernfeind FG, Horvath G, Stutz A, Alnemri ES, MacDonald K, Speert D, et al. Cutting edge: NF-kappaB activating pattern recognition and cytokine receptors license NLRP3 inflammasome activation by regulating NLRP3 expression. J Immunol 2009, 183(2): 787-791.

11. Jiao Y, Zhang T, Zhang C, Ji H, Tong X, Xia R, et al. Exosomal miR-30d-5p of neutrophils induces M1 macrophage polarization and primes macrophage pyroptosis in sepsis-related acute lung injury. Crit Care 2021, 25(1): 356.

1. Meyer NJ, Gattinoni L, Calfee CS. Acute respiratory distress syndrome. Lancet 2021, 398(10300): 622-637.

2. Gorman EA, O'Kane CM, McAuley DF. Acute respiratory distress syndrome in adults: diagnosis, outcomes, long-term sequelae, and management. Lancet 2022, 400(10358): 1157-1170.

3. Bos LDJ, Ware LB. Acute respiratory distress syndrome: causes, pathophysiology, and phenotypes. Lancet 2022, 400(10358): 1145-1156.

4. Gilroy DW, De Maeyer RPH, Tepper M, O'Brien A, Uddin M, Chen J, et al. Treating exuberant, non-resolving inflammation in the lung; Implications for acute respiratory distress syndrome and COVID-19. Pharmacol Ther 2021, 221: 107745.

5. Laffey JG, Matthay MA. Fifty Years of Research in ARDS. Cell-based Therapy for Acute Respiratory Distress Syndrome. Biology and Potential Therapeutic Value. Am J Respir Crit Care Med 2017, 196(3): 266-273.

7. Paik S, Kim JK, Silwal P, Sasakawa C, Jo EK. An update on the regulatory mechanisms of NLRP3 inflammasome activation. Cell Mol Immunol 2021, 18(5): 1141-1160.

8. Kelley N, Jeltema D, Duan Y, He Y. The NLRP3 Inflammasome: An Overview of Mechanisms of Activation and Regulation. Int J Mol Sci 2019, 20(13).

9. Swanson KV, Deng M, Ting JP. The NLRP3 inflammasome: molecular activation and regulation to therapeutics. Nat Rev Immunol 2019, 19(8): 477-489.

10. Bauernfeind FG, Horvath G, Stutz A, Alnemri ES, MacDonald K, Speert D, et al. Cutting edge: NF-kappaB activating pattern recognition and cytokine receptors license NLRP3 inflammasome activation by regulating NLRP3 expression. J Immunol 2009, 183(2): 787-791.

11. Cornut M, Bourdonnay E, Henry T. Transcriptional Regulation of Inflammasomes. Int J Mol Sci 2020, 21(21).

12. Jiao Y, Zhang T, Zhang C, Ji H, Tong X, Xia R, et al. Exosomal miR-30d-5p of neutrophils induces M1 macrophage polarization and primes macrophage pyroptosis in sepsis-related acute lung injury. Crit Care 2021, 25(1): 356.

1. Meyer NJ, Gattinoni L, Calfee CS. Acute respiratory distress syndrome. Lancet 2021, 398(10300): 622-637.

2. Gorman EA, O'Kane CM, McAuley DF. Acute respiratory distress syndrome in adults: diagnosis, outcomes, long-term sequelae, and management. Lancet 2022, 400(10358): 1157-1170.

3. Bos LDJ, Ware LB. Acute respiratory distress syndrome: causes, pathophysiology, and phenotypes. Lancet 2022, 400(10358): 1145-1156.

4. Gilroy DW, De Maeyer RPH, Tepper M, O'Brien A, Uddin M, Chen J, et al. Treating exuberant, non-resolving inflammation in the lung; Implications for acute respiratory distress syndrome and COVID-19. Pharmacol Ther 2021, 221: 107745.

5. Laffey JG, Matthay MA. Fifty Years of Research in ARDS. Cell-based Therapy for Acute Respiratory Distress Syndrome. Biology and Potential Therapeutic Value. Am J Respir Crit Care Med 2017, 196(3): 266-273.

6. Xian H, Liu Y, Rundberg Nilsson A, Gatchalian R, Crother TR, Tourtellotte WG, et al. Metformin inhibition of mitochondrial ATP and DNA synthesis abrogates NLRP3 inflammasome activation and pulmonary inflammation. Immunity 2021, 54(7): 1463-1477 e1411.

7. Paik S, Kim JK, Silwal P, Sasakawa C, Jo EK. An update on the regulatory mechanisms of NLRP3 inflammasome activation. Cell Mol Immunol 2021, 18(5): 1141-1160.

8. Kelley N, Jeltema D, Duan Y, He Y. The NLRP3 Inflammasome: An Overview of Mechanisms of Activation and Regulation. Int J Mol Sci 2019, 20(13).

9. Swanson KV, Deng M, Ting JP. The NLRP3 inflammasome: molecular activation and regulation to therapeutics. Nat Rev Immunol 2019, 19(8): 477-489.

10. Bauernfeind FG, Horvath G, Stutz A, Alnemri ES, MacDonald K, Speert D, et al. Cutting edge: NF-kappaB activating pattern recognition and cytokine receptors license NLRP3 inflammasome activation by regulating NLRP3 expression. J Immunol 2009, 183(2): 787-791.

11. Cornut M, Bourdonnay E, Henry T. Transcriptional Regulation of Inflammasomes. Int J Mol Sci 2020, 21(21).

12. Baker PJ, De Nardo D, Moghaddas F, Tran LS, Bachem A, Nguyen T, et al. Posttranslational Modification as a Critical Determinant of Cytoplasmic Innate Immune Recognition. Physiol Rev 2017, 97(3): 1165-1209.

13. Jiao Y, Zhang T, Zhang C, Ji H, Tong X, Xia R, et al. Exosomal miR-30d-5p of neutrophils induces M1 macrophage polarization and primes macrophage pyroptosis in sepsis-related acute lung injury. Crit Care 2021, 25(1): 356.

1. Meyer NJ, Gattinoni L, Calfee CS. Acute respiratory distress syndrome. Lancet 2021, 398(10300): 622-637.

2. Gorman EA, O'Kane CM, McAuley DF. Acute respiratory distress syndrome in adults: diagnosis, outcomes, long-term sequelae, and management. Lancet 2022, 400(10358): 1157-1170.

3. Bos LDJ, Ware LB. Acute respiratory distress syndrome: causes, pathophysiology, and phenotypes. Lancet 2022, 400(10358): 1145-1156.

4. Gilroy DW, De Maeyer RPH, Tepper M, O'Brien A, Uddin M, Chen J, et al. Treating exuberant, non-resolving inflammation in the lung; Implications for acute respiratory distress syndrome and COVID-19. Pharmacol Ther 2021, 221: 107745.

5. Laffey JG, Matthay MA. Fifty Years of Research in ARDS. Cell-based Therapy for Acute Respiratory Distress Syndrome. Biology and Potential Therapeutic Value. Am J Respir Crit Care Med 2017, 196(3): 266-273.

6. Xian H, Liu Y, Rundberg Nilsson A, Gatchalian R, Crother TR, Tourtellotte WG, et al. Metformin inhibition of mitochondrial ATP and DNA synthesis abrogates NLRP3 inflammasome activation and pulmonary inflammation. Immunity 2021, 54(7): 1463-1477 e1411.

7. Paik S, Kim JK, Silwal P, Sasakawa C, Jo EK. An update on the regulatory mechanisms of NLRP3 inflammasome activation. Cell Mol Immunol 2021, 18(5): 1141-1160.

8. Kelley N, Jeltema D, Duan Y, He Y. The NLRP3 Inflammasome: An Overview of Mechanisms of Activation and Regulation. Int J Mol Sci 2019, 20(13).

9. Swanson KV, Deng M, Ting JP. The NLRP3 inflammasome: molecular activation and regulation to therapeutics. Nat Rev Immunol 2019, 19(8): 477-489.

10. Bauernfeind FG, Horvath G, Stutz A, Alnemri ES, MacDonald K, Speert D, et al. Cutting edge: NF-kappaB activating pattern recognition and cytokine receptors license NLRP3 inflammasome activation by regulating NLRP3 expression. J Immunol 2009, 183(2): 787-791.

11. Cornut M, Bourdonnay E, Henry T. Transcriptional Regulation of Inflammasomes. Int J Mol Sci 2020, 21(21).

13. Moretti J, Blander JM. Increasing complexity of NLRP3 inflammasome regulation. J Leukoc Biol 2021, 109(3): 561-571.

14. Jiao Y, Zhang T, Zhang C, Ji H, Tong X, Xia R, et al. Exosomal miR-30d-5p of neutrophils induces M1 macrophage polarization and primes macrophage pyroptosis in sepsis-related acute lung injury. Crit Care 2021, 25(1): 356.

1. Meyer NJ, Gattinoni L, Calfee CS. Acute respiratory distress syndrome. Lancet 2021, 398(10300): 622-637.

2. Gorman EA, O'Kane CM, McAuley DF. Acute respiratory distress syndrome in adults: diagnosis, outcomes, long-term sequelae, and management. Lancet 2022, 400(10358): 1157-1170.

3. Bos LDJ, Ware LB. Acute respiratory distress syndrome: causes, pathophysiology, and phenotypes. Lancet 2022, 400(10358): 1145-1156.

4. Gilroy DW, De Maeyer RPH, Tepper M, O'Brien A, Uddin M, Chen J, et al. Treating exuberant, non-resolving inflammation in the lung; Implications for acute respiratory distress syndrome and COVID-19. Pharmacol Ther 2021, 221: 107745.

5. Laffey JG, Matthay MA. Fifty Years of Research in ARDS. Cell-based Therapy for Acute Respiratory Distress Syndrome. Biology and Potential Therapeutic Value. Am J Respir Crit Care Med 2017, 196(3): 266-273.

6. Xian H, Liu Y, Rundberg Nilsson A, Gatchalian R, Crother TR, Tourtellotte WG, et al. Metformin inhibition of mitochondrial ATP and DNA synthesis abrogates NLRP3 inflammasome activation and pulmonary inflammation. Immunity 2021, 54(7): 1463-1477 e1411.

7. Paik S, Kim JK, Silwal P, Sasakawa C, Jo EK. An update on the regulatory mechanisms of NLRP3 inflammasome activation. Cell Mol Immunol 2021, 18(5): 1141-1160.

8. Kelley N, Jeltema D, Duan Y, He Y. The NLRP3 Inflammasome: An Overview of Mechanisms of Activation and Regulation. Int J Mol Sci 2019, 20(13).

9. Swanson KV, Deng M, Ting JP. The NLRP3 inflammasome: molecular activation and regulation to therapeutics. Nat Rev Immunol 2019, 19(8): 477-489.

10. Bauernfeind FG, Horvath G, Stutz A, Alnemri ES, MacDonald K, Speert D, et al. Cutting edge: NF-kappaB activating pattern recognition and cytokine receptors license NLRP3 inflammasome activation by regulating NLRP3 expression. J Immunol 2009, 183(2): 787-791.

11. Cornut M, Bourdonnay E, Henry T. Transcriptional Regulation of Inflammasomes. Int J Mol Sci 2020, 21(21).

12. Baker PJ, De Nardo D, Moghaddas F, Tran LS, Bachem A, Nguyen T, et al. Posttranslational Modification as a Critical Determinant of Cytoplasmic Innate Immune Recognition. Physiol Rev 2017, 97(3): 1165-1209.

13. Moretti J, Blander JM. Increasing complexity of NLRP3 inflammasome regulation. J Leukoc Biol 2021, 109(3): 561-571.

14. Liu Y, Yang D, Liu T, Chen J, Yu J, Yi P. N6-methyladenosine-mediated gene regulation and therapeutic implications. Trends Mol Med 2023, 29(6): 454-467.

15. Jiao Y, Zhang T, Zhang C, Ji H, Tong X, Xia R, et al. Exosomal miR-30d-5p of neutrophils induces M1 macrophage polarization and primes macrophage pyroptosis in sepsis-related acute lung injury. Crit Care 2021, 25(1): 356.

1. Meyer NJ, Gattinoni L, Calfee CS. Acute respiratory distress syndrome. Lancet 2021, 398(10300): 622-637.

2. Gorman EA, O'Kane CM, McAuley DF. Acute respiratory distress syndrome in adults: diagnosis, outcomes, long-term sequelae, and management. Lancet 2022, 400(10358): 1157-1170.

3. Bos LDJ, Ware LB. Acute respiratory distress syndrome: causes, pathophysiology, and phenotypes. Lancet 2022, 400(10358): 1145-1156.

4. Gilroy DW, De Maeyer RPH, Tepper M, O'Brien A, Uddin M, Chen J, et al. Treating exuberant, non-resolving inflammation in the lung; Implications for acute respiratory distress syndrome and COVID-19. Pharmacol Ther 2021, 221: 107745.

5. Laffey JG, Matthay MA. Fifty Years of Research in ARDS. Cell-based Therapy for Acute Respiratory Distress Syndrome. Biology and Potential Therapeutic Value. Am J Respir Crit Care Med 2017, 196(3): 266-273.

6. Xian H, Liu Y, Rundberg Nilsson A, Gatchalian R, Crother TR, Tourtellotte WG, et al. Metformin inhibition of mitochondrial ATP and DNA synthesis abrogates NLRP3 inflammasome activation and pulmonary inflammation. Immunity 2021, 54(7): 1463-1477 e1411.

7. Paik S, Kim JK, Silwal P, Sasakawa C, Jo EK. An update on the regulatory mechanisms of NLRP3 inflammasome activation. Cell Mol Immunol 2021, 18(5): 1141-1160.

8. Kelley N, Jeltema D, Duan Y, He Y. The NLRP3 Inflammasome: An Overview of Mechanisms of Activation and Regulation. Int J Mol Sci 2019, 20(13).

9. Swanson KV, Deng M, Ting JP. The NLRP3 inflammasome: molecular activation and regulation to therapeutics. Nat Rev Immunol 2019, 19(8): 477-489.

10. Bauernfeind FG, Horvath G, Stutz A, Alnemri ES, MacDonald K, Speert D, et al. Cutting edge: NF-kappaB activating pattern recognition and cytokine receptors license NLRP3 inflammasome activation by regulating NLRP3 expression. J Immunol 2009, 183(2): 787-791.

11. Cornut M, Bourdonnay E, Henry T. Transcriptional Regulation of Inflammasomes. Int J Mol Sci 2020, 21(21).

13. Moretti J, Blander JM. Increasing complexity of NLRP3 inflammasome regulation. J Leukoc Biol 2021, 109(3): 561-571.

14. Liu Y, Yang D, Liu T, Chen J, Yu J, Yi P. N6-methyladenosine-mediated gene regulation and therapeutic implications. Trends Mol Med 2023, 29(6): 454-467.

16. Jiao Y, Zhang T, Zhang C, Ji H, Tong X, Xia R, et al. Exosomal miR-30d-5p of neutrophils induces M1 macrophage polarization and primes macrophage pyroptosis in sepsis-related acute lung injury. Crit Care 2021, 25(1): 356.

1. Meyer NJ, Gattinoni L, Calfee CS. Acute respiratory distress syndrome. Lancet 2021, 398(10300): 622-637.

2. Gorman EA, O'Kane CM, McAuley DF. Acute respiratory distress syndrome in adults: diagnosis, outcomes, long-term sequelae, and management. Lancet 2022, 400(10358): 1157-1170.

3. Bos LDJ, Ware LB. Acute respiratory distress syndrome: causes, pathophysiology, and phenotypes. Lancet 2022, 400(10358): 1145-1156.

4. Gilroy DW, De Maeyer RPH, Tepper M, O'Brien A, Uddin M, Chen J, et al. Treating exuberant, non-resolving inflammation in the lung; Implications for acute respiratory distress syndrome and COVID-19. Pharmacol Ther 2021, 221: 107745.

5. Laffey JG, Matthay MA. Fifty Years of Research in ARDS. Cell-based Therapy for Acute Respiratory Distress Syndrome. Biology and Potential Therapeutic Value. Am J Respir Crit Care Med 2017, 196(3): 266-273.

6. Xian H, Liu Y, Rundberg Nilsson A, Gatchalian R, Crother TR, Tourtellotte WG, et al. Metformin inhibition of mitochondrial ATP and DNA synthesis abrogates NLRP3 inflammasome activation and pulmonary inflammation. Immunity 2021, 54(7): 1463-1477 e1411.

7. Paik S, Kim JK, Silwal P, Sasakawa C, Jo EK. An update on the regulatory mechanisms of NLRP3 inflammasome activation. Cell Mol Immunol 2021, 18(5): 1141-1160.

9. Swanson KV, Deng M, Ting JP. The NLRP3 inflammasome: molecular activation and regulation to therapeutics. Nat Rev Immunol 2019, 19(8): 477-489.

10. Bauernfeind FG, Horvath G, Stutz A, Alnemri ES, MacDonald K, Speert D, et al. Cutting edge: NF-kappaB activating pattern recognition and cytokine receptors license NLRP3 inflammasome activation by regulating NLRP3 expression. J Immunol 2009, 183(2): 787-791.

11. Cornut M, Bourdonnay E, Henry T. Transcriptional Regulation of Inflammasomes. Int J Mol Sci 2020, 21(21).

12. Baker PJ, De Nardo D, Moghaddas F, Tran LS, Bachem A, Nguyen T, et al. Posttranslational Modification as a Critical Determinant of Cytoplasmic Innate Immune Recognition. Physiol Rev 2017, 97(3): 1165-1209.

13. Moretti J, Blander JM. Increasing complexity of NLRP3 inflammasome regulation. J Leukoc Biol 2021, 109(3): 561-571.

14. Liu Y, Yang D, Liu T, Chen J, Yu J, Yi P. N6-methyladenosine-mediated gene regulation and therapeutic implications. Trends Mol Med 2023, 29(6): 454-467.

15. Zaccara S, Ries RJ, Jaffrey SR. Reading, writing and erasing mRNA methylation. Nat Rev Mol Cell Biol 2019, 20(10): 608-624.

16. Zhao Y, Shi Y, Shen H, Xie W. m(6)A-binding proteins: the emerging crucial performers in epigenetics. J Hematol Oncol 2020, 13(1): 35.

17. Jiao Y, Zhang T, Zhang C, Ji H, Tong X, Xia R, et al. Exosomal miR-30d-5p of neutrophils induces M1 macrophage polarization and primes macrophage pyroptosis in sepsis-related acute lung injury. Crit Care 2021, 25(1): 356.

1. Meyer NJ, Gattinoni L, Calfee CS. Acute respiratory distress syndrome. Lancet 2021, 398(10300): 622-637.

2. Gorman EA, O'Kane CM, McAuley DF. Acute respiratory distress syndrome in adults: diagnosis, outcomes, long-term sequelae, and management. Lancet 2022, 400(10358): 1157-1170.

3. Bos LDJ, Ware LB. Acute respiratory distress syndrome: causes, pathophysiology, and phenotypes. Lancet 2022, 400(10358): 1145-1156.

4. Gilroy DW, De Maeyer RPH, Tepper M, O'Brien A, Uddin M, Chen J, et al. Treating exuberant, non-resolving inflammation in the lung; Implications for acute respiratory distress syndrome and COVID-19. Pharmacol Ther 2021, 221: 107745.

5. Laffey JG, Matthay MA. Fifty Years of Research in ARDS. Cell-based Therapy for Acute Respiratory Distress Syndrome. Biology and Potential Therapeutic Value. Am J Respir Crit Care Med 2017, 196(3): 266-273.

6. Xian H, Liu Y, Rundberg Nilsson A, Gatchalian R, Crother TR, Tourtellotte WG, et al. Metformin inhibition of mitochondrial ATP and DNA synthesis abrogates NLRP3 inflammasome activation and pulmonary inflammation. Immunity 2021, 54(7): 1463-1477 e1411.

7. Paik S, Kim JK, Silwal P, Sasakawa C, Jo EK. An update on the regulatory mechanisms of NLRP3 inflammasome activation. Cell Mol Immunol 2021, 18(5): 1141-1160.

8. Kelley N, Jeltema D, Duan Y, He Y. The NLRP3 Inflammasome: An Overview of Mechanisms of Activation and Regulation. Int J Mol Sci 2019, 20(13).

9. Swanson KV, Deng M, Ting JP. The NLRP3 inflammasome: molecular activation and regulation to therapeutics. Nat Rev Immunol 2019, 19(8): 477-489.

10. Bauernfeind FG, Horvath G, Stutz A, Alnemri ES, MacDonald K, Speert D, et al. Cutting edge: NF-kappaB activating pattern recognition and cytokine receptors license NLRP3 inflammasome activation by regulating NLRP3 expression. J Immunol 2009, 183(2): 787-791.

11. Cornut M, Bourdonnay E, Henry T. Transcriptional Regulation of Inflammasomes. Int J Mol Sci 2020, 21(21).

12. Baker PJ, De Nardo D, Moghaddas F, Tran LS, Bachem A, Nguyen T, et al. Posttranslational Modification as a Critical Determinant of Cytoplasmic Innate Immune Recognition. Physiol Rev 2017, 97(3): 1165-1209.

13. Moretti J, Blander JM. Increasing complexity of NLRP3 inflammasome regulation. J Leukoc Biol 2021, 109(3): 561-571.

14. Liu Y, Yang D, Liu T, Chen J, Yu J, Yi P. N6-methyladenosine-mediated gene regulation and therapeutic implications. Trends Mol Med 2023, 29(6): 454-467.

15. Zaccara S, Ries RJ, Jaffrey SR. Reading, writing and erasing mRNA methylation. Nat Rev Mol Cell Biol 2019, 20(10): 608-624.

17. Xu H, Lin C, Yang J, Chen X, Chen Y, Chen J, et al. The Role of N(6)-Methyladenosine in Inflammatory Diseases. Oxid Med Cell Longev 2022, 2022: 9744771.

18. Jiao Y, Zhang T, Zhang C, Ji H, Tong X, Xia R, et al. Exosomal miR-30d-5p of neutrophils induces M1 macrophage polarization and primes macrophage pyroptosis in sepsis-related acute lung injury. Crit Care 2021, 25(1): 356.

1. Meyer NJ, Gattinoni L, Calfee CS. Acute respiratory distress syndrome. Lancet 2021, 398(10300): 622-637.

2. Gorman EA, O'Kane CM, McAuley DF. Acute respiratory distress syndrome in adults: diagnosis, outcomes, long-term sequelae, and management. Lancet 2022, 400(10358): 1157-1170.

3. Bos LDJ, Ware LB. Acute respiratory distress syndrome: causes, pathophysiology, and phenotypes. Lancet 2022, 400(10358): 1145-1156.

4. Gilroy DW, De Maeyer RPH, Tepper M, O'Brien A, Uddin M, Chen J, et al. Treating exuberant, non-resolving inflammation in the lung; Implications for acute respiratory distress syndrome and COVID-19. Pharmacol Ther 2021, 221: 107745.

5. Laffey JG, Matthay MA. Fifty Years of Research in ARDS. Cell-based Therapy for Acute Respiratory Distress Syndrome. Biology and Potential Therapeutic Value. Am J Respir Crit Care Med 2017, 196(3): 266-273.

6. Xian H, Liu Y, Rundberg Nilsson A, Gatchalian R, Crother TR, Tourtellotte WG, et al. Metformin inhibition of mitochondrial ATP and DNA synthesis abrogates NLRP3 inflammasome activation and pulmonary inflammation. Immunity 2021, 54(7): 1463-1477 e1411.

7. Paik S, Kim JK, Silwal P, Sasakawa C, Jo EK. An update on the regulatory mechanisms of NLRP3 inflammasome activation. Cell Mol Immunol 2021, 18(5): 1141-1160.

8. Kelley N, Jeltema D, Duan Y, He Y. The NLRP3 Inflammasome: An Overview of Mechanisms of Activation and Regulation. Int J Mol Sci 2019, 20(13).

9. Swanson KV, Deng M, Ting JP. The NLRP3 inflammasome: molecular activation and regulation to therapeutics. Nat Rev Immunol 2019, 19(8): 477-489.

10. Bauernfeind FG, Horvath G, Stutz A, Alnemri ES, MacDonald K, Speert D, et al. Cutting edge: NF-kappaB activating pattern recognition and cytokine receptors license NLRP3 inflammasome activation by regulating NLRP3 expression. J Immunol 2009, 183(2): 787-791.

11. Cornut M, Bourdonnay E, Henry T. Transcriptional Regulation of Inflammasomes. Int J Mol Sci 2020, 21(21).

12. Baker PJ, De Nardo D, Moghaddas F, Tran LS, Bachem A, Nguyen T, et al. Posttranslational Modification as a Critical Determinant of Cytoplasmic Innate Immune Recognition. Physiol Rev 2017, 97(3): 1165-1209.

13. Moretti J, Blander JM. Increasing complexity of NLRP3 inflammasome regulation. J Leukoc Biol 2021, 109(3): 561-571.

14. Liu Y, Yang D, Liu T, Chen J, Yu J, Yi P. N6-methyladenosine-mediated gene regulation and therapeutic implications. Trends Mol Med 2023, 29(6): 454-467.

15. Zaccara S, Ries RJ, Jaffrey SR. Reading, writing and erasing mRNA methylation. Nat Rev Mol Cell Biol 2019, 20(10): 608-624.

16. Zhao Y, Shi Y, Shen H, Xie W. m(6)A-binding proteins: the emerging crucial performers in epigenetics. J Hematol Oncol 2020, 13(1): 35.

17. Xu H, Lin C, Yang J, Chen X, Chen Y, Chen J, et al. The Role of N(6)-Methyladenosine in Inflammatory Diseases. Oxid Med Cell Longev 2022, 2022: 9744771.

18. Peng Z, Gong Y, Wang X, He W, Wu L, Zhang L, et al. METTL3-m(6)A-Rubicon axis inhibits autophagy in nonalcoholic fatty liver disease. Mol Ther 2022, 30(2): 932-946.

19. Jiao Y, Zhang T, Zhang C, Ji H, Tong X, Xia R, et al. Exosomal miR-30d-5p of neutrophils induces M1 macrophage polarization and primes macrophage pyroptosis in sepsis-related acute lung injury. Crit Care 2021, 25(1): 356.

1. Meyer NJ, Gattinoni L, Calfee CS. Acute respiratory distress syndrome. Lancet 2021, 398(10300): 622-637.

2. Gorman EA, O'Kane CM, McAuley DF. Acute respiratory distress syndrome in adults: diagnosis, outcomes, long-term sequelae, and management. Lancet 2022, 400(10358): 1157-1170.

3. Bos LDJ, Ware LB. Acute respiratory distress syndrome: causes, pathophysiology, and phenotypes. Lancet 2022, 400(10358): 1145-1156.

4. Gilroy DW, De Maeyer RPH, Tepper M, O'Brien A, Uddin M, Chen J, et al. Treating exuberant, non-resolving inflammation in the lung; Implications for acute respiratory distress syndrome and COVID-19. Pharmacol Ther 2021, 221: 107745.

5. Laffey JG, Matthay MA. Fifty Years of Research in ARDS. Cell-based Therapy for Acute Respiratory Distress Syndrome. Biology and Potential Therapeutic Value. Am J Respir Crit Care Med 2017, 196(3): 266-273.

6. Xian H, Liu Y, Rundberg Nilsson A, Gatchalian R, Crother TR, Tourtellotte WG, et al. Metformin inhibition of mitochondrial ATP and DNA synthesis abrogates NLRP3 inflammasome activation and pulmonary inflammation. Immunity 2021, 54(7): 1463-1477 e1411.

7. Paik S, Kim JK, Silwal P, Sasakawa C, Jo EK. An update on the regulatory mechanisms of NLRP3 inflammasome activation. Cell Mol Immunol 2021, 18(5): 1141-1160.

8. Kelley N, Jeltema D, Duan Y, He Y. The NLRP3 Inflammasome: An Overview of Mechanisms of Activation and Regulation. Int J Mol Sci 2019, 20(13).

9. Swanson KV, Deng M, Ting JP. The NLRP3 inflammasome: molecular activation and regulation to therapeutics. Nat Rev Immunol 2019, 19(8): 477-489.

10. Bauernfeind FG, Horvath G, Stutz A, Alnemri ES, MacDonald K, Speert D, et al. Cutting edge: NF-kappaB activating pattern recognition and cytokine receptors license NLRP3 inflammasome activation by regulating NLRP3 expression. J Immunol 2009, 183(2): 787-791.

11. Cornut M, Bourdonnay E, Henry T. Transcriptional Regulation of Inflammasomes. Int J Mol Sci 2020, 21(21).

12. Baker PJ, De Nardo D, Moghaddas F, Tran LS, Bachem A, Nguyen T, et al. Posttranslational Modification as a Critical Determinant of Cytoplasmic Innate Immune Recognition. Physiol Rev 2017, 97(3): 1165-1209.

13. Moretti J, Blander JM. Increasing complexity of NLRP3 inflammasome regulation. J Leukoc Biol 2021, 109(3): 561-571.

14. Liu Y, Yang D, Liu T, Chen J, Yu J, Yi P. N6-methyladenosine-mediated gene regulation and therapeutic implications. Trends Mol Med 2023, 29(6): 454-467.

15. Zaccara S, Ries RJ, Jaffrey SR. Reading, writing and erasing mRNA methylation. Nat Rev Mol Cell Biol 2019, 20(10): 608-624.

16. Zhao Y, Shi Y, Shen H, Xie W. m(6)A-binding proteins: the emerging crucial performers in epigenetics. J Hematol Oncol 2020, 13(1): 35.

17. Xu H, Lin C, Yang J, Chen X, Chen Y, Chen J, et al. The Role of N(6)-Methyladenosine in Inflammatory Diseases. Oxid Med Cell Longev 2022, 2022: 9744771.

18. Peng Z, Gong Y, Wang X, He W, Wu L, Zhang L, et al. METTL3-m(6)A-Rubicon axis inhibits autophagy in nonalcoholic fatty liver disease. Mol Ther 2022, 30(2): 932-946.

19. Zhang J, Song B, Zeng Y, Xu C, Gao L, Guo Y, et al. m6A modification in inflammatory bowel disease provides new insights into clinical applications. Biomed Pharmacother 2023, 159: 114298.

20. Jiao Y, Zhang T, Zhang C, Ji H, Tong X, Xia R, et al. Exosomal miR-30d-5p of neutrophils induces M1 macrophage polarization and primes macrophage pyroptosis in sepsis-related acute lung injury. Crit Care 2021, 25(1): 356.

1. Meyer NJ, Gattinoni L, Calfee CS. Acute respiratory distress syndrome. Lancet 2021, 398(10300): 622-637.

2. Gorman EA, O'Kane CM, McAuley DF. Acute respiratory distress syndrome in adults: diagnosis, outcomes, long-term sequelae, and management. Lancet 2022, 400(10358): 1157-1170.

3. Bos LDJ, Ware LB. Acute respiratory distress syndrome: causes, pathophysiology, and phenotypes. Lancet 2022, 400(10358): 1145-1156.

4. Gilroy DW, De Maeyer RPH, Tepper M, O'Brien A, Uddin M, Chen J, et al. Treating exuberant, non-resolving inflammation in the lung; Implications for acute respiratory distress syndrome and COVID-19. Pharmacol Ther 2021, 221: 107745.

5. Laffey JG, Matthay MA. Fifty Years of Research in ARDS. Cell-based Therapy for Acute Respiratory Distress Syndrome. Biology and Potential Therapeutic Value. Am J Respir Crit Care Med 2017, 196(3): 266-273.

6. Xian H, Liu Y, Rundberg Nilsson A, Gatchalian R, Crother TR, Tourtellotte WG, et al. Metformin inhibition of mitochondrial ATP and DNA synthesis abrogates NLRP3 inflammasome activation and pulmonary inflammation. Immunity 2021, 54(7): 1463-1477 e1411.

7. Paik S, Kim JK, Silwal P, Sasakawa C, Jo EK. An update on the regulatory mechanisms of NLRP3 inflammasome activation. Cell Mol Immunol 2021, 18(5): 1141-1160.

8. Kelley N, Jeltema D, Duan Y, He Y. The NLRP3 Inflammasome: An Overview of Mechanisms of Activation and Regulation. Int J Mol Sci 2019, 20(13).

9. Swanson KV, Deng M, Ting JP. The NLRP3 inflammasome: molecular activation and regulation to therapeutics. Nat Rev Immunol 2019, 19(8): 477-489.

10. Bauernfeind FG, Horvath G, Stutz A, Alnemri ES, MacDonald K, Speert D, et al. Cutting edge: NF-kappaB activating pattern recognition and cytokine receptors license NLRP3 inflammasome activation by regulating NLRP3 expression. J Immunol 2009, 183(2): 787-791.

11. Cornut M, Bourdonnay E, Henry T. Transcriptional Regulation of Inflammasomes. Int J Mol Sci 2020, 21(21).

12. Baker PJ, De Nardo D, Moghaddas F, Tran LS, Bachem A, Nguyen T, et al. Posttranslational Modification as a Critical Determinant of Cytoplasmic Innate Immune Recognition. Physiol Rev 2017, 97(3): 1165-1209.

13. Moretti J, Blander JM. Increasing complexity of NLRP3 inflammasome regulation. J Leukoc Biol 2021, 109(3): 561-571.

14. Liu Y, Yang D, Liu T, Chen J, Yu J, Yi P. N6-methyladenosine-mediated gene regulation and therapeutic implications. Trends Mol Med 2023, 29(6): 454-467.

15. Zaccara S, Ries RJ, Jaffrey SR. Reading, writing and erasing mRNA methylation. Nat Rev Mol Cell Biol 2019, 20(10): 608-624.

16. Zhao Y, Shi Y, Shen H, Xie W. m(6)A-binding proteins: the emerging crucial performers in epigenetics. J Hematol Oncol 2020, 13(1): 35.

17. Xu H, Lin C, Yang J, Chen X, Chen Y, Chen J, et al. The Role of N(6)-Methyladenosine in Inflammatory Diseases. Oxid Med Cell Longev 2022, 2022: 9744771.

18. Peng Z, Gong Y, Wang X, He W, Wu L, Zhang L, et al. METTL3-m(6)A-Rubicon axis inhibits autophagy in nonalcoholic fatty liver disease. Mol Ther 2022, 30(2): 932-946.

19. Zhang J, Song B, Zeng Y, Xu C, Gao L, Guo Y, et al. m6A modification in inflammatory bowel disease provides new insights into clinical applications. Biomed Pharmacother 2023, 159: 114298.

20. Winkler R, Gillis E, Lasman L, Safra M, Geula S, Soyris C, et al. m(6)A modification controls the innate immune response to infection by targeting type I interferons. Nat Immunol 2019, 20(2): 173-182.

21. Jiao Y, Zhang T, Zhang C, Ji H, Tong X, Xia R, et al. Exosomal miR-30d-5p of neutrophils induces M1 macrophage polarization and primes macrophage pyroptosis in sepsis-related acute lung injury. Crit Care 2021, 25(1): 356.

1. Meyer NJ, Gattinoni L, Calfee CS. Acute respiratory distress syndrome. Lancet 2021, 398(10300): 622-637.

2. Gorman EA, O'Kane CM, McAuley DF. Acute respiratory distress syndrome in adults: diagnosis, outcomes, long-term sequelae, and management. Lancet 2022, 400(10358): 1157-1170.

3. Bos LDJ, Ware LB. Acute respiratory distress syndrome: causes, pathophysiology, and phenotypes. Lancet 2022, 400(10358): 1145-1156.

4. Gilroy DW, De Maeyer RPH, Tepper M, O'Brien A, Uddin M, Chen J, et al. Treating exuberant, non-resolving inflammation in the lung; Implications for acute respiratory distress syndrome and COVID-19. Pharmacol Ther 2021, 221: 107745.

5. Laffey JG, Matthay MA. Fifty Years of Research in ARDS. Cell-based Therapy for Acute Respiratory Distress Syndrome. Biology and Potential Therapeutic Value. Am J Respir Crit Care Med 2017, 196(3): 266-273.

6. Xian H, Liu Y, Rundberg Nilsson A, Gatchalian R, Crother TR, Tourtellotte WG, et al. Metformin inhibition of mitochondrial ATP and DNA synthesis abrogates NLRP3 inflammasome activation and pulmonary inflammation. Immunity 2021, 54(7): 1463-1477 e1411.

7. Paik S, Kim JK, Silwal P, Sasakawa C, Jo EK. An update on the regulatory mechanisms of NLRP3 inflammasome activation. Cell Mol Immunol 2021, 18(5): 1141-1160.

8. Kelley N, Jeltema D, Duan Y, He Y. The NLRP3 Inflammasome: An Overview of Mechanisms of Activation and Regulation. Int J Mol Sci 2019, 20(13).

9. Swanson KV, Deng M, Ting JP. The NLRP3 inflammasome: molecular activation and regulation to therapeutics. Nat Rev Immunol 2019, 19(8): 477-489.

11. Cornut M, Bourdonnay E, Henry T. Transcriptional Regulation of Inflammasomes. Int J Mol Sci 2020, 21(21).

12. Baker PJ, De Nardo D, Moghaddas F, Tran LS, Bachem A, Nguyen T, et al. Posttranslational Modification as a Critical Determinant of Cytoplasmic Innate Immune Recognition. Physiol Rev 2017, 97(3): 1165-1209.

13. Moretti J, Blander JM. Increasing complexity of NLRP3 inflammasome regulation. J Leukoc Biol 2021, 109(3): 561-571.

14. Liu Y, Yang D, Liu T, Chen J, Yu J, Yi P. N6-methyladenosine-mediated gene regulation and therapeutic implications. Trends Mol Med 2023, 29(6): 454-467.

15. Zaccara S, Ries RJ, Jaffrey SR. Reading, writing and erasing mRNA methylation. Nat Rev Mol Cell Biol 2019, 20(10): 608-624.

16. Zhao Y, Shi Y, Shen H, Xie W. m(6)A-binding proteins: the emerging crucial performers in epigenetics. J Hematol Oncol 2020, 13(1): 35.

17. Xu H, Lin C, Yang J, Chen X, Chen Y, Chen J, et al. The Role of N(6)-Methyladenosine in Inflammatory Diseases. Oxid Med Cell Longev 2022, 2022: 9744771.

18. Peng Z, Gong Y, Wang X, He W, Wu L, Zhang L, et al. METTL3-m(6)A-Rubicon axis inhibits autophagy in nonalcoholic fatty liver disease. Mol Ther 2022, 30(2): 932-946.

19. Zhang J, Song B, Zeng Y, Xu C, Gao L, Guo Y, et al. m6A modification in inflammatory bowel disease provides new insights into clinical applications. Biomed Pharmacother 2023, 159: 114298.

20. Winkler R, Gillis E, Lasman L, Safra M, Geula S, Soyris C, et al. m(6)A modification controls the innate immune response to infection by targeting type I interferons. Nat Immunol 2019, 20(2): 173-182.

21. Zhang H, Liu J, Zhou Y, Qu M, Wang Y, Guo K, et al. Neutrophil extracellular traps mediate m(6)A modification and regulates sepsis-associated acute lung injury by activating ferroptosis in alveolar epithelial cells. Int J Biol Sci 2022, 18(8): 3337-3357.

22. Jiao Y, Zhang T, Zhang C, Ji H, Tong X, Xia R, et al. Exosomal miR-30d-5p of neutrophils induces M1 macrophage polarization and primes macrophage pyroptosis in sepsis-related acute lung injury. Crit Care 2021, 25(1): 356.

1. Meyer NJ, Gattinoni L, Calfee CS. Acute respiratory distress syndrome. Lancet 2021, 398(10300): 622-637.

2. Gorman EA, O'Kane CM, McAuley DF. Acute respiratory distress syndrome in adults: diagnosis, outcomes, long-term sequelae, and management. Lancet 2022, 400(10358): 1157-1170.

3. Bos LDJ, Ware LB. Acute respiratory distress syndrome: causes, pathophysiology, and phenotypes. Lancet 2022, 400(10358): 1145-1156.

4. Gilroy DW, De Maeyer RPH, Tepper M, O'Brien A, Uddin M, Chen J, et al. Treating exuberant, non-resolving inflammation in the lung; Implications for acute respiratory distress syndrome and COVID-19. Pharmacol Ther 2021, 221: 107745.

5. Laffey JG, Matthay MA. Fifty Years of Research in ARDS. Cell-based Therapy for Acute Respiratory Distress Syndrome. Biology and Potential Therapeutic Value. Am J Respir Crit Care Med 2017, 196(3): 266-273.

6. Xian H, Liu Y, Rundberg Nilsson A, Gatchalian R, Crother TR, Tourtellotte WG, et al. Metformin inhibition of mitochondrial ATP and DNA synthesis abrogates NLRP3 inflammasome activation and pulmonary inflammation. Immunity 2021, 54(7): 1463-1477 e1411.

7. Paik S, Kim JK, Silwal P, Sasakawa C, Jo EK. An update on the regulatory mechanisms of NLRP3 inflammasome activation. Cell Mol Immunol 2021, 18(5): 1141-1160.

8. Kelley N, Jeltema D, Duan Y, He Y. The NLRP3 Inflammasome: An Overview of Mechanisms of Activation and Regulation. Int J Mol Sci 2019, 20(13).

9. Swanson KV, Deng M, Ting JP. The NLRP3 inflammasome: molecular activation and regulation to therapeutics. Nat Rev Immunol 2019, 19(8): 477-489.

10. Bauernfeind FG, Horvath G, Stutz A, Alnemri ES, MacDonald K, Speert D, et al. Cutting edge: NF-kappaB activating pattern recognition and cytokine receptors license NLRP3 inflammasome activation by regulating NLRP3 expression. J Immunol 2009, 183(2): 787-791.

11. Cornut M, Bourdonnay E, Henry T. Transcriptional Regulation of Inflammasomes. Int J Mol Sci 2020, 21(21).

12. Baker PJ, De Nardo D, Moghaddas F, Tran LS, Bachem A, Nguyen T, et al. Posttranslational Modification as a Critical Determinant of Cytoplasmic Innate Immune Recognition. Physiol Rev 2017, 97(3): 1165-1209.

13. Moretti J, Blander JM. Increasing complexity of NLRP3 inflammasome regulation. J Leukoc Biol 2021, 109(3): 561-571.

14. Liu Y, Yang D, Liu T, Chen J, Yu J, Yi P. N6-methyladenosine-mediated gene regulation and therapeutic implications. Trends Mol Med 2023, 29(6): 454-467.

15. Zaccara S, Ries RJ, Jaffrey SR. Reading, writing and erasing mRNA methylation. Nat Rev Mol Cell Biol 2019, 20(10): 608-624.

16. Zhao Y, Shi Y, Shen H, Xie W. m(6)A-binding proteins: the emerging crucial performers in epigenetics. J Hematol Oncol 2020, 13(1): 35.

17. Xu H, Lin C, Yang J, Chen X, Chen Y, Chen J, et al. The Role of N(6)-Methyladenosine in Inflammatory Diseases. Oxid Med Cell Longev 2022, 2022: 9744771.

18. Peng Z, Gong Y, Wang X, He W, Wu L, Zhang L, et al. METTL3-m(6)A-Rubicon axis inhibits autophagy in nonalcoholic fatty liver disease. Mol Ther 2022, 30(2): 932-946.

19. Zhang J, Song B, Zeng Y, Xu C, Gao L, Guo Y, et al. m6A modification in inflammatory bowel disease provides new insights into clinical applications. Biomed Pharmacother 2023, 159: 114298.

20. Winkler R, Gillis E, Lasman L, Safra M, Geula S, Soyris C, et al. m(6)A modification controls the innate immune response to infection by targeting type I interferons. Nat Immunol 2019, 20(2): 173-182.

21. Zhang H, Liu J, Zhou Y, Qu M, Wang Y, Guo K, et al. Neutrophil extracellular traps mediate m(6)A modification and regulates sepsis-associated acute lung injury by activating ferroptosis in alveolar epithelial cells. Int J Biol Sci 2022, 18(8): 3337-3357.

22. Jiao Y, Zhang T, Zhang C, Ji H, Tong X, Xia R, et al. Exosomal miR-30d-5p of neutrophils induces M1 macrophage polarization and primes macrophage pyroptosis in sepsis-related acute lung injury. Crit Care 2021, 25(1): 356.

1. Meyer NJ, Gattinoni L, Calfee CS. Acute respiratory distress syndrome. Lancet 2021, 398(10300): 622-637.

2. Gorman EA, O'Kane CM, McAuley DF. Acute respiratory distress syndrome in adults: diagnosis, outcomes, long-term sequelae, and management. Lancet 2022, 400(10358): 1157-1170.

3. Bos LDJ, Ware LB. Acute respiratory distress syndrome: causes, pathophysiology, and phenotypes. Lancet 2022, 400(10358): 1145-1156.

4. Gilroy DW, De Maeyer RPH, Tepper M, O'Brien A, Uddin M, Chen J, et al. Treating exuberant, non-resolving inflammation in the lung; Implications for acute respiratory distress syndrome and COVID-19. Pharmacol Ther 2021, 221: 107745.

5. Laffey JG, Matthay MA. Fifty Years of Research in ARDS. Cell-based Therapy for Acute Respiratory Distress Syndrome. Biology and Potential Therapeutic Value. Am J Respir Crit Care Med 2017, 196(3): 266-273.

6. Xian H, Liu Y, Rundberg Nilsson A, Gatchalian R, Crother TR, Tourtellotte WG, et al. Metformin inhibition of mitochondrial ATP and DNA synthesis abrogates NLRP3 inflammasome activation and pulmonary inflammation. Immunity 2021, 54(7): 1463-1477 e1411.

7. Paik S, Kim JK, Silwal P, Sasakawa C, Jo EK. An update on the regulatory mechanisms of NLRP3 inflammasome activation. Cell Mol Immunol 2021, 18(5): 1141-1160.

8. Kelley N, Jeltema D, Duan Y, He Y. The NLRP3 Inflammasome: An Overview of Mechanisms of Activation and Regulation. Int J Mol Sci 2019, 20(13).

9. Swanson KV, Deng M, Ting JP. The NLRP3 inflammasome: molecular activation and regulation to therapeutics. Nat Rev Immunol 2019, 19(8): 477-489.

10. Bauernfeind FG, Horvath G, Stutz A, Alnemri ES, MacDonald K, Speert D, et al. Cutting edge: NF-kappaB activating pattern recognition and cytokine receptors license NLRP3 inflammasome activation by regulating NLRP3 expression. J Immunol 2009, 183(2): 787-791.

11. Cornut M, Bourdonnay E, Henry T. Transcriptional Regulation of Inflammasomes. Int J Mol Sci 2020, 21(21).

12. Baker PJ, De Nardo D, Moghaddas F, Tran LS, Bachem A, Nguyen T, et al. Posttranslational Modification as a Critical Determinant of Cytoplasmic Innate Immune Recognition. Physiol Rev 2017, 97(3): 1165-1209.

13. Moretti J, Blander JM. Increasing complexity of NLRP3 inflammasome regulation. J Leukoc Biol 2021, 109(3): 561-571.

14. Liu Y, Yang D, Liu T, Chen J, Yu J, Yi P. N6-methyladenosine-mediated gene regulation and therapeutic implications. Trends Mol Med 2023, 29(6): 454-467.

15. Zaccara S, Ries RJ, Jaffrey SR. Reading, writing and erasing mRNA methylation. Nat Rev Mol Cell Biol 2019, 20(10): 608-624.

16. Zhao Y, Shi Y, Shen H, Xie W. m(6)A-binding proteins: the emerging crucial performers in epigenetics. J Hematol Oncol 2020, 13(1): 35.

17. Xu H, Lin C, Yang J, Chen X, Chen Y, Chen J, et al. The Role of N(6)-Methyladenosine in Inflammatory Diseases. Oxid Med Cell Longev 2022, 2022: 9744771.

18. Peng Z, Gong Y, Wang X, He W, Wu L, Zhang L, et al. METTL3-m(6)A-Rubicon axis inhibits autophagy in nonalcoholic fatty liver disease. Mol Ther 2022, 30(2): 932-946.

19. Zhang J, Song B, Zeng Y, Xu C, Gao L, Guo Y, et al. m6A modification in inflammatory bowel disease provides new insights into clinical applications. Biomed Pharmacother 2023, 159: 114298.

20. Winkler R, Gillis E, Lasman L, Safra M, Geula S, Soyris C, et al. m(6)A modification controls the innate immune response to infection by targeting type I interferons. Nat Immunol 2019, 20(2): 173-182.

21. Zhang H, Liu J, Zhou Y, Qu M, Wang Y, Guo K, et al. Neutrophil extracellular traps mediate m(6)A modification and regulates sepsis-associated acute lung injury by activating ferroptosis in alveolar epithelial cells. Int J Biol Sci 2022, 18(8): 3337-3357.

22. Jiao Y, Zhang T, Zhang C, Ji H, Tong X, Xia R, et al. Exosomal miR-30d-5p of neutrophils induces M1 macrophage polarization and primes macrophage pyroptosis in sepsis-related acute lung injury. Crit Care 2021, 25(1): 356.

23. Huang X, Zhang H, Guo X, Zhu Z, Cai H, Kong X. Insulin-like growth factor 2 mRNA-binding protein 1 (IGF2BP1) in cancer. J Hematol Oncol 2018, 11(1): 88.

1. Meyer NJ, Gattinoni L, Calfee CS. Acute respiratory distress syndrome. Lancet 2021, 398(10300): 622-637.

2. Gorman EA, O'Kane CM, McAuley DF. Acute respiratory distress syndrome in adults: diagnosis, outcomes, long-term sequelae, and management. Lancet 2022, 400(10358): 1157-1170.

3. Bos LDJ, Ware LB. Acute respiratory distress syndrome: causes, pathophysiology, and phenotypes. Lancet 2022, 400(10358): 1145-1156.

4. Gilroy DW, De Maeyer RPH, Tepper M, O'Brien A, Uddin M, Chen J, et al. Treating exuberant, non-resolving inflammation in the lung; Implications for acute respiratory distress syndrome and COVID-19. Pharmacol Ther 2021, 221: 107745.

5. Laffey JG, Matthay MA. Fifty Years of Research in ARDS. Cell-based Therapy for Acute Respiratory Distress Syndrome. Biology and Potential Therapeutic Value. Am J Respir Crit Care Med 2017, 196(3): 266-273.

6. Xian H, Liu Y, Rundberg Nilsson A, Gatchalian R, Crother TR, Tourtellotte WG, et al. Metformin inhibition of mitochondrial ATP and DNA synthesis abrogates NLRP3 inflammasome activation and pulmonary inflammation. Immunity 2021, 54(7): 1463-1477 e1411.

7. Paik S, Kim JK, Silwal P, Sasakawa C, Jo EK. An update on the regulatory mechanisms of NLRP3 inflammasome activation. Cell Mol Immunol 2021, 18(5): 1141-1160.

8. Kelley N, Jeltema D, Duan Y, He Y. The NLRP3 Inflammasome: An Overview of Mechanisms of Activation and Regulation. Int J Mol Sci 2019, 20(13).

9. Swanson KV, Deng M, Ting JP. The NLRP3 inflammasome: molecular activation and regulation to therapeutics. Nat Rev Immunol 2019, 19(8): 477-489.

10. Bauernfeind FG, Horvath G, Stutz A, Alnemri ES, MacDonald K, Speert D, et al. Cutting edge: NF-kappaB activating pattern recognition and cytokine receptors license NLRP3 inflammasome activation by regulating NLRP3 expression. J Immunol 2009, 183(2): 787-791.

11. Cornut M, Bourdonnay E, Henry T. Transcriptional Regulation of Inflammasomes. Int J Mol Sci 2020, 21(21).

12. Baker PJ, De Nardo D, Moghaddas F, Tran LS, Bachem A, Nguyen T, et al. Posttranslational Modification as a Critical Determinant of Cytoplasmic Innate Immune Recognition. Physiol Rev 2017, 97(3): 1165-1209.

13. Moretti J, Blander JM. Increasing complexity of NLRP3 inflammasome regulation. J Leukoc Biol 2021, 109(3): 561-571.

14. Liu Y, Yang D, Liu T, Chen J, Yu J, Yi P. N6-methyladenosine-mediated gene regulation and therapeutic implications. Trends Mol Med 2023, 29(6): 454-467.

15. Zaccara S, Ries RJ, Jaffrey SR. Reading, writing and erasing mRNA methylation. Nat Rev Mol Cell Biol 2019, 20(10): 608-624.

16. Zhao Y, Shi Y, Shen H, Xie W. m(6)A-binding proteins: the emerging crucial performers in epigenetics. J Hematol Oncol 2020, 13(1): 35.

17. Xu H, Lin C, Yang J, Chen X, Chen Y, Chen J, et al. The Role of N(6)-Methyladenosine in Inflammatory Diseases. Oxid Med Cell Longev 2022, 2022: 9744771.

18. Peng Z, Gong Y, Wang X, He W, Wu L, Zhang L, et al. METTL3-m(6)A-Rubicon axis inhibits autophagy in nonalcoholic fatty liver disease. Mol Ther 2022, 30(2): 932-946.

19. Zhang J, Song B, Zeng Y, Xu C, Gao L, Guo Y, et al. m6A modification in inflammatory bowel disease provides new insights into clinical applications. Biomed Pharmacother 2023, 159: 114298.

20. Winkler R, Gillis E, Lasman L, Safra M, Geula S, Soyris C, et al. m(6)A modification controls the innate immune response to infection by targeting type I interferons. Nat Immunol 2019, 20(2): 173-182.

21. Zhang H, Liu J, Zhou Y, Qu M, Wang Y, Guo K, et al. Neutrophil extracellular traps mediate m(6)A modification and regulates sepsis-associated acute lung injury by activating ferroptosis in alveolar epithelial cells. Int J Biol Sci 2022, 18(8): 3337-3357.

22. Jiao Y, Zhang T, Zhang C, Ji H, Tong X, Xia R, et al. Exosomal miR-30d-5p of neutrophils induces M1 macrophage polarization and primes macrophage pyroptosis in sepsis-related acute lung injury. Crit Care 2021, 25(1): 356.

23. Huang X, Zhang H, Guo X, Zhu Z, Cai H, Kong X. Insulin-like growth factor 2 mRNA-binding protein 1 (IGF2BP1) in cancer. J Hematol Oncol 2018, 11(1): 88.

24. Huang H, Weng H, Chen J. m(6)A Modification in Coding and Non-coding RNAs: Roles and Therapeutic Implications in Cancer. Cancer Cell 2020, 37(3): 270-288.

1. Meyer NJ, Gattinoni L, Calfee CS. Acute respiratory distress syndrome. Lancet 2021, 398(10300): 622-637.

2. Gorman EA, O'Kane CM, McAuley DF. Acute respiratory distress syndrome in adults: diagnosis, outcomes, long-term sequelae, and management. Lancet 2022, 400(10358): 1157-1170.

3. Bos LDJ, Ware LB. Acute respiratory distress syndrome: causes, pathophysiology, and phenotypes. Lancet 2022, 400(10358): 1145-1156.

4. Gilroy DW, De Maeyer RPH, Tepper M, O'Brien A, Uddin M, Chen J, et al. Treating exuberant, non-resolving inflammation in the lung; Implications for acute respiratory distress syndrome and COVID-19. Pharmacol Ther 2021, 221: 107745.

5. Laffey JG, Matthay MA. Fifty Years of Research in ARDS. Cell-based Therapy for Acute Respiratory Distress Syndrome. Biology and Potential Therapeutic Value. Am J Respir Crit Care Med 2017, 196(3): 266-273.

6. Xian H, Liu Y, Rundberg Nilsson A, Gatchalian R, Crother TR, Tourtellotte WG, et al. Metformin inhibition of mitochondrial ATP and DNA synthesis abrogates NLRP3 inflammasome activation and pulmonary inflammation. Immunity 2021, 54(7): 1463-1477 e1411.

7. Paik S, Kim JK, Silwal P, Sasakawa C, Jo EK. An update on the regulatory mechanisms of NLRP3 inflammasome activation. Cell Mol Immunol 2021, 18(5): 1141-1160.

8. Kelley N, Jeltema D, Duan Y, He Y. The NLRP3 Inflammasome: An Overview of Mechanisms of Activation and Regulation. Int J Mol Sci 2019, 20(13).

9. Swanson KV, Deng M, Ting JP. The NLRP3 inflammasome: molecular activation and regulation to therapeutics. Nat Rev Immunol 2019, 19(8): 477-489.

10. Bauernfeind FG, Horvath G, Stutz A, Alnemri ES, MacDonald K, Speert D, et al. Cutting edge: NF-kappaB activating pattern recognition and cytokine receptors license NLRP3 inflammasome activation by regulating NLRP3 expression. J Immunol 2009, 183(2): 787-791.

11. Cornut M, Bourdonnay E, Henry T. Transcriptional Regulation of Inflammasomes. Int J Mol Sci 2020, 21(21).

12. Baker PJ, De Nardo D, Moghaddas F, Tran LS, Bachem A, Nguyen T, et al. Posttranslational Modification as a Critical Determinant of Cytoplasmic Innate Immune Recognition. Physiol Rev 2017, 97(3): 1165-1209.

13. Moretti J, Blander JM. Increasing complexity of NLRP3 inflammasome regulation. J Leukoc Biol 2021, 109(3): 561-571.

14. Liu Y, Yang D, Liu T, Chen J, Yu J, Yi P. N6-methyladenosine-mediated gene regulation and therapeutic implications. Trends Mol Med 2023, 29(6): 454-467.

15. Zaccara S, Ries RJ, Jaffrey SR. Reading, writing and erasing mRNA methylation. Nat Rev Mol Cell Biol 2019, 20(10): 608-624.

16. Zhao Y, Shi Y, Shen H, Xie W. m(6)A-binding proteins: the emerging crucial performers in epigenetics. J Hematol Oncol 2020, 13(1): 35.

17. Xu H, Lin C, Yang J, Chen X, Chen Y, Chen J, et al. The Role of N(6)-Methyladenosine in Inflammatory Diseases. Oxid Med Cell Longev 2022, 2022: 9744771.

18. Peng Z, Gong Y, Wang X, He W, Wu L, Zhang L, et al. METTL3-m(6)A-Rubicon axis inhibits autophagy in nonalcoholic fatty liver disease. Mol Ther 2022, 30(2): 932-946.

19. Zhang J, Song B, Zeng Y, Xu C, Gao L, Guo Y, et al. m6A modification in inflammatory bowel disease provides new insights into clinical applications. Biomed Pharmacother 2023, 159: 114298.

20. Winkler R, Gillis E, Lasman L, Safra M, Geula S, Soyris C, et al. m(6)A modification controls the innate immune response to infection by targeting type I interferons. Nat Immunol 2019, 20(2): 173-182.

21. Zhang H, Liu J, Zhou Y, Qu M, Wang Y, Guo K, et al. Neutrophil extracellular traps mediate m(6)A modification and regulates sepsis-associated acute lung injury by activating ferroptosis in alveolar epithelial cells. Int J Biol Sci 2022, 18(8): 3337-3357.

22. Jiao Y, Zhang T, Zhang C, Ji H, Tong X, Xia R, et al. Exosomal miR-30d-5p of neutrophils induces M1 macrophage polarization and primes macrophage pyroptosis in sepsis-related acute lung injury. Crit Care 2021, 25(1): 356.

23. Huang X, Zhang H, Guo X, Zhu Z, Cai H, Kong X. Insulin-like growth factor 2 mRNA-binding protein 1 (IGF2BP1) in cancer. J Hematol Oncol 2018, 11(1): 88.

24. Huang H, Weng H, Chen J. m(6)A Modification in Coding and Non-coding RNAs: Roles and Therapeutic Implications in Cancer. Cancer Cell 2020, 37(3): 270-288.

25. Zhu X, Tang H, Yang M, Yin K. N6-methyladenosine in macrophage function: a novel target for metabolic diseases. Trends Endocrinol Metab 2023, 34(2): 66-84.

1. Meyer NJ, Gattinoni L, Calfee CS. Acute respiratory distress syndrome. Lancet 2021, 398(10300): 622-637.

2. Gorman EA, O'Kane CM, McAuley DF. Acute respiratory distress syndrome in adults: diagnosis, outcomes, long-term sequelae, and management. Lancet 2022, 400(10358): 1157-1170.

3. Bos LDJ, Ware LB. Acute respiratory distress syndrome: causes, pathophysiology, and phenotypes. Lancet 2022, 400(10358): 1145-1156.

4. Gilroy DW, De Maeyer RPH, Tepper M, O'Brien A, Uddin M, Chen J, et al. Treating exuberant, non-resolving inflammation in the lung; Implications for acute respiratory distress syndrome and COVID-19. Pharmacol Ther 2021, 221: 107745.

5. Laffey JG, Matthay MA. Fifty Years of Research in ARDS. Cell-based Therapy for Acute Respiratory Distress Syndrome. Biology and Potential Therapeutic Value. Am J Respir Crit Care Med 2017, 196(3): 266-273.

6. Xian H, Liu Y, Rundberg Nilsson A, Gatchalian R, Crother TR, Tourtellotte WG, et al. Metformin inhibition of mitochondrial ATP and DNA synthesis abrogates NLRP3 inflammasome activation and pulmonary inflammation. Immunity 2021, 54(7): 1463-1477 e1411.

8. Kelley N, Jeltema D, Duan Y, He Y. The NLRP3 Inflammasome: An Overview of Mechanisms of Activation and Regulation. Int J Mol Sci 2019, 20(13).

9. Swanson KV, Deng M, Ting JP. The NLRP3 inflammasome: molecular activation and regulation to therapeutics. Nat Rev Immunol 2019, 19(8): 477-489.

10. Bauernfeind FG, Horvath G, Stutz A, Alnemri ES, MacDonald K, Speert D, et al. Cutting edge: NF-kappaB activating pattern recognition and cytokine receptors license NLRP3 inflammasome activation by regulating NLRP3 expression. J Immunol 2009, 183(2): 787-791.

11. Cornut M, Bourdonnay E, Henry T. Transcriptional Regulation of Inflammasomes. Int J Mol Sci 2020, 21(21).

12. Baker PJ, De Nardo D, Moghaddas F, Tran LS, Bachem A, Nguyen T, et al. Posttranslational Modification as a Critical Determinant of Cytoplasmic Innate Immune Recognition. Physiol Rev 2017, 97(3): 1165-1209.

13. Moretti J, Blander JM. Increasing complexity of NLRP3 inflammasome regulation. J Leukoc Biol 2021, 109(3): 561-571.

14. Liu Y, Yang D, Liu T, Chen J, Yu J, Yi P. N6-methyladenosine-mediated gene regulation and therapeutic implications. Trends Mol Med 2023, 29(6): 454-467.

15. Zaccara S, Ries RJ, Jaffrey SR. Reading, writing and erasing mRNA methylation. Nat Rev Mol Cell Biol 2019, 20(10): 608-624.

17. Xu H, Lin C, Yang J, Chen X, Chen Y, Chen J, et al. The Role of N(6)-Methyladenosine in Inflammatory Diseases. Oxid Med Cell Longev 2022, 2022: 9744771.

18. Peng Z, Gong Y, Wang X, He W, Wu L, Zhang L, et al. METTL3-m(6)A-Rubicon axis inhibits autophagy in nonalcoholic fatty liver disease. Mol Ther 2022, 30(2): 932-946.

19. Zhang J, Song B, Zeng Y, Xu C, Gao L, Guo Y, et al. m6A modification in inflammatory bowel disease provides new insights into clinical applications. Biomed Pharmacother 2023, 159: 114298.

20. Winkler R, Gillis E, Lasman L, Safra M, Geula S, Soyris C, et al. m(6)A modification controls the innate immune response to infection by targeting type I interferons. Nat Immunol 2019, 20(2): 173-182.

21. Zhang H, Liu J, Zhou Y, Qu M, Wang Y, Guo K, et al. Neutrophil extracellular traps mediate m(6)A modification and regulates sepsis-associated acute lung injury by activating ferroptosis in alveolar epithelial cells. Int J Biol Sci 2022, 18(8): 3337-3357.

22. Jiao Y, Zhang T, Zhang C, Ji H, Tong X, Xia R, et al. Exosomal miR-30d-5p of neutrophils induces M1 macrophage polarization and primes macrophage pyroptosis in sepsis-related acute lung injury. Crit Care 2021, 25(1): 356.

23. Huang X, Zhang H, Guo X, Zhu Z, Cai H, Kong X. Insulin-like growth factor 2 mRNA-binding protein 1 (IGF2BP1) in cancer. J Hematol Oncol 2018, 11(1): 88.

24. Huang H, Weng H, Chen J. m(6)A Modification in Coding and Non-coding RNAs: Roles and Therapeutic Implications in Cancer. Cancer Cell 2020, 37(3): 270-288.

25. Zhu X, Tang H, Yang M, Yin K. N6-methyladenosine in macrophage function: a novel target for metabolic diseases. Trends Endocrinol Metab 2023, 34(2): 66-84.

26. Kumari R, Ranjan P, Suleiman ZG, Goswami SK, Li J, Prasad R, et al. mRNA modifications in cardiovascular biology and disease: with a focus on m6A modification. Cardiovasc Res 2022, 118(7): 1680-1692.

1. Meyer NJ, Gattinoni L, Calfee CS. Acute respiratory distress syndrome. Lancet 2021, 398(10300): 622-637.

2. Gorman EA, O'Kane CM, McAuley DF. Acute respiratory distress syndrome in adults: diagnosis, outcomes, long-term sequelae, and management. Lancet 2022, 400(10358): 1157-1170.

3. Bos LDJ, Ware LB. Acute respiratory distress syndrome: causes, pathophysiology, and phenotypes. Lancet 2022, 400(10358): 1145-1156.

4. Gilroy DW, De Maeyer RPH, Tepper M, O'Brien A, Uddin M, Chen J, et al. Treating exuberant, non-resolving inflammation in the lung; Implications for acute respiratory distress syndrome and COVID-19. Pharmacol Ther 2021, 221: 107745.

5. Laffey JG, Matthay MA. Fifty Years of Research in ARDS. Cell-based Therapy for Acute Respiratory Distress Syndrome. Biology and Potential Therapeutic Value. Am J Respir Crit Care Med 2017, 196(3): 266-273.

6. Xian H, Liu Y, Rundberg Nilsson A, Gatchalian R, Crother TR, Tourtellotte WG, et al. Metformin inhibition of mitochondrial ATP and DNA synthesis abrogates NLRP3 inflammasome activation and pulmonary inflammation. Immunity 2021, 54(7): 1463-1477 e1411.

7. Paik S, Kim JK, Silwal P, Sasakawa C, Jo EK. An update on the regulatory mechanisms of NLRP3 inflammasome activation. Cell Mol Immunol 2021, 18(5): 1141-1160.

8. Kelley N, Jeltema D, Duan Y, He Y. The NLRP3 Inflammasome: An Overview of Mechanisms of Activation and Regulation. Int J Mol Sci 2019, 20(13).

9. Swanson KV, Deng M, Ting JP. The NLRP3 inflammasome: molecular activation and regulation to therapeutics. Nat Rev Immunol 2019, 19(8): 477-489.

10. Bauernfeind FG, Horvath G, Stutz A, Alnemri ES, MacDonald K, Speert D, et al. Cutting edge: NF-kappaB activating pattern recognition and cytokine receptors license NLRP3 inflammasome activation by regulating NLRP3 expression. J Immunol 2009, 183(2): 787-791.

11. Cornut M, Bourdonnay E, Henry T. Transcriptional Regulation of Inflammasomes. Int J Mol Sci 2020, 21(21).

12. Baker PJ, De Nardo D, Moghaddas F, Tran LS, Bachem A, Nguyen T, et al. Posttranslational Modification as a Critical Determinant of Cytoplasmic Innate Immune Recognition. Physiol Rev 2017, 97(3): 1165-1209.

13. Moretti J, Blander JM. Increasing complexity of NLRP3 inflammasome regulation. J Leukoc Biol 2021, 109(3): 561-571.

14. Liu Y, Yang D, Liu T, Chen J, Yu J, Yi P. N6-methyladenosine-mediated gene regulation and therapeutic implications. Trends Mol Med 2023, 29(6): 454-467.

15. Zaccara S, Ries RJ, Jaffrey SR. Reading, writing and erasing mRNA methylation. Nat Rev Mol Cell Biol 2019, 20(10): 608-624.

16. Zhao Y, Shi Y, Shen H, Xie W. m(6)A-binding proteins: the emerging crucial performers in epigenetics. J Hematol Oncol 2020, 13(1): 35.

17. Xu H, Lin C, Yang J, Chen X, Chen Y, Chen J, et al. The Role of N(6)-Methyladenosine in Inflammatory Diseases. Oxid Med Cell Longev 2022, 2022: 9744771.

18. Peng Z, Gong Y, Wang X, He W, Wu L, Zhang L, et al. METTL3-m(6)A-Rubicon axis inhibits autophagy in nonalcoholic fatty liver disease. Mol Ther 2022, 30(2): 932-946.

19. Zhang J, Song B, Zeng Y, Xu C, Gao L, Guo Y, et al. m6A modification in inflammatory bowel disease provides new insights into clinical applications. Biomed Pharmacother 2023, 159: 114298.

20. Winkler R, Gillis E, Lasman L, Safra M, Geula S, Soyris C, et al. m(6)A modification controls the innate immune response to infection by targeting type I interferons. Nat Immunol 2019, 20(2): 173-182.

21. Zhang H, Liu J, Zhou Y, Qu M, Wang Y, Guo K, et al. Neutrophil extracellular traps mediate m(6)A modification and regulates sepsis-associated acute lung injury by activating ferroptosis in alveolar epithelial cells. Int J Biol Sci 2022, 18(8): 3337-3357.

22. Jiao Y, Zhang T, Zhang C, Ji H, Tong X, Xia R, et al. Exosomal miR-30d-5p of neutrophils induces M1 macrophage polarization and primes macrophage pyroptosis in sepsis-related acute lung injury. Crit Care 2021, 25(1): 356.

23. Huang X, Zhang H, Guo X, Zhu Z, Cai H, Kong X. Insulin-like growth factor 2 mRNA-binding protein 1 (IGF2BP1) in cancer. J Hematol Oncol 2018, 11(1): 88.

24. Huang H, Weng H, Chen J. m(6)A Modification in Coding and Non-coding RNAs: Roles and Therapeutic Implications in Cancer. Cancer Cell 2020, 37(3): 270-288.

25. Zhu X, Tang H, Yang M, Yin K. N6-methyladenosine in macrophage function: a novel target for metabolic diseases. Trends Endocrinol Metab 2023, 34(2): 66-84.

26. Kumari R, Ranjan P, Suleiman ZG, Goswami SK, Li J, Prasad R, et al. mRNA modifications in cardiovascular biology and disease: with a focus on m6A modification. Cardiovasc Res 2022, 118(7): 1680-1692.

27. Luo J, Xu T, Sun K. N6-Methyladenosine RNA Modification in Inflammation: Roles, Mechanisms, and Applications. Front Cell Dev Biol 2021, 9: 670711.

1. Meyer NJ, Gattinoni L, Calfee CS. Acute respiratory distress syndrome. Lancet 2021, 398(10300): 622-637.

2. Gorman EA, O'Kane CM, McAuley DF. Acute respiratory distress syndrome in adults: diagnosis, outcomes, long-term sequelae, and management. Lancet 2022, 400(10358): 1157-1170.

3. Bos LDJ, Ware LB. Acute respiratory distress syndrome: causes, pathophysiology, and phenotypes. Lancet 2022, 400(10358): 1145-1156.

4. Gilroy DW, De Maeyer RPH, Tepper M, O'Brien A, Uddin M, Chen J, et al. Treating exuberant, non-resolving inflammation in the lung; Implications for acute respiratory distress syndrome and COVID-19. Pharmacol Ther 2021, 221: 107745.

5. Laffey JG, Matthay MA. Fifty Years of Research in ARDS. Cell-based Therapy for Acute Respiratory Distress Syndrome. Biology and Potential Therapeutic Value. Am J Respir Crit Care Med 2017, 196(3): 266-273.

6. Xian H, Liu Y, Rundberg Nilsson A, Gatchalian R, Crother TR, Tourtellotte WG, et al. Metformin inhibition of mitochondrial ATP and DNA synthesis abrogates NLRP3 inflammasome activation and pulmonary inflammation. Immunity 2021, 54(7): 1463-1477 e1411.

7. Paik S, Kim JK, Silwal P, Sasakawa C, Jo EK. An update on the regulatory mechanisms of NLRP3 inflammasome activation. Cell Mol Immunol 2021, 18(5): 1141-1160.

8. Kelley N, Jeltema D, Duan Y, He Y. The NLRP3 Inflammasome: An Overview of Mechanisms of Activation and Regulation. Int J Mol Sci 2019, 20(13).

9. Swanson KV, Deng M, Ting JP. The NLRP3 inflammasome: molecular activation and regulation to therapeutics. Nat Rev Immunol 2019, 19(8): 477-489.

10. Bauernfeind FG, Horvath G, Stutz A, Alnemri ES, MacDonald K, Speert D, et al. Cutting edge: NF-kappaB activating pattern recognition and cytokine receptors license NLRP3 inflammasome activation by regulating NLRP3 expression. J Immunol 2009, 183(2): 787-791.

11. Cornut M, Bourdonnay E, Henry T. Transcriptional Regulation of Inflammasomes. Int J Mol Sci 2020, 21(21).

12. Baker PJ, De Nardo D, Moghaddas F, Tran LS, Bachem A, Nguyen T, et al. Posttranslational Modification as a Critical Determinant of Cytoplasmic Innate Immune Recognition. Physiol Rev 2017, 97(3): 1165-1209.

13. Moretti J, Blander JM. Increasing complexity of NLRP3 inflammasome regulation. J Leukoc Biol 2021, 109(3): 561-571.

14. Liu Y, Yang D, Liu T, Chen J, Yu J, Yi P. N6-methyladenosine-mediated gene regulation and therapeutic implications. Trends Mol Med 2023, 29(6): 454-467.

15. Zaccara S, Ries RJ, Jaffrey SR. Reading, writing and erasing mRNA methylation. Nat Rev Mol Cell Biol 2019, 20(10): 608-624.

16. Zhao Y, Shi Y, Shen H, Xie W. m(6)A-binding proteins: the emerging crucial performers in epigenetics. J Hematol Oncol 2020, 13(1): 35.

17. Xu H, Lin C, Yang J, Chen X, Chen Y, Chen J, et al. The Role of N(6)-Methyladenosine in Inflammatory Diseases. Oxid Med Cell Longev 2022, 2022: 9744771.

18. Peng Z, Gong Y, Wang X, He W, Wu L, Zhang L, et al. METTL3-m(6)A-Rubicon axis inhibits autophagy in nonalcoholic fatty liver disease. Mol Ther 2022, 30(2): 932-946.

19. Zhang J, Song B, Zeng Y, Xu C, Gao L, Guo Y, et al. m6A modification in inflammatory bowel disease provides new insights into clinical applications. Biomed Pharmacother 2023, 159: 114298.

20. Winkler R, Gillis E, Lasman L, Safra M, Geula S, Soyris C, et al. m(6)A modification controls the innate immune response to infection by targeting type I interferons. Nat Immunol 2019, 20(2): 173-182.

21. Zhang H, Liu J, Zhou Y, Qu M, Wang Y, Guo K, et al. Neutrophil extracellular traps mediate m(6)A modification and regulates sepsis-associated acute lung injury by activating ferroptosis in alveolar epithelial cells. Int J Biol Sci 2022, 18(8): 3337-3357.

22. Jiao Y, Zhang T, Zhang C, Ji H, Tong X, Xia R, et al. Exosomal miR-30d-5p of neutrophils induces M1 macrophage polarization and primes macrophage pyroptosis in sepsis-related acute lung injury. Crit Care 2021, 25(1): 356.

23. Huang X, Zhang H, Guo X, Zhu Z, Cai H, Kong X. Insulin-like growth factor 2 mRNA-binding protein 1 (IGF2BP1) in cancer. J Hematol Oncol 2018, 11(1): 88.

24. Huang H, Weng H, Chen J. m(6)A Modification in Coding and Non-coding RNAs: Roles and Therapeutic Implications in Cancer. Cancer Cell 2020, 37(3): 270-288.

25. Zhu X, Tang H, Yang M, Yin K. N6-methyladenosine in macrophage function: a novel target for metabolic diseases. Trends Endocrinol Metab 2023, 34(2): 66-84.

26. Kumari R, Ranjan P, Suleiman ZG, Goswami SK, Li J, Prasad R, et al. mRNA modifications in cardiovascular biology and disease: with a focus on m6A modification. Cardiovasc Res 2022, 118(7): 1680-1692.

27. Luo J, Xu T, Sun K. N6-Methyladenosine RNA Modification in Inflammation: Roles, Mechanisms, and Applications. Front Cell Dev Biol 2021, 9: 670711.

28. Weng H, Huang H, Wu H, Qin X, Zhao BS, Dong L, et al. METTL14 Inhibits Hematopoietic Stem/Progenitor Differentiation and Promotes Leukemogenesis via mRNA m(6)A Modification. Cell Stem Cell 2018, 22(2): 191-205 e199.

1. Meyer NJ, Gattinoni L, Calfee CS. Acute respiratory distress syndrome. Lancet 2021, 398(10300): 622-637.

2. Gorman EA, O'Kane CM, McAuley DF. Acute respiratory distress syndrome in adults: diagnosis, outcomes, long-term sequelae, and management. Lancet 2022, 400(10358): 1157-1170.

3. Bos LDJ, Ware LB. Acute respiratory distress syndrome: causes, pathophysiology, and phenotypes. Lancet 2022, 400(10358): 1145-1156.

4. Gilroy DW, De Maeyer RPH, Tepper M, O'Brien A, Uddin M, Chen J, et al. Treating exuberant, non-resolving inflammation in the lung; Implications for acute respiratory distress syndrome and COVID-19. Pharmacol Ther 2021, 221: 107745.

5. Laffey JG, Matthay MA. Fifty Years of Research in ARDS. Cell-based Therapy for Acute Respiratory Distress Syndrome. Biology and Potential Therapeutic Value. Am J Respir Crit Care Med 2017, 196(3): 266-273.

6. Xian H, Liu Y, Rundberg Nilsson A, Gatchalian R, Crother TR, Tourtellotte WG, et al. Metformin inhibition of mitochondrial ATP and DNA synthesis abrogates NLRP3 inflammasome activation and pulmonary inflammation. Immunity 2021, 54(7): 1463-1477 e1411.

7. Paik S, Kim JK, Silwal P, Sasakawa C, Jo EK. An update on the regulatory mechanisms of NLRP3 inflammasome activation. Cell Mol Immunol 2021, 18(5): 1141-1160.

8. Kelley N, Jeltema D, Duan Y, He Y. The NLRP3 Inflammasome: An Overview of Mechanisms of Activation and Regulation. Int J Mol Sci 2019, 20(13).

9. Swanson KV, Deng M, Ting JP. The NLRP3 inflammasome: molecular activation and regulation to therapeutics. Nat Rev Immunol 2019, 19(8): 477-489.

10. Bauernfeind FG, Horvath G, Stutz A, Alnemri ES, MacDonald K, Speert D, et al. Cutting edge: NF-kappaB activating pattern recognition and cytokine receptors license NLRP3 inflammasome activation by regulating NLRP3 expression. J Immunol 2009, 183(2): 787-791.

11. Cornut M, Bourdonnay E, Henry T. Transcriptional Regulation of Inflammasomes. Int J Mol Sci 2020, 21(21).

12. Baker PJ, De Nardo D, Moghaddas F, Tran LS, Bachem A, Nguyen T, et al. Posttranslational Modification as a Critical Determinant of Cytoplasmic Innate Immune Recognition. Physiol Rev 2017, 97(3): 1165-1209.

13. Moretti J, Blander JM. Increasing complexity of NLRP3 inflammasome regulation. J Leukoc Biol 2021, 109(3): 561-571.

14. Liu Y, Yang D, Liu T, Chen J, Yu J, Yi P. N6-methyladenosine-mediated gene regulation and therapeutic implications. Trends Mol Med 2023, 29(6): 454-467.

15. Zaccara S, Ries RJ, Jaffrey SR. Reading, writing and erasing mRNA methylation. Nat Rev Mol Cell Biol 2019, 20(10): 608-624.

16. Zhao Y, Shi Y, Shen H, Xie W. m(6)A-binding proteins: the emerging crucial performers in epigenetics. J Hematol Oncol 2020, 13(1): 35.

18. Peng Z, Gong Y, Wang X, He W, Wu L, Zhang L, et al. METTL3-m(6)A-Rubicon axis inhibits autophagy in nonalcoholic fatty liver disease. Mol Ther 2022, 30(2): 932-946.

19. Zhang J, Song B, Zeng Y, Xu C, Gao L, Guo Y, et al. m6A modification in inflammatory bowel disease provides new insights into clinical applications. Biomed Pharmacother 2023, 159: 114298.

20. Winkler R, Gillis E, Lasman L, Safra M, Geula S, Soyris C, et al. m(6)A modification controls the innate immune response to infection by targeting type I interferons. Nat Immunol 2019, 20(2): 173-182.

21. Zhang H, Liu J, Zhou Y, Qu M, Wang Y, Guo K, et al. Neutrophil extracellular traps mediate m(6)A modification and regulates sepsis-associated acute lung injury by activating ferroptosis in alveolar epithelial cells. Int J Biol Sci 2022, 18(8): 3337-3357.

22. Jiao Y, Zhang T, Zhang C, Ji H, Tong X, Xia R, et al. Exosomal miR-30d-5p of neutrophils induces M1 macrophage polarization and primes macrophage pyroptosis in sepsis-related acute lung injury. Crit Care 2021, 25(1): 356.

23. Huang X, Zhang H, Guo X, Zhu Z, Cai H, Kong X. Insulin-like growth factor 2 mRNA-binding protein 1 (IGF2BP1) in cancer. J Hematol Oncol 2018, 11(1): 88.

24. Huang H, Weng H, Chen J. m(6)A Modification in Coding and Non-coding RNAs: Roles and Therapeutic Implications in Cancer. Cancer Cell 2020, 37(3): 270-288.

25. Zhu X, Tang H, Yang M, Yin K. N6-methyladenosine in macrophage function: a novel target for metabolic diseases. Trends Endocrinol Metab 2023, 34(2): 66-84.

26. Kumari R, Ranjan P, Suleiman ZG, Goswami SK, Li J, Prasad R, et al. mRNA modifications in cardiovascular biology and disease: with a focus on m6A modification. Cardiovasc Res 2022, 118(7): 1680-1692.

27. Luo J, Xu T, Sun K. N6-Methyladenosine RNA Modification in Inflammation: Roles, Mechanisms, and Applications. Front Cell Dev Biol 2021, 9: 670711.

28. Weng H, Huang H, Wu H, Qin X, Zhao BS, Dong L, et al. METTL14 Inhibits Hematopoietic Stem/Progenitor Differentiation and Promotes Leukemogenesis via mRNA m(6)A Modification. Cell Stem Cell 2018, 22(2): 191-205 e199.

29. Zhang H, Wu D, Wang Y, Guo K, Spencer CB, Ortoga L, et al. METTL3-mediated N6-methyladenosine exacerbates ferroptosis via m6A-IGF2BP2-dependent mitochondrial metabolic reprogramming in sepsis-induced acute lung injury. Clin Transl Med 2023, 13(9): e1389.

1. Meyer NJ, Gattinoni L, Calfee CS. Acute respiratory distress syndrome. Lancet 2021, 398(10300): 622-637.

2. Gorman EA, O'Kane CM, McAuley DF. Acute respiratory distress syndrome in adults: diagnosis, outcomes, long-term sequelae, and management. Lancet 2022, 400(10358): 1157-1170.

3. Bos LDJ, Ware LB. Acute respiratory distress syndrome: causes, pathophysiology, and phenotypes. Lancet 2022, 400(10358): 1145-1156.

4. Gilroy DW, De Maeyer RPH, Tepper M, O'Brien A, Uddin M, Chen J, et al. Treating exuberant, non-resolving inflammation in the lung; Implications for acute respiratory distress syndrome and COVID-19. Pharmacol Ther 2021, 221: 107745.

5. Laffey JG, Matthay MA. Fifty Years of Research in ARDS. Cell-based Therapy for Acute Respiratory Distress Syndrome. Biology and Potential Therapeutic Value. Am J Respir Crit Care Med 2017, 196(3): 266-273.

6. Xian H, Liu Y, Rundberg Nilsson A, Gatchalian R, Crother TR, Tourtellotte WG, et al. Metformin inhibition of mitochondrial ATP and DNA synthesis abrogates NLRP3 inflammasome activation and pulmonary inflammation. Immunity 2021, 54(7): 1463-1477 e1411.

7. Paik S, Kim JK, Silwal P, Sasakawa C, Jo EK. An update on the regulatory mechanisms of NLRP3 inflammasome activation. Cell Mol Immunol 2021, 18(5): 1141-1160.

8. Kelley N, Jeltema D, Duan Y, He Y. The NLRP3 Inflammasome: An Overview of Mechanisms of Activation and Regulation. Int J Mol Sci 2019, 20(13).

9. Swanson KV, Deng M, Ting JP. The NLRP3 inflammasome: molecular activation and regulation to therapeutics. Nat Rev Immunol 2019, 19(8): 477-489.

10. Bauernfeind FG, Horvath G, Stutz A, Alnemri ES, MacDonald K, Speert D, et al. Cutting edge: NF-kappaB activating pattern recognition and cytokine receptors license NLRP3 inflammasome activation by regulating NLRP3 expression. J Immunol 2009, 183(2): 787-791.

11. Cornut M, Bourdonnay E, Henry T. Transcriptional Regulation of Inflammasomes. Int J Mol Sci 2020, 21(21).

12. Baker PJ, De Nardo D, Moghaddas F, Tran LS, Bachem A, Nguyen T, et al. Posttranslational Modification as a Critical Determinant of Cytoplasmic Innate Immune Recognition. Physiol Rev 2017, 97(3): 1165-1209.

13. Moretti J, Blander JM. Increasing complexity of NLRP3 inflammasome regulation. J Leukoc Biol 2021, 109(3): 561-571.

14. Liu Y, Yang D, Liu T, Chen J, Yu J, Yi P. N6-methyladenosine-mediated gene regulation and therapeutic implications. Trends Mol Med 2023, 29(6): 454-467.

16. Zhao Y, Shi Y, Shen H, Xie W. m(6)A-binding proteins: the emerging crucial performers in epigenetics. J Hematol Oncol 2020, 13(1): 35.

17. Xu H, Lin C, Yang J, Chen X, Chen Y, Chen J, et al. The Role of N(6)-Methyladenosine in Inflammatory Diseases. Oxid Med Cell Longev 2022, 2022: 9744771.

18. Peng Z, Gong Y, Wang X, He W, Wu L, Zhang L, et al. METTL3-m(6)A-Rubicon axis inhibits autophagy in nonalcoholic fatty liver disease. Mol Ther 2022, 30(2): 932-946.

19. Zhang J, Song B, Zeng Y, Xu C, Gao L, Guo Y, et al. m6A modification in inflammatory bowel disease provides new insights into clinical applications. Biomed Pharmacother 2023, 159: 114298.

20. Winkler R, Gillis E, Lasman L, Safra M, Geula S, Soyris C, et al. m(6)A modification controls the innate immune response to infection by targeting type I interferons. Nat Immunol 2019, 20(2): 173-182.

21. Zhang H, Liu J, Zhou Y, Qu M, Wang Y, Guo K, et al. Neutrophil extracellular traps mediate m(6)A modification and regulates sepsis-associated acute lung injury by activating ferroptosis in alveolar epithelial cells. Int J Biol Sci 2022, 18(8): 3337-3357.

22. Jiao Y, Zhang T, Zhang C, Ji H, Tong X, Xia R, et al. Exosomal miR-30d-5p of neutrophils induces M1 macrophage polarization and primes macrophage pyroptosis in sepsis-related acute lung injury. Crit Care 2021, 25(1): 356.

23. Huang X, Zhang H, Guo X, Zhu Z, Cai H, Kong X. Insulin-like growth factor 2 mRNA-binding protein 1 (IGF2BP1) in cancer. J Hematol Oncol 2018, 11(1): 88.

24. Huang H, Weng H, Chen J. m(6)A Modification in Coding and Non-coding RNAs: Roles and Therapeutic Implications in Cancer. Cancer Cell 2020, 37(3): 270-288.

25. Zhu X, Tang H, Yang M, Yin K. N6-methyladenosine in macrophage function: a novel target for metabolic diseases. Trends Endocrinol Metab 2023, 34(2): 66-84.

26. Kumari R, Ranjan P, Suleiman ZG, Goswami SK, Li J, Prasad R, et al. mRNA modifications in cardiovascular biology and disease: with a focus on m6A modification. Cardiovasc Res 2022, 118(7): 1680-1692.

27. Luo J, Xu T, Sun K. N6-Methyladenosine RNA Modification in Inflammation: Roles, Mechanisms, and Applications. Front Cell Dev Biol 2021, 9: 670711.

28. Weng H, Huang H, Wu H, Qin X, Zhao BS, Dong L, et al. METTL14 Inhibits Hematopoietic Stem/Progenitor Differentiation and Promotes Leukemogenesis via mRNA m(6)A Modification. Cell Stem Cell 2018, 22(2): 191-205 e199.

29. Zhang H, Wu D, Wang Y, Guo K, Spencer CB, Ortoga L, et al. METTL3-mediated N6-methyladenosine exacerbates ferroptosis via m6A-IGF2BP2-dependent mitochondrial metabolic reprogramming in sepsis-induced acute lung injury. Clin Transl Med 2023, 13(9): e1389.

30. Qu M, Chen Z, Qiu Z, Nan K, Wang Y, Shi Y, et al. Neutrophil extracellular traps-triggered impaired autophagic flux via METTL3 underlies sepsis-associated acute lung injury. Cell Death Discov 2022, 8(1): 375.

1. Meyer NJ, Gattinoni L, Calfee CS. Acute respiratory distress syndrome. Lancet 2021, 398(10300): 622-637.

2. Gorman EA, O'Kane CM, McAuley DF. Acute respiratory distress syndrome in adults: diagnosis, outcomes, long-term sequelae, and management. Lancet 2022, 400(10358): 1157-1170.

3. Bos LDJ, Ware LB. Acute respiratory distress syndrome: causes, pathophysiology, and phenotypes. Lancet 2022, 400(10358): 1145-1156.

4. Gilroy DW, De Maeyer RPH, Tepper M, O'Brien A, Uddin M, Chen J, et al. Treating exuberant, non-resolving inflammation in the lung; Implications for acute respiratory distress syndrome and COVID-19. Pharmacol Ther 2021, 221: 107745.

5. Laffey JG, Matthay MA. Fifty Years of Research in ARDS. Cell-based Therapy for Acute Respiratory Distress Syndrome. Biology and Potential Therapeutic Value. Am J Respir Crit Care Med 2017, 196(3): 266-273.

6. Xian H, Liu Y, Rundberg Nilsson A, Gatchalian R, Crother TR, Tourtellotte WG, et al. Metformin inhibition of mitochondrial ATP and DNA synthesis abrogates NLRP3 inflammasome activation and pulmonary inflammation. Immunity 2021, 54(7): 1463-1477 e1411.

7. Paik S, Kim JK, Silwal P, Sasakawa C, Jo EK. An update on the regulatory mechanisms of NLRP3 inflammasome activation. Cell Mol Immunol 2021, 18(5): 1141-1160.

8. Kelley N, Jeltema D, Duan Y, He Y. The NLRP3 Inflammasome: An Overview of Mechanisms of Activation and Regulation. Int J Mol Sci 2019, 20(13).

9. Swanson KV, Deng M, Ting JP. The NLRP3 inflammasome: molecular activation and regulation to therapeutics. Nat Rev Immunol 2019, 19(8): 477-489.

10. Bauernfeind FG, Horvath G, Stutz A, Alnemri ES, MacDonald K, Speert D, et al. Cutting edge: NF-kappaB activating pattern recognition and cytokine receptors license NLRP3 inflammasome activation by regulating NLRP3 expression. J Immunol 2009, 183(2): 787-791.

11. Cornut M, Bourdonnay E, Henry T. Transcriptional Regulation of Inflammasomes. Int J Mol Sci 2020, 21(21).

12. Baker PJ, De Nardo D, Moghaddas F, Tran LS, Bachem A, Nguyen T, et al. Posttranslational Modification as a Critical Determinant of Cytoplasmic Innate Immune Recognition. Physiol Rev 2017, 97(3): 1165-1209.

13. Moretti J, Blander JM. Increasing complexity of NLRP3 inflammasome regulation. J Leukoc Biol 2021, 109(3): 561-571.

14. Liu Y, Yang D, Liu T, Chen J, Yu J, Yi P. N6-methyladenosine-mediated gene regulation and therapeutic implications. Trends Mol Med 2023, 29(6): 454-467.

15. Zaccara S, Ries RJ, Jaffrey SR. Reading, writing and erasing mRNA methylation. Nat Rev Mol Cell Biol 2019, 20(10): 608-624.

16. Zhao Y, Shi Y, Shen H, Xie W. m(6)A-binding proteins: the emerging crucial performers in epigenetics. J Hematol Oncol 2020, 13(1): 35.

17. Xu H, Lin C, Yang J, Chen X, Chen Y, Chen J, et al. The Role of N(6)-Methyladenosine in Inflammatory Diseases. Oxid Med Cell Longev 2022, 2022: 9744771.

18. Peng Z, Gong Y, Wang X, He W, Wu L, Zhang L, et al. METTL3-m(6)A-Rubicon axis inhibits autophagy in nonalcoholic fatty liver disease. Mol Ther 2022, 30(2): 932-946.

19. Zhang J, Song B, Zeng Y, Xu C, Gao L, Guo Y, et al. m6A modification in inflammatory bowel disease provides new insights into clinical applications. Biomed Pharmacother 2023, 159: 114298.

20. Winkler R, Gillis E, Lasman L, Safra M, Geula S, Soyris C, et al. m(6)A modification controls the innate immune response to infection by targeting type I interferons. Nat Immunol 2019, 20(2): 173-182.

21. Zhang H, Liu J, Zhou Y, Qu M, Wang Y, Guo K, et al. Neutrophil extracellular traps mediate m(6)A modification and regulates sepsis-associated acute lung injury by activating ferroptosis in alveolar epithelial cells. Int J Biol Sci 2022, 18(8): 3337-3357.

22. Jiao Y, Zhang T, Zhang C, Ji H, Tong X, Xia R, et al. Exosomal miR-30d-5p of neutrophils induces M1 macrophage polarization and primes macrophage pyroptosis in sepsis-related acute lung injury. Crit Care 2021, 25(1): 356.

23. Huang X, Zhang H, Guo X, Zhu Z, Cai H, Kong X. Insulin-like growth factor 2 mRNA-binding protein 1 (IGF2BP1) in cancer. J Hematol Oncol 2018, 11(1): 88.

24. Huang H, Weng H, Chen J. m(6)A Modification in Coding and Non-coding RNAs: Roles and Therapeutic Implications in Cancer. Cancer Cell 2020, 37(3): 270-288.

25. Zhu X, Tang H, Yang M, Yin K. N6-methyladenosine in macrophage function: a novel target for metabolic diseases. Trends Endocrinol Metab 2023, 34(2): 66-84.

26. Kumari R, Ranjan P, Suleiman ZG, Goswami SK, Li J, Prasad R, et al. mRNA modifications in cardiovascular biology and disease: with a focus on m6A modification. Cardiovasc Res 2022, 118(7): 1680-1692.

27. Luo J, Xu T, Sun K. N6-Methyladenosine RNA Modification in Inflammation: Roles, Mechanisms, and Applications. Front Cell Dev Biol 2021, 9: 670711.

28. Weng H, Huang H, Wu H, Qin X, Zhao BS, Dong L, et al. METTL14 Inhibits Hematopoietic Stem/Progenitor Differentiation and Promotes Leukemogenesis via mRNA m(6)A Modification. Cell Stem Cell 2018, 22(2): 191-205 e199.

29. Zhang H, Wu D, Wang Y, Guo K, Spencer CB, Ortoga L, et al. METTL3-mediated N6-methyladenosine exacerbates ferroptosis via m6A-IGF2BP2-dependent mitochondrial metabolic reprogramming in sepsis-induced acute lung injury. Clin Transl Med 2023, 13(9): e1389.

31. Chen Y, Wu Y, Zhu L, Chen C, Xu S, Tang D, et al. METTL3-Mediated N6-Methyladenosine Modification of Trim59 mRNA Protects Against Sepsis-Induced Acute Respiratory Distress Syndrome. Front Immunol 2022, 13: 897487.

1. Meyer NJ, Gattinoni L, Calfee CS. Acute respiratory distress syndrome. Lancet 2021, 398(10300): 622-637.

2. Gorman EA, O'Kane CM, McAuley DF. Acute respiratory distress syndrome in adults: diagnosis, outcomes, long-term sequelae, and management. Lancet 2022, 400(10358): 1157-1170.

3. Bos LDJ, Ware LB. Acute respiratory distress syndrome: causes, pathophysiology, and phenotypes. Lancet 2022, 400(10358): 1145-1156.

4. Gilroy DW, De Maeyer RPH, Tepper M, O'Brien A, Uddin M, Chen J, et al. Treating exuberant, non-resolving inflammation in the lung; Implications for acute respiratory distress syndrome and COVID-19. Pharmacol Ther 2021, 221: 107745.

5. Laffey JG, Matthay MA. Fifty Years of Research in ARDS. Cell-based Therapy for Acute Respiratory Distress Syndrome. Biology and Potential Therapeutic Value. Am J Respir Crit Care Med 2017, 196(3): 266-273.

6. Xian H, Liu Y, Rundberg Nilsson A, Gatchalian R, Crother TR, Tourtellotte WG, et al. Metformin inhibition of mitochondrial ATP and DNA synthesis abrogates NLRP3 inflammasome activation and pulmonary inflammation. Immunity 2021, 54(7): 1463-1477 e1411.

7. Paik S, Kim JK, Silwal P, Sasakawa C, Jo EK. An update on the regulatory mechanisms of NLRP3 inflammasome activation. Cell Mol Immunol 2021, 18(5): 1141-1160.

8. Kelley N, Jeltema D, Duan Y, He Y. The NLRP3 Inflammasome: An Overview of Mechanisms of Activation and Regulation. Int J Mol Sci 2019, 20(13).

9. Swanson KV, Deng M, Ting JP. The NLRP3 inflammasome: molecular activation and regulation to therapeutics. Nat Rev Immunol 2019, 19(8): 477-489.

10. Bauernfeind FG, Horvath G, Stutz A, Alnemri ES, MacDonald K, Speert D, et al. Cutting edge: NF-kappaB activating pattern recognition and cytokine receptors license NLRP3 inflammasome activation by regulating NLRP3 expression. J Immunol 2009, 183(2): 787-791.

11. Cornut M, Bourdonnay E, Henry T. Transcriptional Regulation of Inflammasomes. Int J Mol Sci 2020, 21(21).

12. Baker PJ, De Nardo D, Moghaddas F, Tran LS, Bachem A, Nguyen T, et al. Posttranslational Modification as a Critical Determinant of Cytoplasmic Innate Immune Recognition. Physiol Rev 2017, 97(3): 1165-1209.

13. Moretti J, Blander JM. Increasing complexity of NLRP3 inflammasome regulation. J Leukoc Biol 2021, 109(3): 561-571.

14. Liu Y, Yang D, Liu T, Chen J, Yu J, Yi P. N6-methyladenosine-mediated gene regulation and therapeutic implications. Trends Mol Med 2023, 29(6): 454-467.

15. Zaccara S, Ries RJ, Jaffrey SR. Reading, writing and erasing mRNA methylation. Nat Rev Mol Cell Biol 2019, 20(10): 608-624.

16. Zhao Y, Shi Y, Shen H, Xie W. m(6)A-binding proteins: the emerging crucial performers in epigenetics. J Hematol Oncol 2020, 13(1): 35.

17. Xu H, Lin C, Yang J, Chen X, Chen Y, Chen J, et al. The Role of N(6)-Methyladenosine in Inflammatory Diseases. Oxid Med Cell Longev 2022, 2022: 9744771.

18. Peng Z, Gong Y, Wang X, He W, Wu L, Zhang L, et al. METTL3-m(6)A-Rubicon axis inhibits autophagy in nonalcoholic fatty liver disease. Mol Ther 2022, 30(2): 932-946.

19. Zhang J, Song B, Zeng Y, Xu C, Gao L, Guo Y, et al. m6A modification in inflammatory bowel disease provides new insights into clinical applications. Biomed Pharmacother 2023, 159: 114298.

20. Winkler R, Gillis E, Lasman L, Safra M, Geula S, Soyris C, et al. m(6)A modification controls the innate immune response to infection by targeting type I interferons. Nat Immunol 2019, 20(2): 173-182.

21. Zhang H, Liu J, Zhou Y, Qu M, Wang Y, Guo K, et al. Neutrophil extracellular traps mediate m(6)A modification and regulates sepsis-associated acute lung injury by activating ferroptosis in alveolar epithelial cells. Int J Biol Sci 2022, 18(8): 3337-3357.

22. Jiao Y, Zhang T, Zhang C, Ji H, Tong X, Xia R, et al. Exosomal miR-30d-5p of neutrophils induces M1 macrophage polarization and primes macrophage pyroptosis in sepsis-related acute lung injury. Crit Care 2021, 25(1): 356.

23. Huang X, Zhang H, Guo X, Zhu Z, Cai H, Kong X. Insulin-like growth factor 2 mRNA-binding protein 1 (IGF2BP1) in cancer. J Hematol Oncol 2018, 11(1): 88.

24. Huang H, Weng H, Chen J. m(6)A Modification in Coding and Non-coding RNAs: Roles and Therapeutic Implications in Cancer. Cancer Cell 2020, 37(3): 270-288.

25. Zhu X, Tang H, Yang M, Yin K. N6-methyladenosine in macrophage function: a novel target for metabolic diseases. Trends Endocrinol Metab 2023, 34(2): 66-84.

26. Kumari R, Ranjan P, Suleiman ZG, Goswami SK, Li J, Prasad R, et al. mRNA modifications in cardiovascular biology and disease: with a focus on m6A modification. Cardiovasc Res 2022, 118(7): 1680-1692.

28. Weng H, Huang H, Wu H, Qin X, Zhao BS, Dong L, et al. METTL14 Inhibits Hematopoietic Stem/Progenitor Differentiation and Promotes Leukemogenesis via mRNA m(6)A Modification. Cell Stem Cell 2018, 22(2): 191-205 e199.

29. Zhang H, Wu D, Wang Y, Guo K, Spencer CB, Ortoga L, et al. METTL3-mediated N6-methyladenosine exacerbates ferroptosis via m6A-IGF2BP2-dependent mitochondrial metabolic reprogramming in sepsis-induced acute lung injury. Clin Transl Med 2023, 13(9): e1389.

30. Qu M, Chen Z, Qiu Z, Nan K, Wang Y, Shi Y, et al. Neutrophil extracellular traps-triggered impaired autophagic flux via METTL3 underlies sepsis-associated acute lung injury. Cell Death Discov 2022, 8(1): 375.

31. Chen Y, Wu Y, Zhu L, Chen C, Xu S, Tang D, et al. METTL3-Mediated N6-Methyladenosine Modification of Trim59 mRNA Protects Against Sepsis-Induced Acute Respiratory Distress Syndrome. Front Immunol 2022, 13: 897487.

32. Chen X, Tang J, Shuai W, Meng J, Feng J, Han Z. Macrophage polarization and its role in the pathogenesis of acute lung injury/acute respiratory distress syndrome. Inflamm Res 2020, 69(9): 883-895.

1. Meyer NJ, Gattinoni L, Calfee CS. Acute respiratory distress syndrome. Lancet 2021, 398(10300): 622-637.

3. Bos LDJ, Ware LB. Acute respiratory distress syndrome: causes, pathophysiology, and phenotypes. Lancet 2022, 400(10358): 1145-1156.

4. Gilroy DW, De Maeyer RPH, Tepper M, O'Brien A, Uddin M, Chen J, et al. Treating exuberant, non-resolving inflammation in the lung; Implications for acute respiratory distress syndrome and COVID-19. Pharmacol Ther 2021, 221: 107745.

5. Laffey JG, Matthay MA. Fifty Years of Research in ARDS. Cell-based Therapy for Acute Respiratory Distress Syndrome. Biology and Potential Therapeutic Value. Am J Respir Crit Care Med 2017, 196(3): 266-273.

6. Xian H, Liu Y, Rundberg Nilsson A, Gatchalian R, Crother TR, Tourtellotte WG, et al. Metformin inhibition of mitochondrial ATP and DNA synthesis abrogates NLRP3 inflammasome activation and pulmonary inflammation. Immunity 2021, 54(7): 1463-1477 e1411.

7. Paik S, Kim JK, Silwal P, Sasakawa C, Jo EK. An update on the regulatory mechanisms of NLRP3 inflammasome activation. Cell Mol Immunol 2021, 18(5): 1141-1160.

8. Kelley N, Jeltema D, Duan Y, He Y. The NLRP3 Inflammasome: An Overview of Mechanisms of Activation and Regulation. Int J Mol Sci 2019, 20(13).

9. Swanson KV, Deng M, Ting JP. The NLRP3 inflammasome: molecular activation and regulation to therapeutics. Nat Rev Immunol 2019, 19(8): 477-489.

10. Bauernfeind FG, Horvath G, Stutz A, Alnemri ES, MacDonald K, Speert D, et al. Cutting edge: NF-kappaB activating pattern recognition and cytokine receptors license NLRP3 inflammasome activation by regulating NLRP3 expression. J Immunol 2009, 183(2): 787-791.

11. Cornut M, Bourdonnay E, Henry T. Transcriptional Regulation of Inflammasomes. Int J Mol Sci 2020, 21(21).

12. Baker PJ, De Nardo D, Moghaddas F, Tran LS, Bachem A, Nguyen T, et al. Posttranslational Modification as a Critical Determinant of Cytoplasmic Innate Immune Recognition. Physiol Rev 2017, 97(3): 1165-1209.

13. Moretti J, Blander JM. Increasing complexity of NLRP3 inflammasome regulation. J Leukoc Biol 2021, 109(3): 561-571.

14. Liu Y, Yang D, Liu T, Chen J, Yu J, Yi P. N6-methyladenosine-mediated gene regulation and therapeutic implications. Trends Mol Med 2023, 29(6): 454-467.

15. Zaccara S, Ries RJ, Jaffrey SR. Reading, writing and erasing mRNA methylation. Nat Rev Mol Cell Biol 2019, 20(10): 608-624.

16. Zhao Y, Shi Y, Shen H, Xie W. m(6)A-binding proteins: the emerging crucial performers in epigenetics. J Hematol Oncol 2020, 13(1): 35.

17. Xu H, Lin C, Yang J, Chen X, Chen Y, Chen J, et al. The Role of N(6)-Methyladenosine in Inflammatory Diseases. Oxid Med Cell Longev 2022, 2022: 9744771.

18. Peng Z, Gong Y, Wang X, He W, Wu L, Zhang L, et al. METTL3-m(6)A-Rubicon axis inhibits autophagy in nonalcoholic fatty liver disease. Mol Ther 2022, 30(2): 932-946.

19. Zhang J, Song B, Zeng Y, Xu C, Gao L, Guo Y, et al. m6A modification in inflammatory bowel disease provides new insights into clinical applications. Biomed Pharmacother 2023, 159: 114298.

20. Winkler R, Gillis E, Lasman L, Safra M, Geula S, Soyris C, et al. m(6)A modification controls the innate immune response to infection by targeting type I interferons. Nat Immunol 2019, 20(2): 173-182.

21. Zhang H, Liu J, Zhou Y, Qu M, Wang Y, Guo K, et al. Neutrophil extracellular traps mediate m(6)A modification and regulates sepsis-associated acute lung injury by activating ferroptosis in alveolar epithelial cells. Int J Biol Sci 2022, 18(8): 3337-3357.

22. Jiao Y, Zhang T, Zhang C, Ji H, Tong X, Xia R, et al. Exosomal miR-30d-5p of neutrophils induces M1 macrophage polarization and primes macrophage pyroptosis in sepsis-related acute lung injury. Crit Care 2021, 25(1): 356.

23. Huang X, Zhang H, Guo X, Zhu Z, Cai H, Kong X. Insulin-like growth factor 2 mRNA-binding protein 1 (IGF2BP1) in cancer. J Hematol Oncol 2018, 11(1): 88.

24. Huang H, Weng H, Chen J. m(6)A Modification in Coding and Non-coding RNAs: Roles and Therapeutic Implications in Cancer. Cancer Cell 2020, 37(3): 270-288.

25. Zhu X, Tang H, Yang M, Yin K. N6-methyladenosine in macrophage function: a novel target for metabolic diseases. Trends Endocrinol Metab 2023, 34(2): 66-84.

26. Kumari R, Ranjan P, Suleiman ZG, Goswami SK, Li J, Prasad R, et al. mRNA modifications in cardiovascular biology and disease: with a focus on m6A modification. Cardiovasc Res 2022, 118(7): 1680-1692.

27. Luo J, Xu T, Sun K. N6-Methyladenosine RNA Modification in Inflammation: Roles, Mechanisms, and Applications. Front Cell Dev Biol 2021, 9: 670711.

28. Weng H, Huang H, Wu H, Qin X, Zhao BS, Dong L, et al. METTL14 Inhibits Hematopoietic Stem/Progenitor Differentiation and Promotes Leukemogenesis via mRNA m(6)A Modification. Cell Stem Cell 2018, 22(2): 191-205 e199.

29. Zhang H, Wu D, Wang Y, Guo K, Spencer CB, Ortoga L, et al. METTL3-mediated N6-methyladenosine exacerbates ferroptosis via m6A-IGF2BP2-dependent mitochondrial metabolic reprogramming in sepsis-induced acute lung injury. Clin Transl Med 2023, 13(9): e1389.

30. Qu M, Chen Z, Qiu Z, Nan K, Wang Y, Shi Y, et al. Neutrophil extracellular traps-triggered impaired autophagic flux via METTL3 underlies sepsis-associated acute lung injury. Cell Death Discov 2022, 8(1): 375.

31. Chen Y, Wu Y, Zhu L, Chen C, Xu S, Tang D, et al. METTL3-Mediated N6-Methyladenosine Modification of Trim59 mRNA Protects Against Sepsis-Induced Acute Respiratory Distress Syndrome. Front Immunol 2022, 13: 897487.

32. Chen X, Tang J, Shuai W, Meng J, Feng J, Han Z. Macrophage polarization and its role in the pathogenesis of acute lung injury/acute respiratory distress syndrome. Inflamm Res 2020, 69(9): 883-895.

33. Zaslona Z, Przybranowski S, Wilke C, van Rooijen N, Teitz-Tennenbaum S, Osterholzer JJ, et al. Resident alveolar macrophages suppress, whereas recruited monocytes promote, allergic lung inflammation in murine models of asthma. J Immunol 2014, 193(8): 4245-4253.

1. Meyer NJ, Gattinoni L, Calfee CS. Acute respiratory distress syndrome. Lancet 2021, 398(10300): 622-637.

2. Gorman EA, O'Kane CM, McAuley DF. Acute respiratory distress syndrome in adults: diagnosis, outcomes, long-term sequelae, and management. Lancet 2022, 400(10358): 1157-1170.

3. Bos LDJ, Ware LB. Acute respiratory distress syndrome: causes, pathophysiology, and phenotypes. Lancet 2022, 400(10358): 1145-1156.

4. Gilroy DW, De Maeyer RPH, Tepper M, O'Brien A, Uddin M, Chen J, et al. Treating exuberant, non-resolving inflammation in the lung; Implications for acute respiratory distress syndrome and COVID-19. Pharmacol Ther 2021, 221: 107745.

5. Laffey JG, Matthay MA. Fifty Years of Research in ARDS. Cell-based Therapy for Acute Respiratory Distress Syndrome. Biology and Potential Therapeutic Value. Am J Respir Crit Care Med 2017, 196(3): 266-273.

6. Xian H, Liu Y, Rundberg Nilsson A, Gatchalian R, Crother TR, Tourtellotte WG, et al. Metformin inhibition of mitochondrial ATP and DNA synthesis abrogates NLRP3 inflammasome activation and pulmonary inflammation. Immunity 2021, 54(7): 1463-1477 e1411.

7. Paik S, Kim JK, Silwal P, Sasakawa C, Jo EK. An update on the regulatory mechanisms of NLRP3 inflammasome activation. Cell Mol Immunol 2021, 18(5): 1141-1160.

8. Kelley N, Jeltema D, Duan Y, He Y. The NLRP3 Inflammasome: An Overview of Mechanisms of Activation and Regulation. Int J Mol Sci 2019, 20(13).

9. Swanson KV, Deng M, Ting JP. The NLRP3 inflammasome: molecular activation and regulation to therapeutics. Nat Rev Immunol 2019, 19(8): 477-489.

10. Bauernfeind FG, Horvath G, Stutz A, Alnemri ES, MacDonald K, Speert D, et al. Cutting edge: NF-kappaB activating pattern recognition and cytokine receptors license NLRP3 inflammasome activation by regulating NLRP3 expression. J Immunol 2009, 183(2): 787-791.

11. Cornut M, Bourdonnay E, Henry T. Transcriptional Regulation of Inflammasomes. Int J Mol Sci 2020, 21(21).

12. Baker PJ, De Nardo D, Moghaddas F, Tran LS, Bachem A, Nguyen T, et al. Posttranslational Modification as a Critical Determinant of Cytoplasmic Innate Immune Recognition. Physiol Rev 2017, 97(3): 1165-1209.

13. Moretti J, Blander JM. Increasing complexity of NLRP3 inflammasome regulation. J Leukoc Biol 2021, 109(3): 561-571.

14. Liu Y, Yang D, Liu T, Chen J, Yu J, Yi P. N6-methyladenosine-mediated gene regulation and therapeutic implications. Trends Mol Med 2023, 29(6): 454-467.

15. Zaccara S, Ries RJ, Jaffrey SR. Reading, writing and erasing mRNA methylation. Nat Rev Mol Cell Biol 2019, 20(10): 608-624.

16. Zhao Y, Shi Y, Shen H, Xie W. m(6)A-binding proteins: the emerging crucial performers in epigenetics. J Hematol Oncol 2020, 13(1): 35.

17. Xu H, Lin C, Yang J, Chen X, Chen Y, Chen J, et al. The Role of N(6)-Methyladenosine in Inflammatory Diseases. Oxid Med Cell Longev 2022, 2022: 9744771.

18. Peng Z, Gong Y, Wang X, He W, Wu L, Zhang L, et al. METTL3-m(6)A-Rubicon axis inhibits autophagy in nonalcoholic fatty liver disease. Mol Ther 2022, 30(2): 932-946.

19. Zhang J, Song B, Zeng Y, Xu C, Gao L, Guo Y, et al. m6A modification in inflammatory bowel disease provides new insights into clinical applications. Biomed Pharmacother 2023, 159: 114298.

20. Winkler R, Gillis E, Lasman L, Safra M, Geula S, Soyris C, et al. m(6)A modification controls the innate immune response to infection by targeting type I interferons. Nat Immunol 2019, 20(2): 173-182.

21. Zhang H, Liu J, Zhou Y, Qu M, Wang Y, Guo K, et al. Neutrophil extracellular traps mediate m(6)A modification and regulates sepsis-associated acute lung injury by activating ferroptosis in alveolar epithelial cells. Int J Biol Sci 2022, 18(8): 3337-3357.

22. Jiao Y, Zhang T, Zhang C, Ji H, Tong X, Xia R, et al. Exosomal miR-30d-5p of neutrophils induces M1 macrophage polarization and primes macrophage pyroptosis in sepsis-related acute lung injury. Crit Care 2021, 25(1): 356.

23. Huang X, Zhang H, Guo X, Zhu Z, Cai H, Kong X. Insulin-like growth factor 2 mRNA-binding protein 1 (IGF2BP1) in cancer. J Hematol Oncol 2018, 11(1): 88.

24. Huang H, Weng H, Chen J. m(6)A Modification in Coding and Non-coding RNAs: Roles and Therapeutic Implications in Cancer. Cancer Cell 2020, 37(3): 270-288.

25. Zhu X, Tang H, Yang M, Yin K. N6-methyladenosine in macrophage function: a novel target for metabolic diseases. Trends Endocrinol Metab 2023, 34(2): 66-84.

26. Kumari R, Ranjan P, Suleiman ZG, Goswami SK, Li J, Prasad R, et al. mRNA modifications in cardiovascular biology and disease: with a focus on m6A modification. Cardiovasc Res 2022, 118(7): 1680-1692.

27. Luo J, Xu T, Sun K. N6-Methyladenosine RNA Modification in Inflammation: Roles, Mechanisms, and Applications. Front Cell Dev Biol 2021, 9: 670711.

28. Weng H, Huang H, Wu H, Qin X, Zhao BS, Dong L, et al. METTL14 Inhibits Hematopoietic Stem/Progenitor Differentiation and Promotes Leukemogenesis via mRNA m(6)A Modification. Cell Stem Cell 2018, 22(2): 191-205 e199.

30. Qu M, Chen Z, Qiu Z, Nan K, Wang Y, Shi Y, et al. Neutrophil extracellular traps-triggered impaired autophagic flux via METTL3 underlies sepsis-associated acute lung injury. Cell Death Discov 2022, 8(1): 375.

31. Chen Y, Wu Y, Zhu L, Chen C, Xu S, Tang D, et al. METTL3-Mediated N6-Methyladenosine Modification of Trim59 mRNA Protects Against Sepsis-Induced Acute Respiratory Distress Syndrome. Front Immunol 2022, 13: 897487.

32. Chen X, Tang J, Shuai W, Meng J, Feng J, Han Z. Macrophage polarization and its role in the pathogenesis of acute lung injury/acute respiratory distress syndrome. Inflamm Res 2020, 69(9): 883-895.

33. Zaslona Z, Przybranowski S, Wilke C, van Rooijen N, Teitz-Tennenbaum S, Osterholzer JJ, et al. Resident alveolar macrophages suppress, whereas recruited monocytes promote, allergic lung inflammation in murine models of asthma. J Immunol 2014, 193(8): 4245-4253.

34. Han W, Tanjore H, Liu Y, Hunt RP, Gutor SS, Serezani APM, et al. Identification and Characterization of Alveolar and Recruited Lung Macrophages during Acute Lung Inflammation. J Immunol 2023, 210(11): 1827-1836.

1. Meyer NJ, Gattinoni L, Calfee CS. Acute respiratory distress syndrome. Lancet 2021, 398(10300): 622-637.

2. Gorman EA, O'Kane CM, McAuley DF. Acute respiratory distress syndrome in adults: diagnosis, outcomes, long-term sequelae, and management. Lancet 2022, 400(10358): 1157-1170.

3. Bos LDJ, Ware LB. Acute respiratory distress syndrome: causes, pathophysiology, and phenotypes. Lancet 2022, 400(10358): 1145-1156.

4. Gilroy DW, De Maeyer RPH, Tepper M, O'Brien A, Uddin M, Chen J, et al. Treating exuberant, non-resolving inflammation in the lung; Implications for acute respiratory distress syndrome and COVID-19. Pharmacol Ther 2021, 221: 107745.

5. Laffey JG, Matthay MA. Fifty Years of Research in ARDS. Cell-based Therapy for Acute Respiratory Distress Syndrome. Biology and Potential Therapeutic Value. Am J Respir Crit Care Med 2017, 196(3): 266-273.

6. Xian H, Liu Y, Rundberg Nilsson A, Gatchalian R, Crother TR, Tourtellotte WG, et al. Metformin inhibition of mitochondrial ATP and DNA synthesis abrogates NLRP3 inflammasome activation and pulmonary inflammation. Immunity 2021, 54(7): 1463-1477 e1411.

7. Paik S, Kim JK, Silwal P, Sasakawa C, Jo EK. An update on the regulatory mechanisms of NLRP3 inflammasome activation. Cell Mol Immunol 2021, 18(5): 1141-1160.

8. Kelley N, Jeltema D, Duan Y, He Y. The NLRP3 Inflammasome: An Overview of Mechanisms of Activation and Regulation. Int J Mol Sci 2019, 20(13).

9. Swanson KV, Deng M, Ting JP. The NLRP3 inflammasome: molecular activation and regulation to therapeutics. Nat Rev Immunol 2019, 19(8): 477-489.

10. Bauernfeind FG, Horvath G, Stutz A, Alnemri ES, MacDonald K, Speert D, et al. Cutting edge: NF-kappaB activating pattern recognition and cytokine receptors license NLRP3 inflammasome activation by regulating NLRP3 expression. J Immunol 2009, 183(2): 787-791.

11. Cornut M, Bourdonnay E, Henry T. Transcriptional Regulation of Inflammasomes. Int J Mol Sci 2020, 21(21).

12. Baker PJ, De Nardo D, Moghaddas F, Tran LS, Bachem A, Nguyen T, et al. Posttranslational Modification as a Critical Determinant of Cytoplasmic Innate Immune Recognition. Physiol Rev 2017, 97(3): 1165-1209.

13. Moretti J, Blander JM. Increasing complexity of NLRP3 inflammasome regulation. J Leukoc Biol 2021, 109(3): 561-571.

14. Liu Y, Yang D, Liu T, Chen J, Yu J, Yi P. N6-methyladenosine-mediated gene regulation and therapeutic implications. Trends Mol Med 2023, 29(6): 454-467.

15. Zaccara S, Ries RJ, Jaffrey SR. Reading, writing and erasing mRNA methylation. Nat Rev Mol Cell Biol 2019, 20(10): 608-624.

16. Zhao Y, Shi Y, Shen H, Xie W. m(6)A-binding proteins: the emerging crucial performers in epigenetics. J Hematol Oncol 2020, 13(1): 35.

17. Xu H, Lin C, Yang J, Chen X, Chen Y, Chen J, et al. The Role of N(6)-Methyladenosine in Inflammatory Diseases. Oxid Med Cell Longev 2022, 2022: 9744771.

18. Peng Z, Gong Y, Wang X, He W, Wu L, Zhang L, et al. METTL3-m(6)A-Rubicon axis inhibits autophagy in nonalcoholic fatty liver disease. Mol Ther 2022, 30(2): 932-946.

20. Winkler R, Gillis E, Lasman L, Safra M, Geula S, Soyris C, et al. m(6)A modification controls the innate immune response to infection by targeting type I interferons. Nat Immunol 2019, 20(2): 173-182.

21. Zhang H, Liu J, Zhou Y, Qu M, Wang Y, Guo K, et al. Neutrophil extracellular traps mediate m(6)A modification and regulates sepsis-associated acute lung injury by activating ferroptosis in alveolar epithelial cells. Int J Biol Sci 2022, 18(8): 3337-3357.

22. Jiao Y, Zhang T, Zhang C, Ji H, Tong X, Xia R, et al. Exosomal miR-30d-5p of neutrophils induces M1 macrophage polarization and primes macrophage pyroptosis in sepsis-related acute lung injury. Crit Care 2021, 25(1): 356.

23. Huang X, Zhang H, Guo X, Zhu Z, Cai H, Kong X. Insulin-like growth factor 2 mRNA-binding protein 1 (IGF2BP1) in cancer. J Hematol Oncol 2018, 11(1): 88.

24. Huang H, Weng H, Chen J. m(6)A Modification in Coding and Non-coding RNAs: Roles and Therapeutic Implications in Cancer. Cancer Cell 2020, 37(3): 270-288.

25. Zhu X, Tang H, Yang M, Yin K. N6-methyladenosine in macrophage function: a novel target for metabolic diseases. Trends Endocrinol Metab 2023, 34(2): 66-84.

26. Kumari R, Ranjan P, Suleiman ZG, Goswami SK, Li J, Prasad R, et al. mRNA modifications in cardiovascular biology and disease: with a focus on m6A modification. Cardiovasc Res 2022, 118(7): 1680-1692.

27. Luo J, Xu T, Sun K. N6-Methyladenosine RNA Modification in Inflammation: Roles, Mechanisms, and Applications. Front Cell Dev Biol 2021, 9: 670711.

28. Weng H, Huang H, Wu H, Qin X, Zhao BS, Dong L, et al. METTL14 Inhibits Hematopoietic Stem/Progenitor Differentiation and Promotes Leukemogenesis via mRNA m(6)A Modification. Cell Stem Cell 2018, 22(2): 191-205 e199.

29. Zhang H, Wu D, Wang Y, Guo K, Spencer CB, Ortoga L, et al. METTL3-mediated N6-methyladenosine exacerbates ferroptosis via m6A-IGF2BP2-dependent mitochondrial metabolic reprogramming in sepsis-induced acute lung injury. Clin Transl Med 2023, 13(9): e1389.

30. Qu M, Chen Z, Qiu Z, Nan K, Wang Y, Shi Y, et al. Neutrophil extracellular traps-triggered impaired autophagic flux via METTL3 underlies sepsis-associated acute lung injury. Cell Death Discov 2022, 8(1): 375.

31. Chen Y, Wu Y, Zhu L, Chen C, Xu S, Tang D, et al. METTL3-Mediated N6-Methyladenosine Modification of Trim59 mRNA Protects Against Sepsis-Induced Acute Respiratory Distress Syndrome. Front Immunol 2022, 13: 897487.

32. Chen X, Tang J, Shuai W, Meng J, Feng J, Han Z. Macrophage polarization and its role in the pathogenesis of acute lung injury/acute respiratory distress syndrome. Inflamm Res 2020, 69(9): 883-895.

33. Zaslona Z, Przybranowski S, Wilke C, van Rooijen N, Teitz-Tennenbaum S, Osterholzer JJ, et al. Resident alveolar macrophages suppress, whereas recruited monocytes promote, allergic lung inflammation in murine models of asthma. J Immunol 2014, 193(8): 4245-4253.

34. Han W, Tanjore H, Liu Y, Hunt RP, Gutor SS, Serezani APM, et al. Identification and Characterization of Alveolar and Recruited Lung Macrophages during Acute Lung Inflammation. J Immunol 2023, 210(11): 1827-1836.

35. Li Y, Li J, Yu Q, Ji L, Peng B. METTL14 regulates microglia/macrophage polarization and NLRP3 inflammasome activation after ischemic stroke by the KAT3B-STING axis. Neurobiol Dis 2023, 185: 106253.

1. Meyer NJ, Gattinoni L, Calfee CS. Acute respiratory distress syndrome. Lancet 2021, 398(10300): 622-637.

2. Gorman EA, O'Kane CM, McAuley DF. Acute respiratory distress syndrome in adults: diagnosis, outcomes, long-term sequelae, and management. Lancet 2022, 400(10358): 1157-1170.

3. Bos LDJ, Ware LB. Acute respiratory distress syndrome: causes, pathophysiology, and phenotypes. Lancet 2022, 400(10358): 1145-1156.

4. Gilroy DW, De Maeyer RPH, Tepper M, O'Brien A, Uddin M, Chen J, et al. Treating exuberant, non-resolving inflammation in the lung; Implications for acute respiratory distress syndrome and COVID-19. Pharmacol Ther 2021, 221: 107745.

6. Xian H, Liu Y, Rundberg Nilsson A, Gatchalian R, Crother TR, Tourtellotte WG, et al. Metformin inhibition of mitochondrial ATP and DNA synthesis abrogates NLRP3 inflammasome activation and pulmonary inflammation. Immunity 2021, 54(7): 1463-1477 e1411.

7. Paik S, Kim JK, Silwal P, Sasakawa C, Jo EK. An update on the regulatory mechanisms of NLRP3 inflammasome activation. Cell Mol Immunol 2021, 18(5): 1141-1160.

8. Kelley N, Jeltema D, Duan Y, He Y. The NLRP3 Inflammasome: An Overview of Mechanisms of Activation and Regulation. Int J Mol Sci 2019, 20(13).

9. Swanson KV, Deng M, Ting JP. The NLRP3 inflammasome: molecular activation and regulation to therapeutics. Nat Rev Immunol 2019, 19(8): 477-489.

10. Bauernfeind FG, Horvath G, Stutz A, Alnemri ES, MacDonald K, Speert D, et al. Cutting edge: NF-kappaB activating pattern recognition and cytokine receptors license NLRP3 inflammasome activation by regulating NLRP3 expression. J Immunol 2009, 183(2): 787-791.

11. Cornut M, Bourdonnay E, Henry T. Transcriptional Regulation of Inflammasomes. Int J Mol Sci 2020, 21(21).

12. Baker PJ, De Nardo D, Moghaddas F, Tran LS, Bachem A, Nguyen T, et al. Posttranslational Modification as a Critical Determinant of Cytoplasmic Innate Immune Recognition. Physiol Rev 2017, 97(3): 1165-1209.

13. Moretti J, Blander JM. Increasing complexity of NLRP3 inflammasome regulation. J Leukoc Biol 2021, 109(3): 561-571.

14. Liu Y, Yang D, Liu T, Chen J, Yu J, Yi P. N6-methyladenosine-mediated gene regulation and therapeutic implications. Trends Mol Med 2023, 29(6): 454-467.

15. Zaccara S, Ries RJ, Jaffrey SR. Reading, writing and erasing mRNA methylation. Nat Rev Mol Cell Biol 2019, 20(10): 608-624.

16. Zhao Y, Shi Y, Shen H, Xie W. m(6)A-binding proteins: the emerging crucial performers in epigenetics. J Hematol Oncol 2020, 13(1): 35.

17. Xu H, Lin C, Yang J, Chen X, Chen Y, Chen J, et al. The Role of N(6)-Methyladenosine in Inflammatory Diseases. Oxid Med Cell Longev 2022, 2022: 9744771.

18. Peng Z, Gong Y, Wang X, He W, Wu L, Zhang L, et al. METTL3-m(6)A-Rubicon axis inhibits autophagy in nonalcoholic fatty liver disease. Mol Ther 2022, 30(2): 932-946.

19. Zhang J, Song B, Zeng Y, Xu C, Gao L, Guo Y, et al. m6A modification in inflammatory bowel disease provides new insights into clinical applications. Biomed Pharmacother 2023, 159: 114298.

20. Winkler R, Gillis E, Lasman L, Safra M, Geula S, Soyris C, et al. m(6)A modification controls the innate immune response to infection by targeting type I interferons. Nat Immunol 2019, 20(2): 173-182.

21. Zhang H, Liu J, Zhou Y, Qu M, Wang Y, Guo K, et al. Neutrophil extracellular traps mediate m(6)A modification and regulates sepsis-associated acute lung injury by activating ferroptosis in alveolar epithelial cells. Int J Biol Sci 2022, 18(8): 3337-3357.

22. Jiao Y, Zhang T, Zhang C, Ji H, Tong X, Xia R, et al. Exosomal miR-30d-5p of neutrophils induces M1 macrophage polarization and primes macrophage pyroptosis in sepsis-related acute lung injury. Crit Care 2021, 25(1): 356.

23. Huang X, Zhang H, Guo X, Zhu Z, Cai H, Kong X. Insulin-like growth factor 2 mRNA-binding protein 1 (IGF2BP1) in cancer. J Hematol Oncol 2018, 11(1): 88.

24. Huang H, Weng H, Chen J. m(6)A Modification in Coding and Non-coding RNAs: Roles and Therapeutic Implications in Cancer. Cancer Cell 2020, 37(3): 270-288.

25. Zhu X, Tang H, Yang M, Yin K. N6-methyladenosine in macrophage function: a novel target for metabolic diseases. Trends Endocrinol Metab 2023, 34(2): 66-84.

26. Kumari R, Ranjan P, Suleiman ZG, Goswami SK, Li J, Prasad R, et al. mRNA modifications in cardiovascular biology and disease: with a focus on m6A modification. Cardiovasc Res 2022, 118(7): 1680-1692.

27. Luo J, Xu T, Sun K. N6-Methyladenosine RNA Modification in Inflammation: Roles, Mechanisms, and Applications. Front Cell Dev Biol 2021, 9: 670711.

28. Weng H, Huang H, Wu H, Qin X, Zhao BS, Dong L, et al. METTL14 Inhibits Hematopoietic Stem/Progenitor Differentiation and Promotes Leukemogenesis via mRNA m(6)A Modification. Cell Stem Cell 2018, 22(2): 191-205 e199.

29. Zhang H, Wu D, Wang Y, Guo K, Spencer CB, Ortoga L, et al. METTL3-mediated N6-methyladenosine exacerbates ferroptosis via m6A-IGF2BP2-dependent mitochondrial metabolic reprogramming in sepsis-induced acute lung injury. Clin Transl Med 2023, 13(9): e1389.

30. Qu M, Chen Z, Qiu Z, Nan K, Wang Y, Shi Y, et al. Neutrophil extracellular traps-triggered impaired autophagic flux via METTL3 underlies sepsis-associated acute lung injury. Cell Death Discov 2022, 8(1): 375.

31. Chen Y, Wu Y, Zhu L, Chen C, Xu S, Tang D, et al. METTL3-Mediated N6-Methyladenosine Modification of Trim59 mRNA Protects Against Sepsis-Induced Acute Respiratory Distress Syndrome. Front Immunol 2022, 13: 897487.

32. Chen X, Tang J, Shuai W, Meng J, Feng J, Han Z. Macrophage polarization and its role in the pathogenesis of acute lung injury/acute respiratory distress syndrome. Inflamm Res 2020, 69(9): 883-895.

33. Zaslona Z, Przybranowski S, Wilke C, van Rooijen N, Teitz-Tennenbaum S, Osterholzer JJ, et al. Resident alveolar macrophages suppress, whereas recruited monocytes promote, allergic lung inflammation in murine models of asthma. J Immunol 2014, 193(8): 4245-4253.

34. Han W, Tanjore H, Liu Y, Hunt RP, Gutor SS, Serezani APM, et al. Identification and Characterization of Alveolar and Recruited Lung Macrophages during Acute Lung Inflammation. J Immunol 2023, 210(11): 1827-1836.

35. Li Y, Li J, Yu Q, Ji L, Peng B. METTL14 regulates microglia/macrophage polarization and NLRP3 inflammasome activation after ischemic stroke by the KAT3B-STING axis. Neurobiol Dis 2023, 185: 106253.

36. Zheng Y, Li Y, Ran X, Wang D, Zheng X, Zhang M, et al. Mettl14 mediates the inflammatory response of macrophages in atherosclerosis through the NF-kappaB/IL-6 signaling pathway. Cell Mol Life Sci 2022, 79(6): 311.

1. Meyer NJ, Gattinoni L, Calfee CS. Acute respiratory distress syndrome. Lancet 2021, 398(10300): 622-637.

2. Gorman EA, O'Kane CM, McAuley DF. Acute respiratory distress syndrome in adults: diagnosis, outcomes, long-term sequelae, and management. Lancet 2022, 400(10358): 1157-1170.

3. Bos LDJ, Ware LB. Acute respiratory distress syndrome: causes, pathophysiology, and phenotypes. Lancet 2022, 400(10358): 1145-1156.

4. Gilroy DW, De Maeyer RPH, Tepper M, O'Brien A, Uddin M, Chen J, et al. Treating exuberant, non-resolving inflammation in the lung; Implications for acute respiratory distress syndrome and COVID-19. Pharmacol Ther 2021, 221: 107745.

5. Laffey JG, Matthay MA. Fifty Years of Research in ARDS. Cell-based Therapy for Acute Respiratory Distress Syndrome. Biology and Potential Therapeutic Value. Am J Respir Crit Care Med 2017, 196(3): 266-273.

6. Xian H, Liu Y, Rundberg Nilsson A, Gatchalian R, Crother TR, Tourtellotte WG, et al. Metformin inhibition of mitochondrial ATP and DNA synthesis abrogates NLRP3 inflammasome activation and pulmonary inflammation. Immunity 2021, 54(7): 1463-1477 e1411.

7. Paik S, Kim JK, Silwal P, Sasakawa C, Jo EK. An update on the regulatory mechanisms of NLRP3 inflammasome activation. Cell Mol Immunol 2021, 18(5): 1141-1160.

8. Kelley N, Jeltema D, Duan Y, He Y. The NLRP3 Inflammasome: An Overview of Mechanisms of Activation and Regulation. Int J Mol Sci 2019, 20(13).

9. Swanson KV, Deng M, Ting JP. The NLRP3 inflammasome: molecular activation and regulation to therapeutics. Nat Rev Immunol 2019, 19(8): 477-489.

10. Bauernfeind FG, Horvath G, Stutz A, Alnemri ES, MacDonald K, Speert D, et al. Cutting edge: NF-kappaB activating pattern recognition and cytokine receptors license NLRP3 inflammasome activation by regulating NLRP3 expression. J Immunol 2009, 183(2): 787-791.

11. Cornut M, Bourdonnay E, Henry T. Transcriptional Regulation of Inflammasomes. Int J Mol Sci 2020, 21(21).

12. Baker PJ, De Nardo D, Moghaddas F, Tran LS, Bachem A, Nguyen T, et al. Posttranslational Modification as a Critical Determinant of Cytoplasmic Innate Immune Recognition. Physiol Rev 2017, 97(3): 1165-1209.

13. Moretti J, Blander JM. Increasing complexity of NLRP3 inflammasome regulation. J Leukoc Biol 2021, 109(3): 561-571.

14. Liu Y, Yang D, Liu T, Chen J, Yu J, Yi P. N6-methyladenosine-mediated gene regulation and therapeutic implications. Trends Mol Med 2023, 29(6): 454-467.

15. Zaccara S, Ries RJ, Jaffrey SR. Reading, writing and erasing mRNA methylation. Nat Rev Mol Cell Biol 2019, 20(10): 608-624.

16. Zhao Y, Shi Y, Shen H, Xie W. m(6)A-binding proteins: the emerging crucial performers in epigenetics. J Hematol Oncol 2020, 13(1): 35.

17. Xu H, Lin C, Yang J, Chen X, Chen Y, Chen J, et al. The Role of N(6)-Methyladenosine in Inflammatory Diseases. Oxid Med Cell Longev 2022, 2022: 9744771.

18. Peng Z, Gong Y, Wang X, He W, Wu L, Zhang L, et al. METTL3-m(6)A-Rubicon axis inhibits autophagy in nonalcoholic fatty liver disease. Mol Ther 2022, 30(2): 932-946.

19. Zhang J, Song B, Zeng Y, Xu C, Gao L, Guo Y, et al. m6A modification in inflammatory bowel disease provides new insights into clinical applications. Biomed Pharmacother 2023, 159: 114298.

20. Winkler R, Gillis E, Lasman L, Safra M, Geula S, Soyris C, et al. m(6)A modification controls the innate immune response to infection by targeting type I interferons. Nat Immunol 2019, 20(2): 173-182.

21. Zhang H, Liu J, Zhou Y, Qu M, Wang Y, Guo K, et al. Neutrophil extracellular traps mediate m(6)A modification and regulates sepsis-associated acute lung injury by activating ferroptosis in alveolar epithelial cells. Int J Biol Sci 2022, 18(8): 3337-3357.

22. Jiao Y, Zhang T, Zhang C, Ji H, Tong X, Xia R, et al. Exosomal miR-30d-5p of neutrophils induces M1 macrophage polarization and primes macrophage pyroptosis in sepsis-related acute lung injury. Crit Care 2021, 25(1): 356.

23. Huang X, Zhang H, Guo X, Zhu Z, Cai H, Kong X. Insulin-like growth factor 2 mRNA-binding protein 1 (IGF2BP1) in cancer. J Hematol Oncol 2018, 11(1): 88.

24. Huang H, Weng H, Chen J. m(6)A Modification in Coding and Non-coding RNAs: Roles and Therapeutic Implications in Cancer. Cancer Cell 2020, 37(3): 270-288.

25. Zhu X, Tang H, Yang M, Yin K. N6-methyladenosine in macrophage function: a novel target for metabolic diseases. Trends Endocrinol Metab 2023, 34(2): 66-84.

26. Kumari R, Ranjan P, Suleiman ZG, Goswami SK, Li J, Prasad R, et al. mRNA modifications in cardiovascular biology and disease: with a focus on m6A modification. Cardiovasc Res 2022, 118(7): 1680-1692.

27. Luo J, Xu T, Sun K. N6-Methyladenosine RNA Modification in Inflammation: Roles, Mechanisms, and Applications. Front Cell Dev Biol 2021, 9: 670711.

29. Zhang H, Wu D, Wang Y, Guo K, Spencer CB, Ortoga L, et al. METTL3-mediated N6-methyladenosine exacerbates ferroptosis via m6A-IGF2BP2-dependent mitochondrial metabolic reprogramming in sepsis-induced acute lung injury. Clin Transl Med 2023, 13(9): e1389.

30. Qu M, Chen Z, Qiu Z, Nan K, Wang Y, Shi Y, et al. Neutrophil extracellular traps-triggered impaired autophagic flux via METTL3 underlies sepsis-associated acute lung injury. Cell Death Discov 2022, 8(1): 375.

31. Chen Y, Wu Y, Zhu L, Chen C, Xu S, Tang D, et al. METTL3-Mediated N6-Methyladenosine Modification of Trim59 mRNA Protects Against Sepsis-Induced Acute Respiratory Distress Syndrome. Front Immunol 2022, 13: 897487.

32. Chen X, Tang J, Shuai W, Meng J, Feng J, Han Z. Macrophage polarization and its role in the pathogenesis of acute lung injury/acute respiratory distress syndrome. Inflamm Res 2020, 69(9): 883-895.

33. Zaslona Z, Przybranowski S, Wilke C, van Rooijen N, Teitz-Tennenbaum S, Osterholzer JJ, et al. Resident alveolar macrophages suppress, whereas recruited monocytes promote, allergic lung inflammation in murine models of asthma. J Immunol 2014, 193(8): 4245-4253.

34. Han W, Tanjore H, Liu Y, Hunt RP, Gutor SS, Serezani APM, et al. Identification and Characterization of Alveolar and Recruited Lung Macrophages during Acute Lung Inflammation. J Immunol 2023, 210(11): 1827-1836.

35. Li Y, Li J, Yu Q, Ji L, Peng B. METTL14 regulates microglia/macrophage polarization and NLRP3 inflammasome activation after ischemic stroke by the KAT3B-STING axis. Neurobiol Dis 2023, 185: 106253.

36. Zheng Y, Li Y, Ran X, Wang D, Zheng X, Zhang M, et al. Mettl14 mediates the inflammatory response of macrophages in atherosclerosis through the NF-kappaB/IL-6 signaling pathway. Cell Mol Life Sci 2022, 79(6): 311.

37. Dang W, Tao Y, Xu X, Zhao H, Zou L, Li Y. The role of lung macrophages in acute respiratory distress syndrome. Inflamm Res 2022, 71(12): 1417-1432.

1. Meyer NJ, Gattinoni L, Calfee CS. Acute respiratory distress syndrome. Lancet 2021, 398(10300): 622-637.

2. Gorman EA, O'Kane CM, McAuley DF. Acute respiratory distress syndrome in adults: diagnosis, outcomes, long-term sequelae, and management. Lancet 2022, 400(10358): 1157-1170.

3. Bos LDJ, Ware LB. Acute respiratory distress syndrome: causes, pathophysiology, and phenotypes. Lancet 2022, 400(10358): 1145-1156.

4. Gilroy DW, De Maeyer RPH, Tepper M, O'Brien A, Uddin M, Chen J, et al. Treating exuberant, non-resolving inflammation in the lung; Implications for acute respiratory distress syndrome and COVID-19. Pharmacol Ther 2021, 221: 107745.

5. Laffey JG, Matthay MA. Fifty Years of Research in ARDS. Cell-based Therapy for Acute Respiratory Distress Syndrome. Biology and Potential Therapeutic Value. Am J Respir Crit Care Med 2017, 196(3): 266-273.

6. Xian H, Liu Y, Rundberg Nilsson A, Gatchalian R, Crother TR, Tourtellotte WG, et al. Metformin inhibition of mitochondrial ATP and DNA synthesis abrogates NLRP3 inflammasome activation and pulmonary inflammation. Immunity 2021, 54(7): 1463-1477 e1411.

7. Paik S, Kim JK, Silwal P, Sasakawa C, Jo EK. An update on the regulatory mechanisms of NLRP3 inflammasome activation. Cell Mol Immunol 2021, 18(5): 1141-1160.

8. Kelley N, Jeltema D, Duan Y, He Y. The NLRP3 Inflammasome: An Overview of Mechanisms of Activation and Regulation. Int J Mol Sci 2019, 20(13).

9. Swanson KV, Deng M, Ting JP. The NLRP3 inflammasome: molecular activation and regulation to therapeutics. Nat Rev Immunol 2019, 19(8): 477-489.

10. Bauernfeind FG, Horvath G, Stutz A, Alnemri ES, MacDonald K, Speert D, et al. Cutting edge: NF-kappaB activating pattern recognition and cytokine receptors license NLRP3 inflammasome activation by regulating NLRP3 expression. J Immunol 2009, 183(2): 787-791.

11. Cornut M, Bourdonnay E, Henry T. Transcriptional Regulation of Inflammasomes. Int J Mol Sci 2020, 21(21).

12. Baker PJ, De Nardo D, Moghaddas F, Tran LS, Bachem A, Nguyen T, et al. Posttranslational Modification as a Critical Determinant of Cytoplasmic Innate Immune Recognition. Physiol Rev 2017, 97(3): 1165-1209.

13. Moretti J, Blander JM. Increasing complexity of NLRP3 inflammasome regulation. J Leukoc Biol 2021, 109(3): 561-571.

14. Liu Y, Yang D, Liu T, Chen J, Yu J, Yi P. N6-methyladenosine-mediated gene regulation and therapeutic implications. Trends Mol Med 2023, 29(6): 454-467.

15. Zaccara S, Ries RJ, Jaffrey SR. Reading, writing and erasing mRNA methylation. Nat Rev Mol Cell Biol 2019, 20(10): 608-624.

16. Zhao Y, Shi Y, Shen H, Xie W. m(6)A-binding proteins: the emerging crucial performers in epigenetics. J Hematol Oncol 2020, 13(1): 35.

17. Xu H, Lin C, Yang J, Chen X, Chen Y, Chen J, et al. The Role of N(6)-Methyladenosine in Inflammatory Diseases. Oxid Med Cell Longev 2022, 2022: 9744771.

18. Peng Z, Gong Y, Wang X, He W, Wu L, Zhang L, et al. METTL3-m(6)A-Rubicon axis inhibits autophagy in nonalcoholic fatty liver disease. Mol Ther 2022, 30(2): 932-946.

19. Zhang J, Song B, Zeng Y, Xu C, Gao L, Guo Y, et al. m6A modification in inflammatory bowel disease provides new insights into clinical applications. Biomed Pharmacother 2023, 159: 114298.

20. Winkler R, Gillis E, Lasman L, Safra M, Geula S, Soyris C, et al. m(6)A modification controls the innate immune response to infection by targeting type I interferons. Nat Immunol 2019, 20(2): 173-182.

21. Zhang H, Liu J, Zhou Y, Qu M, Wang Y, Guo K, et al. Neutrophil extracellular traps mediate m(6)A modification and regulates sepsis-associated acute lung injury by activating ferroptosis in alveolar epithelial cells. Int J Biol Sci 2022, 18(8): 3337-3357.

22. Jiao Y, Zhang T, Zhang C, Ji H, Tong X, Xia R, et al. Exosomal miR-30d-5p of neutrophils induces M1 macrophage polarization and primes macrophage pyroptosis in sepsis-related acute lung injury. Crit Care 2021, 25(1): 356.

23. Huang X, Zhang H, Guo X, Zhu Z, Cai H, Kong X. Insulin-like growth factor 2 mRNA-binding protein 1 (IGF2BP1) in cancer. J Hematol Oncol 2018, 11(1): 88.

24. Huang H, Weng H, Chen J. m(6)A Modification in Coding and Non-coding RNAs: Roles and Therapeutic Implications in Cancer. Cancer Cell 2020, 37(3): 270-288.

26. Kumari R, Ranjan P, Suleiman ZG, Goswami SK, Li J, Prasad R, et al. mRNA modifications in cardiovascular biology and disease: with a focus on m6A modification. Cardiovasc Res 2022, 118(7): 1680-1692.

27. Luo J, Xu T, Sun K. N6-Methyladenosine RNA Modification in Inflammation: Roles, Mechanisms, and Applications. Front Cell Dev Biol 2021, 9: 670711.

28. Weng H, Huang H, Wu H, Qin X, Zhao BS, Dong L, et al. METTL14 Inhibits Hematopoietic Stem/Progenitor Differentiation and Promotes Leukemogenesis via mRNA m(6)A Modification. Cell Stem Cell 2018, 22(2): 191-205 e199.

29. Zhang H, Wu D, Wang Y, Guo K, Spencer CB, Ortoga L, et al. METTL3-mediated N6-methyladenosine exacerbates ferroptosis via m6A-IGF2BP2-dependent mitochondrial metabolic reprogramming in sepsis-induced acute lung injury. Clin Transl Med 2023, 13(9): e1389.

30. Qu M, Chen Z, Qiu Z, Nan K, Wang Y, Shi Y, et al. Neutrophil extracellular traps-triggered impaired autophagic flux via METTL3 underlies sepsis-associated acute lung injury. Cell Death Discov 2022, 8(1): 375.

31. Chen Y, Wu Y, Zhu L, Chen C, Xu S, Tang D, et al. METTL3-Mediated N6-Methyladenosine Modification of Trim59 mRNA Protects Against Sepsis-Induced Acute Respiratory Distress Syndrome. Front Immunol 2022, 13: 897487.

32. Chen X, Tang J, Shuai W, Meng J, Feng J, Han Z. Macrophage polarization and its role in the pathogenesis of acute lung injury/acute respiratory distress syndrome. Inflamm Res 2020, 69(9): 883-895.

34. Han W, Tanjore H, Liu Y, Hunt RP, Gutor SS, Serezani APM, et al. Identification and Characterization of Alveolar and Recruited Lung Macrophages during Acute Lung Inflammation. J Immunol 2023, 210(11): 1827-1836.

35. Li Y, Li J, Yu Q, Ji L, Peng B. METTL14 regulates microglia/macrophage polarization and NLRP3 inflammasome activation after ischemic stroke by the KAT3B-STING axis. Neurobiol Dis 2023, 185: 106253.

36. Zheng Y, Li Y, Ran X, Wang D, Zheng X, Zhang M, et al. Mettl14 mediates the inflammatory response of macrophages in atherosclerosis through the NF-kappaB/IL-6 signaling pathway. Cell Mol Life Sci 2022, 79(6): 311.

37. Dang W, Tao Y, Xu X, Zhao H, Zou L, Li Y. The role of lung macrophages in acute respiratory distress syndrome. Inflamm Res 2022, 71(12): 1417-1432.

38. Wang X, Ding Y, Li R, Zhang R, Ge X, Gao R, et al. N(6)-methyladenosine of Spi2a attenuates inflammation and sepsis-associated myocardial dysfunction in mice. Nat Commun 2023, 14(1): 1185.

1. Meyer NJ, Gattinoni L, Calfee CS. Acute respiratory distress syndrome. Lancet 2021, 398(10300): 622-637.

2. Gorman EA, O'Kane CM, McAuley DF. Acute respiratory distress syndrome in adults: diagnosis, outcomes, long-term sequelae, and management. Lancet 2022, 400(10358): 1157-1170.

3. Bos LDJ, Ware LB. Acute respiratory distress syndrome: causes, pathophysiology, and phenotypes. Lancet 2022, 400(10358): 1145-1156.

4. Gilroy DW, De Maeyer RPH, Tepper M, O'Brien A, Uddin M, Chen J, et al. Treating exuberant, non-resolving inflammation in the lung; Implications for acute respiratory distress syndrome and COVID-19. Pharmacol Ther 2021, 221: 107745.

5. Laffey JG, Matthay MA. Fifty Years of Research in ARDS. Cell-based Therapy for Acute Respiratory Distress Syndrome. Biology and Potential Therapeutic Value. Am J Respir Crit Care Med 2017, 196(3): 266-273.

6. Xian H, Liu Y, Rundberg Nilsson A, Gatchalian R, Crother TR, Tourtellotte WG, et al. Metformin inhibition of mitochondrial ATP and DNA synthesis abrogates NLRP3 inflammasome activation and pulmonary inflammation. Immunity 2021, 54(7): 1463-1477 e1411.

7. Paik S, Kim JK, Silwal P, Sasakawa C, Jo EK. An update on the regulatory mechanisms of NLRP3 inflammasome activation. Cell Mol Immunol 2021, 18(5): 1141-1160.

8. Kelley N, Jeltema D, Duan Y, He Y. The NLRP3 Inflammasome: An Overview of Mechanisms of Activation and Regulation. Int J Mol Sci 2019, 20(13).

9. Swanson KV, Deng M, Ting JP. The NLRP3 inflammasome: molecular activation and regulation to therapeutics. Nat Rev Immunol 2019, 19(8): 477-489.

10. Bauernfeind FG, Horvath G, Stutz A, Alnemri ES, MacDonald K, Speert D, et al. Cutting edge: NF-kappaB activating pattern recognition and cytokine receptors license NLRP3 inflammasome activation by regulating NLRP3 expression. J Immunol 2009, 183(2): 787-791.

11. Cornut M, Bourdonnay E, Henry T. Transcriptional Regulation of Inflammasomes. Int J Mol Sci 2020, 21(21).

12. Baker PJ, De Nardo D, Moghaddas F, Tran LS, Bachem A, Nguyen T, et al. Posttranslational Modification as a Critical Determinant of Cytoplasmic Innate Immune Recognition. Physiol Rev 2017, 97(3): 1165-1209.

13. Moretti J, Blander JM. Increasing complexity of NLRP3 inflammasome regulation. J Leukoc Biol 2021, 109(3): 561-571.

14. Liu Y, Yang D, Liu T, Chen J, Yu J, Yi P. N6-methyladenosine-mediated gene regulation and therapeutic implications. Trends Mol Med 2023, 29(6): 454-467.

15. Zaccara S, Ries RJ, Jaffrey SR. Reading, writing and erasing mRNA methylation. Nat Rev Mol Cell Biol 2019, 20(10): 608-624.

16. Zhao Y, Shi Y, Shen H, Xie W. m(6)A-binding proteins: the emerging crucial performers in epigenetics. J Hematol Oncol 2020, 13(1): 35.

17. Xu H, Lin C, Yang J, Chen X, Chen Y, Chen J, et al. The Role of N(6)-Methyladenosine in Inflammatory Diseases. Oxid Med Cell Longev 2022, 2022: 9744771.

18. Peng Z, Gong Y, Wang X, He W, Wu L, Zhang L, et al. METTL3-m(6)A-Rubicon axis inhibits autophagy in nonalcoholic fatty liver disease. Mol Ther 2022, 30(2): 932-946.

19. Zhang J, Song B, Zeng Y, Xu C, Gao L, Guo Y, et al. m6A modification in inflammatory bowel disease provides new insights into clinical applications. Biomed Pharmacother 2023, 159: 114298.

20. Winkler R, Gillis E, Lasman L, Safra M, Geula S, Soyris C, et al. m(6)A modification controls the innate immune response to infection by targeting type I interferons. Nat Immunol 2019, 20(2): 173-182.

21. Zhang H, Liu J, Zhou Y, Qu M, Wang Y, Guo K, et al. Neutrophil extracellular traps mediate m(6)A modification and regulates sepsis-associated acute lung injury by activating ferroptosis in alveolar epithelial cells. Int J Biol Sci 2022, 18(8): 3337-3357.

22. Jiao Y, Zhang T, Zhang C, Ji H, Tong X, Xia R, et al. Exosomal miR-30d-5p of neutrophils induces M1 macrophage polarization and primes macrophage pyroptosis in sepsis-related acute lung injury. Crit Care 2021, 25(1): 356.

23. Huang X, Zhang H, Guo X, Zhu Z, Cai H, Kong X. Insulin-like growth factor 2 mRNA-binding protein 1 (IGF2BP1) in cancer. J Hematol Oncol 2018, 11(1): 88.

24. Huang H, Weng H, Chen J. m(6)A Modification in Coding and Non-coding RNAs: Roles and Therapeutic Implications in Cancer. Cancer Cell 2020, 37(3): 270-288.

25. Zhu X, Tang H, Yang M, Yin K. N6-methyladenosine in macrophage function: a novel target for metabolic diseases. Trends Endocrinol Metab 2023, 34(2): 66-84.

26. Kumari R, Ranjan P, Suleiman ZG, Goswami SK, Li J, Prasad R, et al. mRNA modifications in cardiovascular biology and disease: with a focus on m6A modification. Cardiovasc Res 2022, 118(7): 1680-1692.

27. Luo J, Xu T, Sun K. N6-Methyladenosine RNA Modification in Inflammation: Roles, Mechanisms, and Applications. Front Cell Dev Biol 2021, 9: 670711.

28. Weng H, Huang H, Wu H, Qin X, Zhao BS, Dong L, et al. METTL14 Inhibits Hematopoietic Stem/Progenitor Differentiation and Promotes Leukemogenesis via mRNA m(6)A Modification. Cell Stem Cell 2018, 22(2): 191-205 e199.

29. Zhang H, Wu D, Wang Y, Guo K, Spencer CB, Ortoga L, et al. METTL3-mediated N6-methyladenosine exacerbates ferroptosis via m6A-IGF2BP2-dependent mitochondrial metabolic reprogramming in sepsis-induced acute lung injury. Clin Transl Med 2023, 13(9): e1389.

30. Qu M, Chen Z, Qiu Z, Nan K, Wang Y, Shi Y, et al. Neutrophil extracellular traps-triggered impaired autophagic flux via METTL3 underlies sepsis-associated acute lung injury. Cell Death Discov 2022, 8(1): 375.

31. Chen Y, Wu Y, Zhu L, Chen C, Xu S, Tang D, et al. METTL3-Mediated N6-Methyladenosine Modification of Trim59 mRNA Protects Against Sepsis-Induced Acute Respiratory Distress Syndrome. Front Immunol 2022, 13: 897487.

32. Chen X, Tang J, Shuai W, Meng J, Feng J, Han Z. Macrophage polarization and its role in the pathogenesis of acute lung injury/acute respiratory distress syndrome. Inflamm Res 2020, 69(9): 883-895.

33. Zaslona Z, Przybranowski S, Wilke C, van Rooijen N, Teitz-Tennenbaum S, Osterholzer JJ, et al. Resident alveolar macrophages suppress, whereas recruited monocytes promote, allergic lung inflammation in murine models of asthma. J Immunol 2014, 193(8): 4245-4253.

34. Han W, Tanjore H, Liu Y, Hunt RP, Gutor SS, Serezani APM, et al. Identification and Characterization of Alveolar and Recruited Lung Macrophages during Acute Lung Inflammation. J Immunol 2023, 210(11): 1827-1836.

35. Li Y, Li J, Yu Q, Ji L, Peng B. METTL14 regulates microglia/macrophage polarization and NLRP3 inflammasome activation after ischemic stroke by the KAT3B-STING axis. Neurobiol Dis 2023, 185: 106253.

36. Zheng Y, Li Y, Ran X, Wang D, Zheng X, Zhang M, et al. Mettl14 mediates the inflammatory response of macrophages in atherosclerosis through the NF-kappaB/IL-6 signaling pathway. Cell Mol Life Sci 2022, 79(6): 311.

37. Dang W, Tao Y, Xu X, Zhao H, Zou L, Li Y. The role of lung macrophages in acute respiratory distress syndrome. Inflamm Res 2022, 71(12): 1417-1432.

39. Mangan MSJ, Olhava EJ, Roush WR, Seidel HM, Glick GD, Latz E. Targeting the NLRP3 inflammasome in inflammatory diseases. Nat Rev Drug Discov 2018, 17(8): 588-606.

1. Meyer NJ, Gattinoni L, Calfee CS. Acute respiratory distress syndrome. Lancet 2021, 398(10300): 622-637.

2. Gorman EA, O'Kane CM, McAuley DF. Acute respiratory distress syndrome in adults: diagnosis, outcomes, long-term sequelae, and management. Lancet 2022, 400(10358): 1157-1170.

3. Bos LDJ, Ware LB. Acute respiratory distress syndrome: causes, pathophysiology, and phenotypes. Lancet 2022, 400(10358): 1145-1156.

4. Gilroy DW, De Maeyer RPH, Tepper M, O'Brien A, Uddin M, Chen J, et al. Treating exuberant, non-resolving inflammation in the lung; Implications for acute respiratory distress syndrome and COVID-19. Pharmacol Ther 2021, 221: 107745.

5. Laffey JG, Matthay MA. Fifty Years of Research in ARDS. Cell-based Therapy for Acute Respiratory Distress Syndrome. Biology and Potential Therapeutic Value. Am J Respir Crit Care Med 2017, 196(3): 266-273.

6. Xian H, Liu Y, Rundberg Nilsson A, Gatchalian R, Crother TR, Tourtellotte WG, et al. Metformin inhibition of mitochondrial ATP and DNA synthesis abrogates NLRP3 inflammasome activation and pulmonary inflammation. Immunity 2021, 54(7): 1463-1477 e1411.

7. Paik S, Kim JK, Silwal P, Sasakawa C, Jo EK. An update on the regulatory mechanisms of NLRP3 inflammasome activation. Cell Mol Immunol 2021, 18(5): 1141-1160.

8. Kelley N, Jeltema D, Duan Y, He Y. The NLRP3 Inflammasome: An Overview of Mechanisms of Activation and Regulation. Int J Mol Sci 2019, 20(13).

9. Swanson KV, Deng M, Ting JP. The NLRP3 inflammasome: molecular activation and regulation to therapeutics. Nat Rev Immunol 2019, 19(8): 477-489.

10. Bauernfeind FG, Horvath G, Stutz A, Alnemri ES, MacDonald K, Speert D, et al. Cutting edge: NF-kappaB activating pattern recognition and cytokine receptors license NLRP3 inflammasome activation by regulating NLRP3 expression. J Immunol 2009, 183(2): 787-791.

11. Cornut M, Bourdonnay E, Henry T. Transcriptional Regulation of Inflammasomes. Int J Mol Sci 2020, 21(21).

12. Baker PJ, De Nardo D, Moghaddas F, Tran LS, Bachem A, Nguyen T, et al. Posttranslational Modification as a Critical Determinant of Cytoplasmic Innate Immune Recognition. Physiol Rev 2017, 97(3): 1165-1209.

13. Moretti J, Blander JM. Increasing complexity of NLRP3 inflammasome regulation. J Leukoc Biol 2021, 109(3): 561-571.

14. Liu Y, Yang D, Liu T, Chen J, Yu J, Yi P. N6-methyladenosine-mediated gene regulation and therapeutic implications. Trends Mol Med 2023, 29(6): 454-467.

15. Zaccara S, Ries RJ, Jaffrey SR. Reading, writing and erasing mRNA methylation. Nat Rev Mol Cell Biol 2019, 20(10): 608-624.

16. Zhao Y, Shi Y, Shen H, Xie W. m(6)A-binding proteins: the emerging crucial performers in epigenetics. J Hematol Oncol 2020, 13(1): 35.

17. Xu H, Lin C, Yang J, Chen X, Chen Y, Chen J, et al. The Role of N(6)-Methyladenosine in Inflammatory Diseases. Oxid Med Cell Longev 2022, 2022: 9744771.

18. Peng Z, Gong Y, Wang X, He W, Wu L, Zhang L, et al. METTL3-m(6)A-Rubicon axis inhibits autophagy in nonalcoholic fatty liver disease. Mol Ther 2022, 30(2): 932-946.

19. Zhang J, Song B, Zeng Y, Xu C, Gao L, Guo Y, et al. m6A modification in inflammatory bowel disease provides new insights into clinical applications. Biomed Pharmacother 2023, 159: 114298.

20. Winkler R, Gillis E, Lasman L, Safra M, Geula S, Soyris C, et al. m(6)A modification controls the innate immune response to infection by targeting type I interferons. Nat Immunol 2019, 20(2): 173-182.

21. Zhang H, Liu J, Zhou Y, Qu M, Wang Y, Guo K, et al. Neutrophil extracellular traps mediate m(6)A modification and regulates sepsis-associated acute lung injury by activating ferroptosis in alveolar epithelial cells. Int J Biol Sci 2022, 18(8): 3337-3357.

22. Jiao Y, Zhang T, Zhang C, Ji H, Tong X, Xia R, et al. Exosomal miR-30d-5p of neutrophils induces M1 macrophage polarization and primes macrophage pyroptosis in sepsis-related acute lung injury. Crit Care 2021, 25(1): 356.

23. Huang X, Zhang H, Guo X, Zhu Z, Cai H, Kong X. Insulin-like growth factor 2 mRNA-binding protein 1 (IGF2BP1) in cancer. J Hematol Oncol 2018, 11(1): 88.

24. Huang H, Weng H, Chen J. m(6)A Modification in Coding and Non-coding RNAs: Roles and Therapeutic Implications in Cancer. Cancer Cell 2020, 37(3): 270-288.

25. Zhu X, Tang H, Yang M, Yin K. N6-methyladenosine in macrophage function: a novel target for metabolic diseases. Trends Endocrinol Metab 2023, 34(2): 66-84.

26. Kumari R, Ranjan P, Suleiman ZG, Goswami SK, Li J, Prasad R, et al. mRNA modifications in cardiovascular biology and disease: with a focus on m6A modification. Cardiovasc Res 2022, 118(7): 1680-1692.

27. Luo J, Xu T, Sun K. N6-Methyladenosine RNA Modification in Inflammation: Roles, Mechanisms, and Applications. Front Cell Dev Biol 2021, 9: 670711.

28. Weng H, Huang H, Wu H, Qin X, Zhao BS, Dong L, et al. METTL14 Inhibits Hematopoietic Stem/Progenitor Differentiation and Promotes Leukemogenesis via mRNA m(6)A Modification. Cell Stem Cell 2018, 22(2): 191-205 e199.

29. Zhang H, Wu D, Wang Y, Guo K, Spencer CB, Ortoga L, et al. METTL3-mediated N6-methyladenosine exacerbates ferroptosis via m6A-IGF2BP2-dependent mitochondrial metabolic reprogramming in sepsis-induced acute lung injury. Clin Transl Med 2023, 13(9): e1389.

30. Qu M, Chen Z, Qiu Z, Nan K, Wang Y, Shi Y, et al. Neutrophil extracellular traps-triggered impaired autophagic flux via METTL3 underlies sepsis-associated acute lung injury. Cell Death Discov 2022, 8(1): 375.

31. Chen Y, Wu Y, Zhu L, Chen C, Xu S, Tang D, et al. METTL3-Mediated N6-Methyladenosine Modification of Trim59 mRNA Protects Against Sepsis-Induced Acute Respiratory Distress Syndrome. Front Immunol 2022, 13: 897487.

32. Chen X, Tang J, Shuai W, Meng J, Feng J, Han Z. Macrophage polarization and its role in the pathogenesis of acute lung injury/acute respiratory distress syndrome. Inflamm Res 2020, 69(9): 883-895.

33. Zaslona Z, Przybranowski S, Wilke C, van Rooijen N, Teitz-Tennenbaum S, Osterholzer JJ, et al. Resident alveolar macrophages suppress, whereas recruited monocytes promote, allergic lung inflammation in murine models of asthma. J Immunol 2014, 193(8): 4245-4253.

34. Han W, Tanjore H, Liu Y, Hunt RP, Gutor SS, Serezani APM, et al. Identification and Characterization of Alveolar and Recruited Lung Macrophages during Acute Lung Inflammation. J Immunol 2023, 210(11): 1827-1836.

35. Li Y, Li J, Yu Q, Ji L, Peng B. METTL14 regulates microglia/macrophage polarization and NLRP3 inflammasome activation after ischemic stroke by the KAT3B-STING axis. Neurobiol Dis 2023, 185: 106253.

36. Zheng Y, Li Y, Ran X, Wang D, Zheng X, Zhang M, et al. Mettl14 mediates the inflammatory response of macrophages in atherosclerosis through the NF-kappaB/IL-6 signaling pathway. Cell Mol Life Sci 2022, 79(6): 311.

37. Dang W, Tao Y, Xu X, Zhao H, Zou L, Li Y. The role of lung macrophages in acute respiratory distress syndrome. Inflamm Res 2022, 71(12): 1417-1432.

38. Wang X, Ding Y, Li R, Zhang R, Ge X, Gao R, et al. N(6)-methyladenosine of Spi2a attenuates inflammation and sepsis-associated myocardial dysfunction in mice. Nat Commun 2023, 14(1): 1185.

39. Mangan MSJ, Olhava EJ, Roush WR, Seidel HM, Glick GD, Latz E. Targeting the NLRP3 inflammasome in inflammatory diseases. Nat Rev Drug Discov 2018, 17(8): 588-606.

40. Wann SR, Chi PL, Huang WC, Cheng CC, Chang YT. Combination therapy of iPSC-derived conditioned medium with ceftriaxone alleviates bacteria-induced lung injury by targeting the NLRP3 inflammasome. J Cell Physiol 2022, 237(2): 1299-1314.

1. Meyer NJ, Gattinoni L, Calfee CS. Acute respiratory distress syndrome. Lancet 2021, 398(10300): 622-637.

2. Gorman EA, O'Kane CM, McAuley DF. Acute respiratory distress syndrome in adults: diagnosis, outcomes, long-term sequelae, and management. Lancet 2022, 400(10358): 1157-1170.

3. Bos LDJ, Ware LB. Acute respiratory distress syndrome: causes, pathophysiology, and phenotypes. Lancet 2022, 400(10358): 1145-1156.

4. Gilroy DW, De Maeyer RPH, Tepper M, O'Brien A, Uddin M, Chen J, et al. Treating exuberant, non-resolving inflammation in the lung; Implications for acute respiratory distress syndrome and COVID-19. Pharmacol Ther 2021, 221: 107745.

5. Laffey JG, Matthay MA. Fifty Years of Research in ARDS. Cell-based Therapy for Acute Respiratory Distress Syndrome. Biology and Potential Therapeutic Value. Am J Respir Crit Care Med 2017, 196(3): 266-273.

6. Xian H, Liu Y, Rundberg Nilsson A, Gatchalian R, Crother TR, Tourtellotte WG, et al. Metformin inhibition of mitochondrial ATP and DNA synthesis abrogates NLRP3 inflammasome activation and pulmonary inflammation. Immunity 2021, 54(7): 1463-1477 e1411.

7. Paik S, Kim JK, Silwal P, Sasakawa C, Jo EK. An update on the regulatory mechanisms of NLRP3 inflammasome activation. Cell Mol Immunol 2021, 18(5): 1141-1160.

8. Kelley N, Jeltema D, Duan Y, He Y. The NLRP3 Inflammasome: An Overview of Mechanisms of Activation and Regulation. Int J Mol Sci 2019, 20(13).

9. Swanson KV, Deng M, Ting JP. The NLRP3 inflammasome: molecular activation and regulation to therapeutics. Nat Rev Immunol 2019, 19(8): 477-489.

10. Bauernfeind FG, Horvath G, Stutz A, Alnemri ES, MacDonald K, Speert D, et al. Cutting edge: NF-kappaB activating pattern recognition and cytokine receptors license NLRP3 inflammasome activation by regulating NLRP3 expression. J Immunol 2009, 183(2): 787-791.

11. Cornut M, Bourdonnay E, Henry T. Transcriptional Regulation of Inflammasomes. Int J Mol Sci 2020, 21(21).

12. Baker PJ, De Nardo D, Moghaddas F, Tran LS, Bachem A, Nguyen T, et al. Posttranslational Modification as a Critical Determinant of Cytoplasmic Innate Immune Recognition. Physiol Rev 2017, 97(3): 1165-1209.

13. Moretti J, Blander JM. Increasing complexity of NLRP3 inflammasome regulation. J Leukoc Biol 2021, 109(3): 561-571.

14. Liu Y, Yang D, Liu T, Chen J, Yu J, Yi P. N6-methyladenosine-mediated gene regulation and therapeutic implications. Trends Mol Med 2023, 29(6): 454-467.

15. Zaccara S, Ries RJ, Jaffrey SR. Reading, writing and erasing mRNA methylation. Nat Rev Mol Cell Biol 2019, 20(10): 608-624.

16. Zhao Y, Shi Y, Shen H, Xie W. m(6)A-binding proteins: the emerging crucial performers in epigenetics. J Hematol Oncol 2020, 13(1): 35.

17. Xu H, Lin C, Yang J, Chen X, Chen Y, Chen J, et al. The Role of N(6)-Methyladenosine in Inflammatory Diseases. Oxid Med Cell Longev 2022, 2022: 9744771.

18. Peng Z, Gong Y, Wang X, He W, Wu L, Zhang L, et al. METTL3-m(6)A-Rubicon axis inhibits autophagy in nonalcoholic fatty liver disease. Mol Ther 2022, 30(2): 932-946.

19. Zhang J, Song B, Zeng Y, Xu C, Gao L, Guo Y, et al. m6A modification in inflammatory bowel disease provides new insights into clinical applications. Biomed Pharmacother 2023, 159: 114298.

20. Winkler R, Gillis E, Lasman L, Safra M, Geula S, Soyris C, et al. m(6)A modification controls the innate immune response to infection by targeting type I interferons. Nat Immunol 2019, 20(2): 173-182.

21. Zhang H, Liu J, Zhou Y, Qu M, Wang Y, Guo K, et al. Neutrophil extracellular traps mediate m(6)A modification and regulates sepsis-associated acute lung injury by activating ferroptosis in alveolar epithelial cells. Int J Biol Sci 2022, 18(8): 3337-3357.

22. Jiao Y, Zhang T, Zhang C, Ji H, Tong X, Xia R, et al. Exosomal miR-30d-5p of neutrophils induces M1 macrophage polarization and primes macrophage pyroptosis in sepsis-related acute lung injury. Crit Care 2021, 25(1): 356.

23. Huang X, Zhang H, Guo X, Zhu Z, Cai H, Kong X. Insulin-like growth factor 2 mRNA-binding protein 1 (IGF2BP1) in cancer. J Hematol Oncol 2018, 11(1): 88.

24. Huang H, Weng H, Chen J. m(6)A Modification in Coding and Non-coding RNAs: Roles and Therapeutic Implications in Cancer. Cancer Cell 2020, 37(3): 270-288.

25. Zhu X, Tang H, Yang M, Yin K. N6-methyladenosine in macrophage function: a novel target for metabolic diseases. Trends Endocrinol Metab 2023, 34(2): 66-84.

26. Kumari R, Ranjan P, Suleiman ZG, Goswami SK, Li J, Prasad R, et al. mRNA modifications in cardiovascular biology and disease: with a focus on m6A modification. Cardiovasc Res 2022, 118(7): 1680-1692.

27. Luo J, Xu T, Sun K. N6-Methyladenosine RNA Modification in Inflammation: Roles, Mechanisms, and Applications. Front Cell Dev Biol 2021, 9: 670711.

28. Weng H, Huang H, Wu H, Qin X, Zhao BS, Dong L, et al. METTL14 Inhibits Hematopoietic Stem/Progenitor Differentiation and Promotes Leukemogenesis via mRNA m(6)A Modification. Cell Stem Cell 2018, 22(2): 191-205 e199.

29. Zhang H, Wu D, Wang Y, Guo K, Spencer CB, Ortoga L, et al. METTL3-mediated N6-methyladenosine exacerbates ferroptosis via m6A-IGF2BP2-dependent mitochondrial metabolic reprogramming in sepsis-induced acute lung injury. Clin Transl Med 2023, 13(9): e1389.

30. Qu M, Chen Z, Qiu Z, Nan K, Wang Y, Shi Y, et al. Neutrophil extracellular traps-triggered impaired autophagic flux via METTL3 underlies sepsis-associated acute lung injury. Cell Death Discov 2022, 8(1): 375.

31. Chen Y, Wu Y, Zhu L, Chen C, Xu S, Tang D, et al. METTL3-Mediated N6-Methyladenosine Modification of Trim59 mRNA Protects Against Sepsis-Induced Acute Respiratory Distress Syndrome. Front Immunol 2022, 13: 897487.

32. Chen X, Tang J, Shuai W, Meng J, Feng J, Han Z. Macrophage polarization and its role in the pathogenesis of acute lung injury/acute respiratory distress syndrome. Inflamm Res 2020, 69(9): 883-895.

33. Zaslona Z, Przybranowski S, Wilke C, van Rooijen N, Teitz-Tennenbaum S, Osterholzer JJ, et al. Resident alveolar macrophages suppress, whereas recruited monocytes promote, allergic lung inflammation in murine models of asthma. J Immunol 2014, 193(8): 4245-4253.

34. Han W, Tanjore H, Liu Y, Hunt RP, Gutor SS, Serezani APM, et al. Identification and Characterization of Alveolar and Recruited Lung Macrophages during Acute Lung Inflammation. J Immunol 2023, 210(11): 1827-1836.

35. Li Y, Li J, Yu Q, Ji L, Peng B. METTL14 regulates microglia/macrophage polarization and NLRP3 inflammasome activation after ischemic stroke by the KAT3B-STING axis. Neurobiol Dis 2023, 185: 106253.

36. Zheng Y, Li Y, Ran X, Wang D, Zheng X, Zhang M, et al. Mettl14 mediates the inflammatory response of macrophages in atherosclerosis through the NF-kappaB/IL-6 signaling pathway. Cell Mol Life Sci 2022, 79(6): 311.

37. Dang W, Tao Y, Xu X, Zhao H, Zou L, Li Y. The role of lung macrophages in acute respiratory distress syndrome. Inflamm Res 2022, 71(12): 1417-1432.

38. Wang X, Ding Y, Li R, Zhang R, Ge X, Gao R, et al. N(6)-methyladenosine of Spi2a attenuates inflammation and sepsis-associated myocardial dysfunction in mice. Nat Commun 2023, 14(1): 1185.

40. Wann SR, Chi PL, Huang WC, Cheng CC, Chang YT. Combination therapy of iPSC-derived conditioned medium with ceftriaxone alleviates bacteria-induced lung injury by targeting the NLRP3 inflammasome. J Cell Physiol 2022, 237(2): 1299-1314.

41. Xu Q, Ye Y, Wang Z, Zhu H, Li Y, Wang J, et al. NLRP3 Knockout Protects against Lung Injury Induced by Cerebral Ischemia-Reperfusion. Oxid Med Cell Longev 2022, 2022: 6260102.

1. Meyer NJ, Gattinoni L, Calfee CS. Acute respiratory distress syndrome. Lancet 2021, 398(10300): 622-637.

2. Gorman EA, O'Kane CM, McAuley DF. Acute respiratory distress syndrome in adults: diagnosis, outcomes, long-term sequelae, and management. Lancet 2022, 400(10358): 1157-1170.

3. Bos LDJ, Ware LB. Acute respiratory distress syndrome: causes, pathophysiology, and phenotypes. Lancet 2022, 400(10358): 1145-1156.

4. Gilroy DW, De Maeyer RPH, Tepper M, O'Brien A, Uddin M, Chen J, et al. Treating exuberant, non-resolving inflammation in the lung; Implications for acute respiratory distress syndrome and COVID-19. Pharmacol Ther 2021, 221: 107745.

5. Laffey JG, Matthay MA. Fifty Years of Research in ARDS. Cell-based Therapy for Acute Respiratory Distress Syndrome. Biology and Potential Therapeutic Value. Am J Respir Crit Care Med 2017, 196(3): 266-273.

6. Xian H, Liu Y, Rundberg Nilsson A, Gatchalian R, Crother TR, Tourtellotte WG, et al. Metformin inhibition of mitochondrial ATP and DNA synthesis abrogates NLRP3 inflammasome activation and pulmonary inflammation. Immunity 2021, 54(7): 1463-1477 e1411.

7. Paik S, Kim JK, Silwal P, Sasakawa C, Jo EK. An update on the regulatory mechanisms of NLRP3 inflammasome activation. Cell Mol Immunol 2021, 18(5): 1141-1160.

8. Kelley N, Jeltema D, Duan Y, He Y. The NLRP3 Inflammasome: An Overview of Mechanisms of Activation and Regulation. Int J Mol Sci 2019, 20(13).

9. Swanson KV, Deng M, Ting JP. The NLRP3 inflammasome: molecular activation and regulation to therapeutics. Nat Rev Immunol 2019, 19(8): 477-489.

10. Bauernfeind FG, Horvath G, Stutz A, Alnemri ES, MacDonald K, Speert D, et al. Cutting edge: NF-kappaB activating pattern recognition and cytokine receptors license NLRP3 inflammasome activation by regulating NLRP3 expression. J Immunol 2009, 183(2): 787-791.

11. Cornut M, Bourdonnay E, Henry T. Transcriptional Regulation of Inflammasomes. Int J Mol Sci 2020, 21(21).

12. Baker PJ, De Nardo D, Moghaddas F, Tran LS, Bachem A, Nguyen T, et al. Posttranslational Modification as a Critical Determinant of Cytoplasmic Innate Immune Recognition. Physiol Rev 2017, 97(3): 1165-1209.

13. Moretti J, Blander JM. Increasing complexity of NLRP3 inflammasome regulation. J Leukoc Biol 2021, 109(3): 561-571.

14. Liu Y, Yang D, Liu T, Chen J, Yu J, Yi P. N6-methyladenosine-mediated gene regulation and therapeutic implications. Trends Mol Med 2023, 29(6): 454-467.

15. Zaccara S, Ries RJ, Jaffrey SR. Reading, writing and erasing mRNA methylation. Nat Rev Mol Cell Biol 2019, 20(10): 608-624.

16. Zhao Y, Shi Y, Shen H, Xie W. m(6)A-binding proteins: the emerging crucial performers in epigenetics. J Hematol Oncol 2020, 13(1): 35.

17. Xu H, Lin C, Yang J, Chen X, Chen Y, Chen J, et al. The Role of N(6)-Methyladenosine in Inflammatory Diseases. Oxid Med Cell Longev 2022, 2022: 9744771.

18. Peng Z, Gong Y, Wang X, He W, Wu L, Zhang L, et al. METTL3-m(6)A-Rubicon axis inhibits autophagy in nonalcoholic fatty liver disease. Mol Ther 2022, 30(2): 932-946.

19. Zhang J, Song B, Zeng Y, Xu C, Gao L, Guo Y, et al. m6A modification in inflammatory bowel disease provides new insights into clinical applications. Biomed Pharmacother 2023, 159: 114298.

20. Winkler R, Gillis E, Lasman L, Safra M, Geula S, Soyris C, et al. m(6)A modification controls the innate immune response to infection by targeting type I interferons. Nat Immunol 2019, 20(2): 173-182.

21. Zhang H, Liu J, Zhou Y, Qu M, Wang Y, Guo K, et al. Neutrophil extracellular traps mediate m(6)A modification and regulates sepsis-associated acute lung injury by activating ferroptosis in alveolar epithelial cells. Int J Biol Sci 2022, 18(8): 3337-3357.

22. Jiao Y, Zhang T, Zhang C, Ji H, Tong X, Xia R, et al. Exosomal miR-30d-5p of neutrophils induces M1 macrophage polarization and primes macrophage pyroptosis in sepsis-related acute lung injury. Crit Care 2021, 25(1): 356.

23. Huang X, Zhang H, Guo X, Zhu Z, Cai H, Kong X. Insulin-like growth factor 2 mRNA-binding protein 1 (IGF2BP1) in cancer. J Hematol Oncol 2018, 11(1): 88.

24. Huang H, Weng H, Chen J. m(6)A Modification in Coding and Non-coding RNAs: Roles and Therapeutic Implications in Cancer. Cancer Cell 2020, 37(3): 270-288.

25. Zhu X, Tang H, Yang M, Yin K. N6-methyladenosine in macrophage function: a novel target for metabolic diseases. Trends Endocrinol Metab 2023, 34(2): 66-84.

26. Kumari R, Ranjan P, Suleiman ZG, Goswami SK, Li J, Prasad R, et al. mRNA modifications in cardiovascular biology and disease: with a focus on m6A modification. Cardiovasc Res 2022, 118(7): 1680-1692.

27. Luo J, Xu T, Sun K. N6-Methyladenosine RNA Modification in Inflammation: Roles, Mechanisms, and Applications. Front Cell Dev Biol 2021, 9: 670711.

28. Weng H, Huang H, Wu H, Qin X, Zhao BS, Dong L, et al. METTL14 Inhibits Hematopoietic Stem/Progenitor Differentiation and Promotes Leukemogenesis via mRNA m(6)A Modification. Cell Stem Cell 2018, 22(2): 191-205 e199.

29. Zhang H, Wu D, Wang Y, Guo K, Spencer CB, Ortoga L, et al. METTL3-mediated N6-methyladenosine exacerbates ferroptosis via m6A-IGF2BP2-dependent mitochondrial metabolic reprogramming in sepsis-induced acute lung injury. Clin Transl Med 2023, 13(9): e1389.

30. Qu M, Chen Z, Qiu Z, Nan K, Wang Y, Shi Y, et al. Neutrophil extracellular traps-triggered impaired autophagic flux via METTL3 underlies sepsis-associated acute lung injury. Cell Death Discov 2022, 8(1): 375.

31. Chen Y, Wu Y, Zhu L, Chen C, Xu S, Tang D, et al. METTL3-Mediated N6-Methyladenosine Modification of Trim59 mRNA Protects Against Sepsis-Induced Acute Respiratory Distress Syndrome. Front Immunol 2022, 13: 897487.

32. Chen X, Tang J, Shuai W, Meng J, Feng J, Han Z. Macrophage polarization and its role in the pathogenesis of acute lung injury/acute respiratory distress syndrome. Inflamm Res 2020, 69(9): 883-895.

33. Zaslona Z, Przybranowski S, Wilke C, van Rooijen N, Teitz-Tennenbaum S, Osterholzer JJ, et al. Resident alveolar macrophages suppress, whereas recruited monocytes promote, allergic lung inflammation in murine models of asthma. J Immunol 2014, 193(8): 4245-4253.

34. Han W, Tanjore H, Liu Y, Hunt RP, Gutor SS, Serezani APM, et al. Identification and Characterization of Alveolar and Recruited Lung Macrophages during Acute Lung Inflammation. J Immunol 2023, 210(11): 1827-1836.

35. Li Y, Li J, Yu Q, Ji L, Peng B. METTL14 regulates microglia/macrophage polarization and NLRP3 inflammasome activation after ischemic stroke by the KAT3B-STING axis. Neurobiol Dis 2023, 185: 106253.

36. Zheng Y, Li Y, Ran X, Wang D, Zheng X, Zhang M, et al. Mettl14 mediates the inflammatory response of macrophages in atherosclerosis through the NF-kappaB/IL-6 signaling pathway. Cell Mol Life Sci 2022, 79(6): 311.

37. Dang W, Tao Y, Xu X, Zhao H, Zou L, Li Y. The role of lung macrophages in acute respiratory distress syndrome. Inflamm Res 2022, 71(12): 1417-1432.

38. Wang X, Ding Y, Li R, Zhang R, Ge X, Gao R, et al. N(6)-methyladenosine of Spi2a attenuates inflammation and sepsis-associated myocardial dysfunction in mice. Nat Commun 2023, 14(1): 1185.

39. Mangan MSJ, Olhava EJ, Roush WR, Seidel HM, Glick GD, Latz E. Targeting the NLRP3 inflammasome in inflammatory diseases. Nat Rev Drug Discov 2018, 17(8): 588-606.

40. Wann SR, Chi PL, Huang WC, Cheng CC, Chang YT. Combination therapy of iPSC-derived conditioned medium with ceftriaxone alleviates bacteria-induced lung injury by targeting the NLRP3 inflammasome. J Cell Physiol 2022, 237(2): 1299-1314.

41. Xu Q, Ye Y, Wang Z, Zhu H, Li Y, Wang J, et al. NLRP3 Knockout Protects against Lung Injury Induced by Cerebral Ischemia-Reperfusion. Oxid Med Cell Longev 2022, 2022: 6260102.

42. Li H, Guan Y, Liang B, Ding P, Hou X, Wei W, et al. Therapeutic potential of MCC950, a specific inhibitor of NLRP3 inflammasome. Eur J Pharmacol 2022, 928: 175091.

1. Meyer NJ, Gattinoni L, Calfee CS. Acute respiratory distress syndrome. Lancet 2021, 398(10300): 622-637.

2. Gorman EA, O'Kane CM, McAuley DF. Acute respiratory distress syndrome in adults: diagnosis, outcomes, long-term sequelae, and management. Lancet 2022, 400(10358): 1157-1170.

3. Bos LDJ, Ware LB. Acute respiratory distress syndrome: causes, pathophysiology, and phenotypes. Lancet 2022, 400(10358): 1145-1156.

4. Gilroy DW, De Maeyer RPH, Tepper M, O'Brien A, Uddin M, Chen J, et al. Treating exuberant, non-resolving inflammation in the lung; Implications for acute respiratory distress syndrome and COVID-19. Pharmacol Ther 2021, 221: 107745.

5. Laffey JG, Matthay MA. Fifty Years of Research in ARDS. Cell-based Therapy for Acute Respiratory Distress Syndrome. Biology and Potential Therapeutic Value. Am J Respir Crit Care Med 2017, 196(3): 266-273.

6. Xian H, Liu Y, Rundberg Nilsson A, Gatchalian R, Crother TR, Tourtellotte WG, et al. Metformin inhibition of mitochondrial ATP and DNA synthesis abrogates NLRP3 inflammasome activation and pulmonary inflammation. Immunity 2021, 54(7): 1463-1477 e1411.

7. Paik S, Kim JK, Silwal P, Sasakawa C, Jo EK. An update on the regulatory mechanisms of NLRP3 inflammasome activation. Cell Mol Immunol 2021, 18(5): 1141-1160.

8. Kelley N, Jeltema D, Duan Y, He Y. The NLRP3 Inflammasome: An Overview of Mechanisms of Activation and Regulation. Int J Mol Sci 2019, 20(13).

9. Swanson KV, Deng M, Ting JP. The NLRP3 inflammasome: molecular activation and regulation to therapeutics. Nat Rev Immunol 2019, 19(8): 477-489.

10. Bauernfeind FG, Horvath G, Stutz A, Alnemri ES, MacDonald K, Speert D, et al. Cutting edge: NF-kappaB activating pattern recognition and cytokine receptors license NLRP3 inflammasome activation by regulating NLRP3 expression. J Immunol 2009, 183(2): 787-791.

11. Cornut M, Bourdonnay E, Henry T. Transcriptional Regulation of Inflammasomes. Int J Mol Sci 2020, 21(21).

12. Baker PJ, De Nardo D, Moghaddas F, Tran LS, Bachem A, Nguyen T, et al. Posttranslational Modification as a Critical Determinant of Cytoplasmic Innate Immune Recognition. Physiol Rev 2017, 97(3): 1165-1209.

13. Moretti J, Blander JM. Increasing complexity of NLRP3 inflammasome regulation. J Leukoc Biol 2021, 109(3): 561-571.

14. Liu Y, Yang D, Liu T, Chen J, Yu J, Yi P. N6-methyladenosine-mediated gene regulation and therapeutic implications. Trends Mol Med 2023, 29(6): 454-467.

15. Zaccara S, Ries RJ, Jaffrey SR. Reading, writing and erasing mRNA methylation. Nat Rev Mol Cell Biol 2019, 20(10): 608-624.

16. Zhao Y, Shi Y, Shen H, Xie W. m(6)A-binding proteins: the emerging crucial performers in epigenetics. J Hematol Oncol 2020, 13(1): 35.

17. Xu H, Lin C, Yang J, Chen X, Chen Y, Chen J, et al. The Role of N(6)-Methyladenosine in Inflammatory Diseases. Oxid Med Cell Longev 2022, 2022: 9744771.

18. Peng Z, Gong Y, Wang X, He W, Wu L, Zhang L, et al. METTL3-m(6)A-Rubicon axis inhibits autophagy in nonalcoholic fatty liver disease. Mol Ther 2022, 30(2): 932-946.

19. Zhang J, Song B, Zeng Y, Xu C, Gao L, Guo Y, et al. m6A modification in inflammatory bowel disease provides new insights into clinical applications. Biomed Pharmacother 2023, 159: 114298.

20. Winkler R, Gillis E, Lasman L, Safra M, Geula S, Soyris C, et al. m(6)A modification controls the innate immune response to infection by targeting type I interferons. Nat Immunol 2019, 20(2): 173-182.

21. Zhang H, Liu J, Zhou Y, Qu M, Wang Y, Guo K, et al. Neutrophil extracellular traps mediate m(6)A modification and regulates sepsis-associated acute lung injury by activating ferroptosis in alveolar epithelial cells. Int J Biol Sci 2022, 18(8): 3337-3357.

22. Jiao Y, Zhang T, Zhang C, Ji H, Tong X, Xia R, et al. Exosomal miR-30d-5p of neutrophils induces M1 macrophage polarization and primes macrophage pyroptosis in sepsis-related acute lung injury. Crit Care 2021, 25(1): 356.

23. Huang X, Zhang H, Guo X, Zhu Z, Cai H, Kong X. Insulin-like growth factor 2 mRNA-binding protein 1 (IGF2BP1) in cancer. J Hematol Oncol 2018, 11(1): 88.

24. Huang H, Weng H, Chen J. m(6)A Modification in Coding and Non-coding RNAs: Roles and Therapeutic Implications in Cancer. Cancer Cell 2020, 37(3): 270-288.

25. Zhu X, Tang H, Yang M, Yin K. N6-methyladenosine in macrophage function: a novel target for metabolic diseases. Trends Endocrinol Metab 2023, 34(2): 66-84.

26. Kumari R, Ranjan P, Suleiman ZG, Goswami SK, Li J, Prasad R, et al. mRNA modifications in cardiovascular biology and disease: with a focus on m6A modification. Cardiovasc Res 2022, 118(7): 1680-1692.

27. Luo J, Xu T, Sun K. N6-Methyladenosine RNA Modification in Inflammation: Roles, Mechanisms, and Applications. Front Cell Dev Biol 2021, 9: 670711.

28. Weng H, Huang H, Wu H, Qin X, Zhao BS, Dong L, et al. METTL14 Inhibits Hematopoietic Stem/Progenitor Differentiation and Promotes Leukemogenesis via mRNA m(6)A Modification. Cell Stem Cell 2018, 22(2): 191-205 e199.

29. Zhang H, Wu D, Wang Y, Guo K, Spencer CB, Ortoga L, et al. METTL3-mediated N6-methyladenosine exacerbates ferroptosis via m6A-IGF2BP2-dependent mitochondrial metabolic reprogramming in sepsis-induced acute lung injury. Clin Transl Med 2023, 13(9): e1389.

30. Qu M, Chen Z, Qiu Z, Nan K, Wang Y, Shi Y, et al. Neutrophil extracellular traps-triggered impaired autophagic flux via METTL3 underlies sepsis-associated acute lung injury. Cell Death Discov 2022, 8(1): 375.

31. Chen Y, Wu Y, Zhu L, Chen C, Xu S, Tang D, et al. METTL3-Mediated N6-Methyladenosine Modification of Trim59 mRNA Protects Against Sepsis-Induced Acute Respiratory Distress Syndrome. Front Immunol 2022, 13: 897487.

32. Chen X, Tang J, Shuai W, Meng J, Feng J, Han Z. Macrophage polarization and its role in the pathogenesis of acute lung injury/acute respiratory distress syndrome. Inflamm Res 2020, 69(9): 883-895.

33. Zaslona Z, Przybranowski S, Wilke C, van Rooijen N, Teitz-Tennenbaum S, Osterholzer JJ, et al. Resident alveolar macrophages suppress, whereas recruited monocytes promote, allergic lung inflammation in murine models of asthma. J Immunol 2014, 193(8): 4245-4253.

34. Han W, Tanjore H, Liu Y, Hunt RP, Gutor SS, Serezani APM, et al. Identification and Characterization of Alveolar and Recruited Lung Macrophages during Acute Lung Inflammation. J Immunol 2023, 210(11): 1827-1836.

35. Li Y, Li J, Yu Q, Ji L, Peng B. METTL14 regulates microglia/macrophage polarization and NLRP3 inflammasome activation after ischemic stroke by the KAT3B-STING axis. Neurobiol Dis 2023, 185: 106253.

36. Zheng Y, Li Y, Ran X, Wang D, Zheng X, Zhang M, et al. Mettl14 mediates the inflammatory response of macrophages in atherosclerosis through the NF-kappaB/IL-6 signaling pathway. Cell Mol Life Sci 2022, 79(6): 311.

37. Dang W, Tao Y, Xu X, Zhao H, Zou L, Li Y. The role of lung macrophages in acute respiratory distress syndrome. Inflamm Res 2022, 71(12): 1417-1432.

38. Wang X, Ding Y, Li R, Zhang R, Ge X, Gao R, et al. N(6)-methyladenosine of Spi2a attenuates inflammation and sepsis-associated myocardial dysfunction in mice. Nat Commun 2023, 14(1): 1185.

39. Mangan MSJ, Olhava EJ, Roush WR, Seidel HM, Glick GD, Latz E. Targeting the NLRP3 inflammasome in inflammatory diseases. Nat Rev Drug Discov 2018, 17(8): 588-606.

40. Wann SR, Chi PL, Huang WC, Cheng CC, Chang YT. Combination therapy of iPSC-derived conditioned medium with ceftriaxone alleviates bacteria-induced lung injury by targeting the NLRP3 inflammasome. J Cell Physiol 2022, 237(2): 1299-1314.

41. Xu Q, Ye Y, Wang Z, Zhu H, Li Y, Wang J, et al. NLRP3 Knockout Protects against Lung Injury Induced by Cerebral Ischemia-Reperfusion. Oxid Med Cell Longev 2022, 2022: 6260102.

42. Li H, Guan Y, Liang B, Ding P, Hou X, Wei W, et al. Therapeutic potential of MCC950, a specific inhibitor of NLRP3 inflammasome. Eur J Pharmacol 2022, 928: 175091.

1. Meyer NJ, Gattinoni L, Calfee CS. Acute respiratory distress syndrome. Lancet 2021, 398(10300): 622-637.

2. Gorman EA, O'Kane CM, McAuley DF. Acute respiratory distress syndrome in adults: diagnosis, outcomes, long-term sequelae, and management. Lancet 2022, 400(10358): 1157-1170.

3. Bos LDJ, Ware LB. Acute respiratory distress syndrome: causes, pathophysiology, and phenotypes. Lancet 2022, 400(10358): 1145-1156.

4. Gilroy DW, De Maeyer RPH, Tepper M, O'Brien A, Uddin M, Chen J, et al. Treating exuberant, non-resolving inflammation in the lung; Implications for acute respiratory distress syndrome and COVID-19. Pharmacol Ther 2021, 221: 107745.

5. Laffey JG, Matthay MA. Fifty Years of Research in ARDS. Cell-based Therapy for Acute Respiratory Distress Syndrome. Biology and Potential Therapeutic Value. Am J Respir Crit Care Med 2017, 196(3): 266-273.

6. Xian H, Liu Y, Rundberg Nilsson A, Gatchalian R, Crother TR, Tourtellotte WG, et al. Metformin inhibition of mitochondrial ATP and DNA synthesis abrogates NLRP3 inflammasome activation and pulmonary inflammation. Immunity 2021, 54(7): 1463-1477 e1411.

7. Paik S, Kim JK, Silwal P, Sasakawa C, Jo EK. An update on the regulatory mechanisms of NLRP3 inflammasome activation. Cell Mol Immunol 2021, 18(5): 1141-1160.

8. Kelley N, Jeltema D, Duan Y, He Y. The NLRP3 Inflammasome: An Overview of Mechanisms of Activation and Regulation. Int J Mol Sci 2019, 20(13).

9. Swanson KV, Deng M, Ting JP. The NLRP3 inflammasome: molecular activation and regulation to therapeutics. Nat Rev Immunol 2019, 19(8): 477-489.

10. Bauernfeind FG, Horvath G, Stutz A, Alnemri ES, MacDonald K, Speert D, et al. Cutting edge: NF-kappaB activating pattern recognition and cytokine receptors license NLRP3 inflammasome activation by regulating NLRP3 expression. J Immunol 2009, 183(2): 787-791.

11. Cornut M, Bourdonnay E, Henry T. Transcriptional Regulation of Inflammasomes. Int J Mol Sci 2020, 21(21).

12. Baker PJ, De Nardo D, Moghaddas F, Tran LS, Bachem A, Nguyen T, et al. Posttranslational Modification as a Critical Determinant of Cytoplasmic Innate Immune Recognition. Physiol Rev 2017, 97(3): 1165-1209.

13. Moretti J, Blander JM. Increasing complexity of NLRP3 inflammasome regulation. J Leukoc Biol 2021, 109(3): 561-571.

14. Liu Y, Yang D, Liu T, Chen J, Yu J, Yi P. N6-methyladenosine-mediated gene regulation and therapeutic implications. Trends Mol Med 2023, 29(6): 454-467.

15. Zaccara S, Ries RJ, Jaffrey SR. Reading, writing and erasing mRNA methylation. Nat Rev Mol Cell Biol 2019, 20(10): 608-624.

16. Zhao Y, Shi Y, Shen H, Xie W. m(6)A-binding proteins: the emerging crucial performers in epigenetics. J Hematol Oncol 2020, 13(1): 35.

17. Xu H, Lin C, Yang J, Chen X, Chen Y, Chen J, et al. The Role of N(6)-Methyladenosine in Inflammatory Diseases. Oxid Med Cell Longev 2022, 2022: 9744771.

18. Peng Z, Gong Y, Wang X, He W, Wu L, Zhang L, et al. METTL3-m(6)A-Rubicon axis inhibits autophagy in nonalcoholic fatty liver disease. Mol Ther 2022, 30(2): 932-946.

19. Zhang J, Song B, Zeng Y, Xu C, Gao L, Guo Y, et al. m6A modification in inflammatory bowel disease provides new insights into clinical applications. Biomed Pharmacother 2023, 159: 114298.

20. Winkler R, Gillis E, Lasman L, Safra M, Geula S, Soyris C, et al. m(6)A modification controls the innate immune response to infection by targeting type I interferons. Nat Immunol 2019, 20(2): 173-182.

21. Zhang H, Liu J, Zhou Y, Qu M, Wang Y, Guo K, et al. Neutrophil extracellular traps mediate m(6)A modification and regulates sepsis-associated acute lung injury by activating ferroptosis in alveolar epithelial cells. Int J Biol Sci 2022, 18(8): 3337-3357.

22. Jiao Y, Zhang T, Zhang C, Ji H, Tong X, Xia R, et al. Exosomal miR-30d-5p of neutrophils induces M1 macrophage polarization and primes macrophage pyroptosis in sepsis-related acute lung injury. Crit Care 2021, 25(1): 356.

23. Huang X, Zhang H, Guo X, Zhu Z, Cai H, Kong X. Insulin-like growth factor 2 mRNA-binding protein 1 (IGF2BP1) in cancer. J Hematol Oncol 2018, 11(1): 88.

24. Huang H, Weng H, Chen J. m(6)A Modification in Coding and Non-coding RNAs: Roles and Therapeutic Implications in Cancer. Cancer Cell 2020, 37(3): 270-288.

25. Zhu X, Tang H, Yang M, Yin K. N6-methyladenosine in macrophage function: a novel target for metabolic diseases. Trends Endocrinol Metab 2023, 34(2): 66-84.

26. Kumari R, Ranjan P, Suleiman ZG, Goswami SK, Li J, Prasad R, et al. mRNA modifications in cardiovascular biology and disease: with a focus on m6A modification. Cardiovasc Res 2022, 118(7): 1680-1692.

27. Luo J, Xu T, Sun K. N6-Methyladenosine RNA Modification in Inflammation: Roles, Mechanisms, and Applications. Front Cell Dev Biol 2021, 9: 670711.

28. Weng H, Huang H, Wu H, Qin X, Zhao BS, Dong L, et al. METTL14 Inhibits Hematopoietic Stem/Progenitor Differentiation and Promotes Leukemogenesis via mRNA m(6)A Modification. Cell Stem Cell 2018, 22(2): 191-205 e199.

29. Zhang H, Wu D, Wang Y, Guo K, Spencer CB, Ortoga L, et al. METTL3-mediated N6-methyladenosine exacerbates ferroptosis via m6A-IGF2BP2-dependent mitochondrial metabolic reprogramming in sepsis-induced acute lung injury. Clin Transl Med 2023, 13(9): e1389.

30. Qu M, Chen Z, Qiu Z, Nan K, Wang Y, Shi Y, et al. Neutrophil extracellular traps-triggered impaired autophagic flux via METTL3 underlies sepsis-associated acute lung injury. Cell Death Discov 2022, 8(1): 375.

31. Chen Y, Wu Y, Zhu L, Chen C, Xu S, Tang D, et al. METTL3-Mediated N6-Methyladenosine Modification of Trim59 mRNA Protects Against Sepsis-Induced Acute Respiratory Distress Syndrome. Front Immunol 2022, 13: 897487.

32. Chen X, Tang J, Shuai W, Meng J, Feng J, Han Z. Macrophage polarization and its role in the pathogenesis of acute lung injury/acute respiratory distress syndrome. Inflamm Res 2020, 69(9): 883-895.

33. Zaslona Z, Przybranowski S, Wilke C, van Rooijen N, Teitz-Tennenbaum S, Osterholzer JJ, et al. Resident alveolar macrophages suppress, whereas recruited monocytes promote, allergic lung inflammation in murine models of asthma. J Immunol 2014, 193(8): 4245-4253.

34. Han W, Tanjore H, Liu Y, Hunt RP, Gutor SS, Serezani APM, et al. Identification and Characterization of Alveolar and Recruited Lung Macrophages during Acute Lung Inflammation. J Immunol 2023, 210(11): 1827-1836.

35. Li Y, Li J, Yu Q, Ji L, Peng B. METTL14 regulates microglia/macrophage polarization and NLRP3 inflammasome activation after ischemic stroke by the KAT3B-STING axis. Neurobiol Dis 2023, 185: 106253.

36. Zheng Y, Li Y, Ran X, Wang D, Zheng X, Zhang M, et al. Mettl14 mediates the inflammatory response of macrophages in atherosclerosis through the NF-kappaB/IL-6 signaling pathway. Cell Mol Life Sci 2022, 79(6): 311.

37. Dang W, Tao Y, Xu X, Zhao H, Zou L, Li Y. The role of lung macrophages in acute respiratory distress syndrome. Inflamm Res 2022, 71(12): 1417-1432.

38. Wang X, Ding Y, Li R, Zhang R, Ge X, Gao R, et al. N(6)-methyladenosine of Spi2a attenuates inflammation and sepsis-associated myocardial dysfunction in mice. Nat Commun 2023, 14(1): 1185.

39. Mangan MSJ, Olhava EJ, Roush WR, Seidel HM, Glick GD, Latz E. Targeting the NLRP3 inflammasome in inflammatory diseases. Nat Rev Drug Discov 2018, 17(8): 588-606.

40. Wann SR, Chi PL, Huang WC, Cheng CC, Chang YT. Combination therapy of iPSC-derived conditioned medium with ceftriaxone alleviates bacteria-induced lung injury by targeting the NLRP3 inflammasome. J Cell Physiol 2022, 237(2): 1299-1314.

41. Xu Q, Ye Y, Wang Z, Zhu H, Li Y, Wang J, et al. NLRP3 Knockout Protects against Lung Injury Induced by Cerebral Ischemia-Reperfusion. Oxid Med Cell Longev 2022, 2022: 6260102.

42. Bakhshi S, Shamsi S. MCC950 in the treatment of NLRP3-mediated inflammatory diseases: Latest evidence and therapeutic outcomes. Int Immunopharmacol 2022, 106: 108595.

43. Li H, Guan Y, Liang B, Ding P, Hou X, Wei W, et al. Therapeutic potential of MCC950, a specific inhibitor of NLRP3 inflammasome. Eur J Pharmacol 2022, 928: 175091.

1. Meyer NJ, Gattinoni L, Calfee CS. Acute respiratory distress syndrome. Lancet 2021, 398(10300): 622-637.

2. Gorman EA, O'Kane CM, McAuley DF. Acute respiratory distress syndrome in adults: diagnosis, outcomes, long-term sequelae, and management. Lancet 2022, 400(10358): 1157-1170.

3. Bos LDJ, Ware LB. Acute respiratory distress syndrome: causes, pathophysiology, and phenotypes. Lancet 2022, 400(10358): 1145-1156.

4. Gilroy DW, De Maeyer RPH, Tepper M, O'Brien A, Uddin M, Chen J, et al. Treating exuberant, non-resolving inflammation in the lung; Implications for acute respiratory distress syndrome and COVID-19. Pharmacol Ther 2021, 221: 107745.

5. Laffey JG, Matthay MA. Fifty Years of Research in ARDS. Cell-based Therapy for Acute Respiratory Distress Syndrome. Biology and Potential Therapeutic Value. Am J Respir Crit Care Med 2017, 196(3): 266-273.

6. Xian H, Liu Y, Rundberg Nilsson A, Gatchalian R, Crother TR, Tourtellotte WG, et al. Metformin inhibition of mitochondrial ATP and DNA synthesis abrogates NLRP3 inflammasome activation and pulmonary inflammation. Immunity 2021, 54(7): 1463-1477 e1411.

7. Paik S, Kim JK, Silwal P, Sasakawa C, Jo EK. An update on the regulatory mechanisms of NLRP3 inflammasome activation. Cell Mol Immunol 2021, 18(5): 1141-1160.

8. Kelley N, Jeltema D, Duan Y, He Y. The NLRP3 Inflammasome: An Overview of Mechanisms of Activation and Regulation. Int J Mol Sci 2019, 20(13).

9. Swanson KV, Deng M, Ting JP. The NLRP3 inflammasome: molecular activation and regulation to therapeutics. Nat Rev Immunol 2019, 19(8): 477-489.

10. Bauernfeind FG, Horvath G, Stutz A, Alnemri ES, MacDonald K, Speert D, et al. Cutting edge: NF-kappaB activating pattern recognition and cytokine receptors license NLRP3 inflammasome activation by regulating NLRP3 expression. J Immunol 2009, 183(2): 787-791.

11. Cornut M, Bourdonnay E, Henry T. Transcriptional Regulation of Inflammasomes. Int J Mol Sci 2020, 21(21).

12. Baker PJ, De Nardo D, Moghaddas F, Tran LS, Bachem A, Nguyen T, et al. Posttranslational Modification as a Critical Determinant of Cytoplasmic Innate Immune Recognition. Physiol Rev 2017, 97(3): 1165-1209.

13. Moretti J, Blander JM. Increasing complexity of NLRP3 inflammasome regulation. J Leukoc Biol 2021, 109(3): 561-571.

14. Liu Y, Yang D, Liu T, Chen J, Yu J, Yi P. N6-methyladenosine-mediated gene regulation and therapeutic implications. Trends Mol Med 2023, 29(6): 454-467.

15. Zaccara S, Ries RJ, Jaffrey SR. Reading, writing and erasing mRNA methylation. Nat Rev Mol Cell Biol 2019, 20(10): 608-624.

16. Zhao Y, Shi Y, Shen H, Xie W. m(6)A-binding proteins: the emerging crucial performers in epigenetics. J Hematol Oncol 2020, 13(1): 35.

17. Xu H, Lin C, Yang J, Chen X, Chen Y, Chen J, et al. The Role of N(6)-Methyladenosine in Inflammatory Diseases. Oxid Med Cell Longev 2022, 2022: 9744771.

18. Peng Z, Gong Y, Wang X, He W, Wu L, Zhang L, et al. METTL3-m(6)A-Rubicon axis inhibits autophagy in nonalcoholic fatty liver disease. Mol Ther 2022, 30(2): 932-946.

19. Zhang J, Song B, Zeng Y, Xu C, Gao L, Guo Y, et al. m6A modification in inflammatory bowel disease provides new insights into clinical applications. Biomed Pharmacother 2023, 159: 114298.

20. Winkler R, Gillis E, Lasman L, Safra M, Geula S, Soyris C, et al. m(6)A modification controls the innate immune response to infection by targeting type I interferons. Nat Immunol 2019, 20(2): 173-182.

21. Zhang H, Liu J, Zhou Y, Qu M, Wang Y, Guo K, et al. Neutrophil extracellular traps mediate m(6)A modification and regulates sepsis-associated acute lung injury by activating ferroptosis in alveolar epithelial cells. Int J Biol Sci 2022, 18(8): 3337-3357.

22. Jiao Y, Zhang T, Zhang C, Ji H, Tong X, Xia R, et al. Exosomal miR-30d-5p of neutrophils induces M1 macrophage polarization and primes macrophage pyroptosis in sepsis-related acute lung injury. Crit Care 2021, 25(1): 356.

23. Huang X, Zhang H, Guo X, Zhu Z, Cai H, Kong X. Insulin-like growth factor 2 mRNA-binding protein 1 (IGF2BP1) in cancer. J Hematol Oncol 2018, 11(1): 88.

24. Huang H, Weng H, Chen J. m(6)A Modification in Coding and Non-coding RNAs: Roles and Therapeutic Implications in Cancer. Cancer Cell 2020, 37(3): 270-288.

25. Zhu X, Tang H, Yang M, Yin K. N6-methyladenosine in macrophage function: a novel target for metabolic diseases. Trends Endocrinol Metab 2023, 34(2): 66-84.

26. Kumari R, Ranjan P, Suleiman ZG, Goswami SK, Li J, Prasad R, et al. mRNA modifications in cardiovascular biology and disease: with a focus on m6A modification. Cardiovasc Res 2022, 118(7): 1680-1692.

27. Luo J, Xu T, Sun K. N6-Methyladenosine RNA Modification in Inflammation: Roles, Mechanisms, and Applications. Front Cell Dev Biol 2021, 9: 670711.

28. Weng H, Huang H, Wu H, Qin X, Zhao BS, Dong L, et al. METTL14 Inhibits Hematopoietic Stem/Progenitor Differentiation and Promotes Leukemogenesis via mRNA m(6)A Modification. Cell Stem Cell 2018, 22(2): 191-205 e199.

29. Zhang H, Wu D, Wang Y, Guo K, Spencer CB, Ortoga L, et al. METTL3-mediated N6-methyladenosine exacerbates ferroptosis via m6A-IGF2BP2-dependent mitochondrial metabolic reprogramming in sepsis-induced acute lung injury. Clin Transl Med 2023, 13(9): e1389.

30. Qu M, Chen Z, Qiu Z, Nan K, Wang Y, Shi Y, et al. Neutrophil extracellular traps-triggered impaired autophagic flux via METTL3 underlies sepsis-associated acute lung injury. Cell Death Discov 2022, 8(1): 375.

31. Chen Y, Wu Y, Zhu L, Chen C, Xu S, Tang D, et al. METTL3-Mediated N6-Methyladenosine Modification of Trim59 mRNA Protects Against Sepsis-Induced Acute Respiratory Distress Syndrome. Front Immunol 2022, 13: 897487.

32. Chen X, Tang J, Shuai W, Meng J, Feng J, Han Z. Macrophage polarization and its role in the pathogenesis of acute lung injury/acute respiratory distress syndrome. Inflamm Res 2020, 69(9): 883-895.

33. Zaslona Z, Przybranowski S, Wilke C, van Rooijen N, Teitz-Tennenbaum S, Osterholzer JJ, et al. Resident alveolar macrophages suppress, whereas recruited monocytes promote, allergic lung inflammation in murine models of asthma. J Immunol 2014, 193(8): 4245-4253.

34. Han W, Tanjore H, Liu Y, Hunt RP, Gutor SS, Serezani APM, et al. Identification and Characterization of Alveolar and Recruited Lung Macrophages during Acute Lung Inflammation. J Immunol 2023, 210(11): 1827-1836.

35. Li Y, Li J, Yu Q, Ji L, Peng B. METTL14 regulates microglia/macrophage polarization and NLRP3 inflammasome activation after ischemic stroke by the KAT3B-STING axis. Neurobiol Dis 2023, 185: 106253.

36. Zheng Y, Li Y, Ran X, Wang D, Zheng X, Zhang M, et al. Mettl14 mediates the inflammatory response of macrophages in atherosclerosis through the NF-kappaB/IL-6 signaling pathway. Cell Mol Life Sci 2022, 79(6): 311.

37. Dang W, Tao Y, Xu X, Zhao H, Zou L, Li Y. The role of lung macrophages in acute respiratory distress syndrome. Inflamm Res 2022, 71(12): 1417-1432.

38. Wang X, Ding Y, Li R, Zhang R, Ge X, Gao R, et al. N(6)-methyladenosine of Spi2a attenuates inflammation and sepsis-associated myocardial dysfunction in mice. Nat Commun 2023, 14(1): 1185.

39. Mangan MSJ, Olhava EJ, Roush WR, Seidel HM, Glick GD, Latz E. Targeting the NLRP3 inflammasome in inflammatory diseases. Nat Rev Drug Discov 2018, 17(8): 588-606.

40. Wann SR, Chi PL, Huang WC, Cheng CC, Chang YT. Combination therapy of iPSC-derived conditioned medium with ceftriaxone alleviates bacteria-induced lung injury by targeting the NLRP3 inflammasome. J Cell Physiol 2022, 237(2): 1299-1314.

41. Xu Q, Ye Y, Wang Z, Zhu H, Li Y, Wang J, et al. NLRP3 Knockout Protects against Lung Injury Induced by Cerebral Ischemia-Reperfusion. Oxid Med Cell Longev 2022, 2022: 6260102.

42. Bakhshi S, Shamsi S. MCC950 in the treatment of NLRP3-mediated inflammatory diseases: Latest evidence and therapeutic outcomes. Int Immunopharmacol 2022, 106: 108595.

43. Li H, Guan Y, Liang B, Ding P, Hou X, Wei W, et al. Therapeutic potential of MCC950, a specific inhibitor of NLRP3 inflammasome. Eur J Pharmacol 2022, 928: 175091.

44. Meng L, Lin H, Huang X, Weng J, Peng F, Wu S. METTL14 suppresses pyroptosis and diabetic cardiomyopathy by downregulating TINCR lncRNA. Cell Death Dis 2022, 13(1): 38.

1. Meyer NJ, Gattinoni L, Calfee CS. Acute respiratory distress syndrome. Lancet 2021, 398(10300): 622-637.

2. Gorman EA, O'Kane CM, McAuley DF. Acute respiratory distress syndrome in adults: diagnosis, outcomes, long-term sequelae, and management. Lancet 2022, 400(10358): 1157-1170.

3. Bos LDJ, Ware LB. Acute respiratory distress syndrome: causes, pathophysiology, and phenotypes. Lancet 2022, 400(10358): 1145-1156.

4. Gilroy DW, De Maeyer RPH, Tepper M, O'Brien A, Uddin M, Chen J, et al. Treating exuberant, non-resolving inflammation in the lung; Implications for acute respiratory distress syndrome and COVID-19. Pharmacol Ther 2021, 221: 107745.

5. Laffey JG, Matthay MA. Fifty Years of Research in ARDS. Cell-based Therapy for Acute Respiratory Distress Syndrome. Biology and Potential Therapeutic Value. Am J Respir Crit Care Med 2017, 196(3): 266-273.

6. Xian H, Liu Y, Rundberg Nilsson A, Gatchalian R, Crother TR, Tourtellotte WG, et al. Metformin inhibition of mitochondrial ATP and DNA synthesis abrogates NLRP3 inflammasome activation and pulmonary inflammation. Immunity 2021, 54(7): 1463-1477 e1411.

7. Paik S, Kim JK, Silwal P, Sasakawa C, Jo EK. An update on the regulatory mechanisms of NLRP3 inflammasome activation. Cell Mol Immunol 2021, 18(5): 1141-1160.

8. Kelley N, Jeltema D, Duan Y, He Y. The NLRP3 Inflammasome: An Overview of Mechanisms of Activation and Regulation. Int J Mol Sci 2019, 20(13).

9. Swanson KV, Deng M, Ting JP. The NLRP3 inflammasome: molecular activation and regulation to therapeutics. Nat Rev Immunol 2019, 19(8): 477-489.

10. Bauernfeind FG, Horvath G, Stutz A, Alnemri ES, MacDonald K, Speert D, et al. Cutting edge: NF-kappaB activating pattern recognition and cytokine receptors license NLRP3 inflammasome activation by regulating NLRP3 expression. J Immunol 2009, 183(2): 787-791.

11. Cornut M, Bourdonnay E, Henry T. Transcriptional Regulation of Inflammasomes. Int J Mol Sci 2020, 21(21).

12. Baker PJ, De Nardo D, Moghaddas F, Tran LS, Bachem A, Nguyen T, et al. Posttranslational Modification as a Critical Determinant of Cytoplasmic Innate Immune Recognition. Physiol Rev 2017, 97(3): 1165-1209.

13. Moretti J, Blander JM. Increasing complexity of NLRP3 inflammasome regulation. J Leukoc Biol 2021, 109(3): 561-571.

14. Liu Y, Yang D, Liu T, Chen J, Yu J, Yi P. N6-methyladenosine-mediated gene regulation and therapeutic implications. Trends Mol Med 2023, 29(6): 454-467.

15. Zaccara S, Ries RJ, Jaffrey SR. Reading, writing and erasing mRNA methylation. Nat Rev Mol Cell Biol 2019, 20(10): 608-624.

16. Zhao Y, Shi Y, Shen H, Xie W. m(6)A-binding proteins: the emerging crucial performers in epigenetics. J Hematol Oncol 2020, 13(1): 35.

17. Xu H, Lin C, Yang J, Chen X, Chen Y, Chen J, et al. The Role of N(6)-Methyladenosine in Inflammatory Diseases. Oxid Med Cell Longev 2022, 2022: 9744771.

18. Peng Z, Gong Y, Wang X, He W, Wu L, Zhang L, et al. METTL3-m(6)A-Rubicon axis inhibits autophagy in nonalcoholic fatty liver disease. Mol Ther 2022, 30(2): 932-946.

19. Zhang J, Song B, Zeng Y, Xu C, Gao L, Guo Y, et al. m6A modification in inflammatory bowel disease provides new insights into clinical applications. Biomed Pharmacother 2023, 159: 114298.

20. Winkler R, Gillis E, Lasman L, Safra M, Geula S, Soyris C, et al. m(6)A modification controls the innate immune response to infection by targeting type I interferons. Nat Immunol 2019, 20(2): 173-182.

21. Zhang H, Liu J, Zhou Y, Qu M, Wang Y, Guo K, et al. Neutrophil extracellular traps mediate m(6)A modification and regulates sepsis-associated acute lung injury by activating ferroptosis in alveolar epithelial cells. Int J Biol Sci 2022, 18(8): 3337-3357.

22. Jiao Y, Zhang T, Zhang C, Ji H, Tong X, Xia R, et al. Exosomal miR-30d-5p of neutrophils induces M1 macrophage polarization and primes macrophage pyroptosis in sepsis-related acute lung injury. Crit Care 2021, 25(1): 356.

23. Huang X, Zhang H, Guo X, Zhu Z, Cai H, Kong X. Insulin-like growth factor 2 mRNA-binding protein 1 (IGF2BP1) in cancer. J Hematol Oncol 2018, 11(1): 88.

24. Huang H, Weng H, Chen J. m(6)A Modification in Coding and Non-coding RNAs: Roles and Therapeutic Implications in Cancer. Cancer Cell 2020, 37(3): 270-288.

25. Zhu X, Tang H, Yang M, Yin K. N6-methyladenosine in macrophage function: a novel target for metabolic diseases. Trends Endocrinol Metab 2023, 34(2): 66-84.

27. Luo J, Xu T, Sun K. N6-Methyladenosine RNA Modification in Inflammation: Roles, Mechanisms, and Applications. Front Cell Dev Biol 2021, 9: 670711.

29. Zhang H, Wu D, Wang Y, Guo K, Spencer CB, Ortoga L, et al. METTL3-mediated N6-methyladenosine exacerbates ferroptosis via m6A-IGF2BP2-dependent mitochondrial metabolic reprogramming in sepsis-induced acute lung injury. Clin Transl Med 2023, 13(9): e1389.

30. Qu M, Chen Z, Qiu Z, Nan K, Wang Y, Shi Y, et al. Neutrophil extracellular traps-triggered impaired autophagic flux via METTL3 underlies sepsis-associated acute lung injury. Cell Death Discov 2022, 8(1): 375.

31. Chen Y, Wu Y, Zhu L, Chen C, Xu S, Tang D, et al. METTL3-Mediated N6-Methyladenosine Modification of Trim59 mRNA Protects Against Sepsis-Induced Acute Respiratory Distress Syndrome. Front Immunol 2022, 13: 897487.

32. Chen X, Tang J, Shuai W, Meng J, Feng J, Han Z. Macrophage polarization and its role in the pathogenesis of acute lung injury/acute respiratory distress syndrome. Inflamm Res 2020, 69(9): 883-895.

33. Zaslona Z, Przybranowski S, Wilke C, van Rooijen N, Teitz-Tennenbaum S, Osterholzer JJ, et al. Resident alveolar macrophages suppress, whereas recruited monocytes promote, allergic lung inflammation in murine models of asthma. J Immunol 2014, 193(8): 4245-4253.

34. Han W, Tanjore H, Liu Y, Hunt RP, Gutor SS, Serezani APM, et al. Identification and Characterization of Alveolar and Recruited Lung Macrophages during Acute Lung Inflammation. J Immunol 2023, 210(11): 1827-1836.

35. Li Y, Li J, Yu Q, Ji L, Peng B. METTL14 regulates microglia/macrophage polarization and NLRP3 inflammasome activation after ischemic stroke by the KAT3B-STING axis. Neurobiol Dis 2023, 185: 106253.

36. Zheng Y, Li Y, Ran X, Wang D, Zheng X, Zhang M, et al. Mettl14 mediates the inflammatory response of macrophages in atherosclerosis through the NF-kappaB/IL-6 signaling pathway. Cell Mol Life Sci 2022, 79(6): 311.

37. Dang W, Tao Y, Xu X, Zhao H, Zou L, Li Y. The role of lung macrophages in acute respiratory distress syndrome. Inflamm Res 2022, 71(12): 1417-1432.

38. Wang X, Ding Y, Li R, Zhang R, Ge X, Gao R, et al. N(6)-methyladenosine of Spi2a attenuates inflammation and sepsis-associated myocardial dysfunction in mice. Nat Commun 2023, 14(1): 1185.

39. Mangan MSJ, Olhava EJ, Roush WR, Seidel HM, Glick GD, Latz E. Targeting the NLRP3 inflammasome in inflammatory diseases. Nat Rev Drug Discov 2018, 17(8): 588-606.

40. Wann SR, Chi PL, Huang WC, Cheng CC, Chang YT. Combination therapy of iPSC-derived conditioned medium with ceftriaxone alleviates bacteria-induced lung injury by targeting the NLRP3 inflammasome. J Cell Physiol 2022, 237(2): 1299-1314.

41. Xu Q, Ye Y, Wang Z, Zhu H, Li Y, Wang J, et al. NLRP3 Knockout Protects against Lung Injury Induced by Cerebral Ischemia-Reperfusion. Oxid Med Cell Longev 2022, 2022: 6260102.

42. Bakhshi S, Shamsi S. MCC950 in the treatment of NLRP3-mediated inflammatory diseases: Latest evidence and therapeutic outcomes. Int Immunopharmacol 2022, 106: 108595.

43. Li H, Guan Y, Liang B, Ding P, Hou X, Wei W, et al. Therapeutic potential of MCC950, a specific inhibitor of NLRP3 inflammasome. Eur J Pharmacol 2022, 928: 175091.

44. Meng L, Lin H, Huang X, Weng J, Peng F, Wu S. METTL14 suppresses pyroptosis and diabetic cardiomyopathy by downregulating TINCR lncRNA. Cell Death Dis 2022, 13(1): 38.

45. Yuan X, Li T, Shi L, Miao J, Guo Y, Chen Y. Human umbilical cord mesenchymal stem cells deliver exogenous miR-26a-5p via exosomes to inhibit nucleus pulposus cell pyroptosis through METTL14/NLRP3. Mol Med 2021, 27(1): 91.

1. Meyer NJ, Gattinoni L, Calfee CS. Acute respiratory distress syndrome. Lancet 2021, 398(10300): 622-637.

2. Gorman EA, O'Kane CM, McAuley DF. Acute respiratory distress syndrome in adults: diagnosis, outcomes, long-term sequelae, and management. Lancet 2022, 400(10358): 1157-1170.

3. Bos LDJ, Ware LB. Acute respiratory distress syndrome: causes, pathophysiology, and phenotypes. Lancet 2022, 400(10358): 1145-1156.

4. Gilroy DW, De Maeyer RPH, Tepper M, O'Brien A, Uddin M, Chen J, et al. Treating exuberant, non-resolving inflammation in the lung; Implications for acute respiratory distress syndrome and COVID-19. Pharmacol Ther 2021, 221: 107745.

5. Laffey JG, Matthay MA. Fifty Years of Research in ARDS. Cell-based Therapy for Acute Respiratory Distress Syndrome. Biology and Potential Therapeutic Value. Am J Respir Crit Care Med 2017, 196(3): 266-273.

6. Xian H, Liu Y, Rundberg Nilsson A, Gatchalian R, Crother TR, Tourtellotte WG, et al. Metformin inhibition of mitochondrial ATP and DNA synthesis abrogates NLRP3 inflammasome activation and pulmonary inflammation. Immunity 2021, 54(7): 1463-1477 e1411.

7. Paik S, Kim JK, Silwal P, Sasakawa C, Jo EK. An update on the regulatory mechanisms of NLRP3 inflammasome activation. Cell Mol Immunol 2021, 18(5): 1141-1160.

8. Kelley N, Jeltema D, Duan Y, He Y. The NLRP3 Inflammasome: An Overview of Mechanisms of Activation and Regulation. Int J Mol Sci 2019, 20(13).

9. Swanson KV, Deng M, Ting JP. The NLRP3 inflammasome: molecular activation and regulation to therapeutics. Nat Rev Immunol 2019, 19(8): 477-489.

10. Bauernfeind FG, Horvath G, Stutz A, Alnemri ES, MacDonald K, Speert D, et al. Cutting edge: NF-kappaB activating pattern recognition and cytokine receptors license NLRP3 inflammasome activation by regulating NLRP3 expression. J Immunol 2009, 183(2): 787-791.

11. Cornut M, Bourdonnay E, Henry T. Transcriptional Regulation of Inflammasomes. Int J Mol Sci 2020, 21(21).

12. Baker PJ, De Nardo D, Moghaddas F, Tran LS, Bachem A, Nguyen T, et al. Posttranslational Modification as a Critical Determinant of Cytoplasmic Innate Immune Recognition. Physiol Rev 2017, 97(3): 1165-1209.

13. Moretti J, Blander JM. Increasing complexity of NLRP3 inflammasome regulation. J Leukoc Biol 2021, 109(3): 561-571.

14. Liu Y, Yang D, Liu T, Chen J, Yu J, Yi P. N6-methyladenosine-mediated gene regulation and therapeutic implications. Trends Mol Med 2023, 29(6): 454-467.

15. Zaccara S, Ries RJ, Jaffrey SR. Reading, writing and erasing mRNA methylation. Nat Rev Mol Cell Biol 2019, 20(10): 608-624.

16. Zhao Y, Shi Y, Shen H, Xie W. m(6)A-binding proteins: the emerging crucial performers in epigenetics. J Hematol Oncol 2020, 13(1): 35.

17. Xu H, Lin C, Yang J, Chen X, Chen Y, Chen J, et al. The Role of N(6)-Methyladenosine in Inflammatory Diseases. Oxid Med Cell Longev 2022, 2022: 9744771.

18. Peng Z, Gong Y, Wang X, He W, Wu L, Zhang L, et al. METTL3-m(6)A-Rubicon axis inhibits autophagy in nonalcoholic fatty liver disease. Mol Ther 2022, 30(2): 932-946.

19. Zhang J, Song B, Zeng Y, Xu C, Gao L, Guo Y, et al. m6A modification in inflammatory bowel disease provides new insights into clinical applications. Biomed Pharmacother 2023, 159: 114298.

20. Winkler R, Gillis E, Lasman L, Safra M, Geula S, Soyris C, et al. m(6)A modification controls the innate immune response to infection by targeting type I interferons. Nat Immunol 2019, 20(2): 173-182.

21. Zhang H, Liu J, Zhou Y, Qu M, Wang Y, Guo K, et al. Neutrophil extracellular traps mediate m(6)A modification and regulates sepsis-associated acute lung injury by activating ferroptosis in alveolar epithelial cells. Int J Biol Sci 2022, 18(8): 3337-3357.

22. Jiao Y, Zhang T, Zhang C, Ji H, Tong X, Xia R, et al. Exosomal miR-30d-5p of neutrophils induces M1 macrophage polarization and primes macrophage pyroptosis in sepsis-related acute lung injury. Crit Care 2021, 25(1): 356.

23. Huang X, Zhang H, Guo X, Zhu Z, Cai H, Kong X. Insulin-like growth factor 2 mRNA-binding protein 1 (IGF2BP1) in cancer. J Hematol Oncol 2018, 11(1): 88.

24. Huang H, Weng H, Chen J. m(6)A Modification in Coding and Non-coding RNAs: Roles and Therapeutic Implications in Cancer. Cancer Cell 2020, 37(3): 270-288.

25. Zhu X, Tang H, Yang M, Yin K. N6-methyladenosine in macrophage function: a novel target for metabolic diseases. Trends Endocrinol Metab 2023, 34(2): 66-84.

26. Kumari R, Ranjan P, Suleiman ZG, Goswami SK, Li J, Prasad R, et al. mRNA modifications in cardiovascular biology and disease: with a focus on m6A modification. Cardiovasc Res 2022, 118(7): 1680-1692.

27. Luo J, Xu T, Sun K. N6-Methyladenosine RNA Modification in Inflammation: Roles, Mechanisms, and Applications. Front Cell Dev Biol 2021, 9: 670711.

28. Weng H, Huang H, Wu H, Qin X, Zhao BS, Dong L, et al. METTL14 Inhibits Hematopoietic Stem/Progenitor Differentiation and Promotes Leukemogenesis via mRNA m(6)A Modification. Cell Stem Cell 2018, 22(2): 191-205 e199.

29. Zhang H, Wu D, Wang Y, Guo K, Spencer CB, Ortoga L, et al. METTL3-mediated N6-methyladenosine exacerbates ferroptosis via m6A-IGF2BP2-dependent mitochondrial metabolic reprogramming in sepsis-induced acute lung injury. Clin Transl Med 2023, 13(9): e1389.

30. Qu M, Chen Z, Qiu Z, Nan K, Wang Y, Shi Y, et al. Neutrophil extracellular traps-triggered impaired autophagic flux via METTL3 underlies sepsis-associated acute lung injury. Cell Death Discov 2022, 8(1): 375.

31. Chen Y, Wu Y, Zhu L, Chen C, Xu S, Tang D, et al. METTL3-Mediated N6-Methyladenosine Modification of Trim59 mRNA Protects Against Sepsis-Induced Acute Respiratory Distress Syndrome. Front Immunol 2022, 13: 897487.

32. Chen X, Tang J, Shuai W, Meng J, Feng J, Han Z. Macrophage polarization and its role in the pathogenesis of acute lung injury/acute respiratory distress syndrome. Inflamm Res 2020, 69(9): 883-895.

33. Zaslona Z, Przybranowski S, Wilke C, van Rooijen N, Teitz-Tennenbaum S, Osterholzer JJ, et al. Resident alveolar macrophages suppress, whereas recruited monocytes promote, allergic lung inflammation in murine models of asthma. J Immunol 2014, 193(8): 4245-4253.

34. Han W, Tanjore H, Liu Y, Hunt RP, Gutor SS, Serezani APM, et al. Identification and Characterization of Alveolar and Recruited Lung Macrophages during Acute Lung Inflammation. J Immunol 2023, 210(11): 1827-1836.

35. Li Y, Li J, Yu Q, Ji L, Peng B. METTL14 regulates microglia/macrophage polarization and NLRP3 inflammasome activation after ischemic stroke by the KAT3B-STING axis. Neurobiol Dis 2023, 185: 106253.

36. Zheng Y, Li Y, Ran X, Wang D, Zheng X, Zhang M, et al. Mettl14 mediates the inflammatory response of macrophages in atherosclerosis through the NF-kappaB/IL-6 signaling pathway. Cell Mol Life Sci 2022, 79(6): 311.

37. Dang W, Tao Y, Xu X, Zhao H, Zou L, Li Y. The role of lung macrophages in acute respiratory distress syndrome. Inflamm Res 2022, 71(12): 1417-1432.

38. Wang X, Ding Y, Li R, Zhang R, Ge X, Gao R, et al. N(6)-methyladenosine of Spi2a attenuates inflammation and sepsis-associated myocardial dysfunction in mice. Nat Commun 2023, 14(1): 1185.

39. Mangan MSJ, Olhava EJ, Roush WR, Seidel HM, Glick GD, Latz E. Targeting the NLRP3 inflammasome in inflammatory diseases. Nat Rev Drug Discov 2018, 17(8): 588-606.

40. Wann SR, Chi PL, Huang WC, Cheng CC, Chang YT. Combination therapy of iPSC-derived conditioned medium with ceftriaxone alleviates bacteria-induced lung injury by targeting the NLRP3 inflammasome. J Cell Physiol 2022, 237(2): 1299-1314.

41. Xu Q, Ye Y, Wang Z, Zhu H, Li Y, Wang J, et al. NLRP3 Knockout Protects against Lung Injury Induced by Cerebral Ischemia-Reperfusion. Oxid Med Cell Longev 2022, 2022: 6260102.

42. Bakhshi S, Shamsi S. MCC950 in the treatment of NLRP3-mediated inflammatory diseases: Latest evidence and therapeutic outcomes. Int Immunopharmacol 2022, 106: 108595.

43. Li H, Guan Y, Liang B, Ding P, Hou X, Wei W, et al. Therapeutic potential of MCC950, a specific inhibitor of NLRP3 inflammasome. Eur J Pharmacol 2022, 928: 175091.

45. Yuan X, Li T, Shi L, Miao J, Guo Y, Chen Y. Human umbilical cord mesenchymal stem cells deliver exogenous miR-26a-5p via exosomes to inhibit nucleus pulposus cell pyroptosis through METTL14/NLRP3. Mol Med 2021, 27(1): 91.

1. Meyer NJ, Gattinoni L, Calfee CS. Acute respiratory distress syndrome. Lancet 2021, 398(10300): 622-637.

2. Gorman EA, O'Kane CM, McAuley DF. Acute respiratory distress syndrome in adults: diagnosis, outcomes, long-term sequelae, and management. Lancet 2022, 400(10358): 1157-1170.

3. Bos LDJ, Ware LB. Acute respiratory distress syndrome: causes, pathophysiology, and phenotypes. Lancet 2022, 400(10358): 1145-1156.

4. Gilroy DW, De Maeyer RPH, Tepper M, O'Brien A, Uddin M, Chen J, et al. Treating exuberant, non-resolving inflammation in the lung; Implications for acute respiratory distress syndrome and COVID-19. Pharmacol Ther 2021, 221: 107745.

5. Laffey JG, Matthay MA. Fifty Years of Research in ARDS. Cell-based Therapy for Acute Respiratory Distress Syndrome. Biology and Potential Therapeutic Value. Am J Respir Crit Care Med 2017, 196(3): 266-273.

6. Xian H, Liu Y, Rundberg Nilsson A, Gatchalian R, Crother TR, Tourtellotte WG, et al. Metformin inhibition of mitochondrial ATP and DNA synthesis abrogates NLRP3 inflammasome activation and pulmonary inflammation. Immunity 2021, 54(7): 1463-1477 e1411.

7. Paik S, Kim JK, Silwal P, Sasakawa C, Jo EK. An update on the regulatory mechanisms of NLRP3 inflammasome activation. Cell Mol Immunol 2021, 18(5): 1141-1160.

8. Kelley N, Jeltema D, Duan Y, He Y. The NLRP3 Inflammasome: An Overview of Mechanisms of Activation and Regulation. Int J Mol Sci 2019, 20(13).

9. Swanson KV, Deng M, Ting JP. The NLRP3 inflammasome: molecular activation and regulation to therapeutics. Nat Rev Immunol 2019, 19(8): 477-489.

10. Bauernfeind FG, Horvath G, Stutz A, Alnemri ES, MacDonald K, Speert D, et al. Cutting edge: NF-kappaB activating pattern recognition and cytokine receptors license NLRP3 inflammasome activation by regulating NLRP3 expression. J Immunol 2009, 183(2): 787-791.

11. Cornut M, Bourdonnay E, Henry T. Transcriptional Regulation of Inflammasomes. Int J Mol Sci 2020, 21(21).

12. Baker PJ, De Nardo D, Moghaddas F, Tran LS, Bachem A, Nguyen T, et al. Posttranslational Modification as a Critical Determinant of Cytoplasmic Innate Immune Recognition. Physiol Rev 2017, 97(3): 1165-1209.

14. Liu Y, Yang D, Liu T, Chen J, Yu J, Yi P. N6-methyladenosine-mediated gene regulation and therapeutic implications. Trends Mol Med 2023, 29(6): 454-467.

15. Zaccara S, Ries RJ, Jaffrey SR. Reading, writing and erasing mRNA methylation. Nat Rev Mol Cell Biol 2019, 20(10): 608-624.

16. Zhao Y, Shi Y, Shen H, Xie W. m(6)A-binding proteins: the emerging crucial performers in epigenetics. J Hematol Oncol 2020, 13(1): 35.

17. Xu H, Lin C, Yang J, Chen X, Chen Y, Chen J, et al. The Role of N(6)-Methyladenosine in Inflammatory Diseases. Oxid Med Cell Longev 2022, 2022: 9744771.

18. Peng Z, Gong Y, Wang X, He W, Wu L, Zhang L, et al. METTL3-m(6)A-Rubicon axis inhibits autophagy in nonalcoholic fatty liver disease. Mol Ther 2022, 30(2): 932-946.

19. Zhang J, Song B, Zeng Y, Xu C, Gao L, Guo Y, et al. m6A modification in inflammatory bowel disease provides new insights into clinical applications. Biomed Pharmacother 2023, 159: 114298.

20. Winkler R, Gillis E, Lasman L, Safra M, Geula S, Soyris C, et al. m(6)A modification controls the innate immune response to infection by targeting type I interferons. Nat Immunol 2019, 20(2): 173-182.

21. Zhang H, Liu J, Zhou Y, Qu M, Wang Y, Guo K, et al. Neutrophil extracellular traps mediate m(6)A modification and regulates sepsis-associated acute lung injury by activating ferroptosis in alveolar epithelial cells. Int J Biol Sci 2022, 18(8): 3337-3357.

22. Jiao Y, Zhang T, Zhang C, Ji H, Tong X, Xia R, et al. Exosomal miR-30d-5p of neutrophils induces M1 macrophage polarization and primes macrophage pyroptosis in sepsis-related acute lung injury. Crit Care 2021, 25(1): 356.

23. Huang X, Zhang H, Guo X, Zhu Z, Cai H, Kong X. Insulin-like growth factor 2 mRNA-binding protein 1 (IGF2BP1) in cancer. J Hematol Oncol 2018, 11(1): 88.

24. Huang H, Weng H, Chen J. m(6)A Modification in Coding and Non-coding RNAs: Roles and Therapeutic Implications in Cancer. Cancer Cell 2020, 37(3): 270-288.

25. Zhu X, Tang H, Yang M, Yin K. N6-methyladenosine in macrophage function: a novel target for metabolic diseases. Trends Endocrinol Metab 2023, 34(2): 66-84.

26. Kumari R, Ranjan P, Suleiman ZG, Goswami SK, Li J, Prasad R, et al. mRNA modifications in cardiovascular biology and disease: with a focus on m6A modification. Cardiovasc Res 2022, 118(7): 1680-1692.

28. Weng H, Huang H, Wu H, Qin X, Zhao BS, Dong L, et al. METTL14 Inhibits Hematopoietic Stem/Progenitor Differentiation and Promotes Leukemogenesis via mRNA m(6)A Modification. Cell Stem Cell 2018, 22(2): 191-205 e199.

29. Zhang H, Wu D, Wang Y, Guo K, Spencer CB, Ortoga L, et al. METTL3-mediated N6-methyladenosine exacerbates ferroptosis via m6A-IGF2BP2-dependent mitochondrial metabolic reprogramming in sepsis-induced acute lung injury. Clin Transl Med 2023, 13(9): e1389.

30. Qu M, Chen Z, Qiu Z, Nan K, Wang Y, Shi Y, et al. Neutrophil extracellular traps-triggered impaired autophagic flux via METTL3 underlies sepsis-associated acute lung injury. Cell Death Discov 2022, 8(1): 375.

31. Chen Y, Wu Y, Zhu L, Chen C, Xu S, Tang D, et al. METTL3-Mediated N6-Methyladenosine Modification of Trim59 mRNA Protects Against Sepsis-Induced Acute Respiratory Distress Syndrome. Front Immunol 2022, 13: 897487.

32. Chen X, Tang J, Shuai W, Meng J, Feng J, Han Z. Macrophage polarization and its role in the pathogenesis of acute lung injury/acute respiratory distress syndrome. Inflamm Res 2020, 69(9): 883-895.

33. Zaslona Z, Przybranowski S, Wilke C, van Rooijen N, Teitz-Tennenbaum S, Osterholzer JJ, et al. Resident alveolar macrophages suppress, whereas recruited monocytes promote, allergic lung inflammation in murine models of asthma. J Immunol 2014, 193(8): 4245-4253.

34. Han W, Tanjore H, Liu Y, Hunt RP, Gutor SS, Serezani APM, et al. Identification and Characterization of Alveolar and Recruited Lung Macrophages during Acute Lung Inflammation. J Immunol 2023, 210(11): 1827-1836.

35. Li Y, Li J, Yu Q, Ji L, Peng B. METTL14 regulates microglia/macrophage polarization and NLRP3 inflammasome activation after ischemic stroke by the KAT3B-STING axis. Neurobiol Dis 2023, 185: 106253.

36. Zheng Y, Li Y, Ran X, Wang D, Zheng X, Zhang M, et al. Mettl14 mediates the inflammatory response of macrophages in atherosclerosis through the NF-kappaB/IL-6 signaling pathway. Cell Mol Life Sci 2022, 79(6): 311.

37. Dang W, Tao Y, Xu X, Zhao H, Zou L, Li Y. The role of lung macrophages in acute respiratory distress syndrome. Inflamm Res 2022, 71(12): 1417-1432.

38. Wang X, Ding Y, Li R, Zhang R, Ge X, Gao R, et al. N(6)-methyladenosine of Spi2a attenuates inflammation and sepsis-associated myocardial dysfunction in mice. Nat Commun 2023, 14(1): 1185.

39. Mangan MSJ, Olhava EJ, Roush WR, Seidel HM, Glick GD, Latz E. Targeting the NLRP3 inflammasome in inflammatory diseases. Nat Rev Drug Discov 2018, 17(8): 588-606.

40. Wann SR, Chi PL, Huang WC, Cheng CC, Chang YT. Combination therapy of iPSC-derived conditioned medium with ceftriaxone alleviates bacteria-induced lung injury by targeting the NLRP3 inflammasome. J Cell Physiol 2022, 237(2): 1299-1314.

41. Xu Q, Ye Y, Wang Z, Zhu H, Li Y, Wang J, et al. NLRP3 Knockout Protects against Lung Injury Induced by Cerebral Ischemia-Reperfusion. Oxid Med Cell Longev 2022, 2022: 6260102.

42. Bakhshi S, Shamsi S. MCC950 in the treatment of NLRP3-mediated inflammatory diseases: Latest evidence and therapeutic outcomes. Int Immunopharmacol 2022, 106: 108595.

43. Li H, Guan Y, Liang B, Ding P, Hou X, Wei W, et al. Therapeutic potential of MCC950, a specific inhibitor of NLRP3 inflammasome. Eur J Pharmacol 2022, 928: 175091.

44. Meng L, Lin H, Huang X, Weng J, Peng F, Wu S. METTL14 suppresses pyroptosis and diabetic cardiomyopathy by downregulating TINCR lncRNA. Cell Death Dis 2022, 13(1): 38.

45. Yuan X, Li T, Shi L, Miao J, Guo Y, Chen Y. Human umbilical cord mesenchymal stem cells deliver exogenous miR-26a-5p via exosomes to inhibit nucleus pulposus cell pyroptosis through METTL14/NLRP3. Mol Med 2021, 27(1): 91.

1. Meyer NJ, Gattinoni L, Calfee CS. Acute respiratory distress syndrome. Lancet 2021, 398(10300): 622-637.

2. Gorman EA, O'Kane CM, McAuley DF. Acute respiratory distress syndrome in adults: diagnosis, outcomes, long-term sequelae, and management. Lancet 2022, 400(10358): 1157-1170.

3. Bos LDJ, Ware LB. Acute respiratory distress syndrome: causes, pathophysiology, and phenotypes. Lancet 2022, 400(10358): 1145-1156.

4. Gilroy DW, De Maeyer RPH, Tepper M, O'Brien A, Uddin M, Chen J, et al. Treating exuberant, non-resolving inflammation in the lung; Implications for acute respiratory distress syndrome and COVID-19. Pharmacol Ther 2021, 221: 107745.

5. Laffey JG, Matthay MA. Fifty Years of Research in ARDS. Cell-based Therapy for Acute Respiratory Distress Syndrome. Biology and Potential Therapeutic Value. Am J Respir Crit Care Med 2017, 196(3): 266-273.

6. Xian H, Liu Y, Rundberg Nilsson A, Gatchalian R, Crother TR, Tourtellotte WG, et al. Metformin inhibition of mitochondrial ATP and DNA synthesis abrogates NLRP3 inflammasome activation and pulmonary inflammation. Immunity 2021, 54(7): 1463-1477 e1411.

7. Paik S, Kim JK, Silwal P, Sasakawa C, Jo EK. An update on the regulatory mechanisms of NLRP3 inflammasome activation. Cell Mol Immunol 2021, 18(5): 1141-1160.

8. Kelley N, Jeltema D, Duan Y, He Y. The NLRP3 Inflammasome: An Overview of Mechanisms of Activation and Regulation. Int J Mol Sci 2019, 20(13).

9. Swanson KV, Deng M, Ting JP. The NLRP3 inflammasome: molecular activation and regulation to therapeutics. Nat Rev Immunol 2019, 19(8): 477-489.

10. Bauernfeind FG, Horvath G, Stutz A, Alnemri ES, MacDonald K, Speert D, et al. Cutting edge: NF-kappaB activating pattern recognition and cytokine receptors license NLRP3 inflammasome activation by regulating NLRP3 expression. J Immunol 2009, 183(2): 787-791.

11. Cornut M, Bourdonnay E, Henry T. Transcriptional Regulation of Inflammasomes. Int J Mol Sci 2020, 21(21).

12. Baker PJ, De Nardo D, Moghaddas F, Tran LS, Bachem A, Nguyen T, et al. Posttranslational Modification as a Critical Determinant of Cytoplasmic Innate Immune Recognition. Physiol Rev 2017, 97(3): 1165-1209.

13. Moretti J, Blander JM. Increasing complexity of NLRP3 inflammasome regulation. J Leukoc Biol 2021, 109(3): 561-571.

14. Liu Y, Yang D, Liu T, Chen J, Yu J, Yi P. N6-methyladenosine-mediated gene regulation and therapeutic implications. Trends Mol Med 2023, 29(6): 454-467.

15. Zaccara S, Ries RJ, Jaffrey SR. Reading, writing and erasing mRNA methylation. Nat Rev Mol Cell Biol 2019, 20(10): 608-624.

16. Zhao Y, Shi Y, Shen H, Xie W. m(6)A-binding proteins: the emerging crucial performers in epigenetics. J Hematol Oncol 2020, 13(1): 35.

17. Xu H, Lin C, Yang J, Chen X, Chen Y, Chen J, et al. The Role of N(6)-Methyladenosine in Inflammatory Diseases. Oxid Med Cell Longev 2022, 2022: 9744771.

18. Peng Z, Gong Y, Wang X, He W, Wu L, Zhang L, et al. METTL3-m(6)A-Rubicon axis inhibits autophagy in nonalcoholic fatty liver disease. Mol Ther 2022, 30(2): 932-946.

19. Zhang J, Song B, Zeng Y, Xu C, Gao L, Guo Y, et al. m6A modification in inflammatory bowel disease provides new insights into clinical applications. Biomed Pharmacother 2023, 159: 114298.

20. Winkler R, Gillis E, Lasman L, Safra M, Geula S, Soyris C, et al. m(6)A modification controls the innate immune response to infection by targeting type I interferons. Nat Immunol 2019, 20(2): 173-182.

21. Zhang H, Liu J, Zhou Y, Qu M, Wang Y, Guo K, et al. Neutrophil extracellular traps mediate m(6)A modification and regulates sepsis-associated acute lung injury by activating ferroptosis in alveolar epithelial cells. Int J Biol Sci 2022, 18(8): 3337-3357.

23. Huang X, Zhang H, Guo X, Zhu Z, Cai H, Kong X. Insulin-like growth factor 2 mRNA-binding protein 1 (IGF2BP1) in cancer. J Hematol Oncol 2018, 11(1): 88.

24. Huang H, Weng H, Chen J. m(6)A Modification in Coding and Non-coding RNAs: Roles and Therapeutic Implications in Cancer. Cancer Cell 2020, 37(3): 270-288.

25. Zhu X, Tang H, Yang M, Yin K. N6-methyladenosine in macrophage function: a novel target for metabolic diseases. Trends Endocrinol Metab 2023, 34(2): 66-84.

26. Kumari R, Ranjan P, Suleiman ZG, Goswami SK, Li J, Prasad R, et al. mRNA modifications in cardiovascular biology and disease: with a focus on m6A modification. Cardiovasc Res 2022, 118(7): 1680-1692.

27. Luo J, Xu T, Sun K. N6-Methyladenosine RNA Modification in Inflammation: Roles, Mechanisms, and Applications. Front Cell Dev Biol 2021, 9: 670711.

28. Weng H, Huang H, Wu H, Qin X, Zhao BS, Dong L, et al. METTL14 Inhibits Hematopoietic Stem/Progenitor Differentiation and Promotes Leukemogenesis via mRNA m(6)A Modification. Cell Stem Cell 2018, 22(2): 191-205 e199.

29. Zhang H, Wu D, Wang Y, Guo K, Spencer CB, Ortoga L, et al. METTL3-mediated N6-methyladenosine exacerbates ferroptosis via m6A-IGF2BP2-dependent mitochondrial metabolic reprogramming in sepsis-induced acute lung injury. Clin Transl Med 2023, 13(9): e1389.

30. Qu M, Chen Z, Qiu Z, Nan K, Wang Y, Shi Y, et al. Neutrophil extracellular traps-triggered impaired autophagic flux via METTL3 underlies sepsis-associated acute lung injury. Cell Death Discov 2022, 8(1): 375.

31. Chen Y, Wu Y, Zhu L, Chen C, Xu S, Tang D, et al. METTL3-Mediated N6-Methyladenosine Modification of Trim59 mRNA Protects Against Sepsis-Induced Acute Respiratory Distress Syndrome. Front Immunol 2022, 13: 897487.

32. Chen X, Tang J, Shuai W, Meng J, Feng J, Han Z. Macrophage polarization and its role in the pathogenesis of acute lung injury/acute respiratory distress syndrome. Inflamm Res 2020, 69(9): 883-895.

33. Zaslona Z, Przybranowski S, Wilke C, van Rooijen N, Teitz-Tennenbaum S, Osterholzer JJ, et al. Resident alveolar macrophages suppress, whereas recruited monocytes promote, allergic lung inflammation in murine models of asthma. J Immunol 2014, 193(8): 4245-4253.

34. Han W, Tanjore H, Liu Y, Hunt RP, Gutor SS, Serezani APM, et al. Identification and Characterization of Alveolar and Recruited Lung Macrophages during Acute Lung Inflammation. J Immunol 2023, 210(11): 1827-1836.

35. Li Y, Li J, Yu Q, Ji L, Peng B. METTL14 regulates microglia/macrophage polarization and NLRP3 inflammasome activation after ischemic stroke by the KAT3B-STING axis. Neurobiol Dis 2023, 185: 106253.

36. Zheng Y, Li Y, Ran X, Wang D, Zheng X, Zhang M, et al. Mettl14 mediates the inflammatory response of macrophages in atherosclerosis through the NF-kappaB/IL-6 signaling pathway. Cell Mol Life Sci 2022, 79(6): 311.

37. Dang W, Tao Y, Xu X, Zhao H, Zou L, Li Y. The role of lung macrophages in acute respiratory distress syndrome. Inflamm Res 2022, 71(12): 1417-1432.

38. Wang X, Ding Y, Li R, Zhang R, Ge X, Gao R, et al. N(6)-methyladenosine of Spi2a attenuates inflammation and sepsis-associated myocardial dysfunction in mice. Nat Commun 2023, 14(1): 1185.

39. Mangan MSJ, Olhava EJ, Roush WR, Seidel HM, Glick GD, Latz E. Targeting the NLRP3 inflammasome in inflammatory diseases. Nat Rev Drug Discov 2018, 17(8): 588-606.

40. Wann SR, Chi PL, Huang WC, Cheng CC, Chang YT. Combination therapy of iPSC-derived conditioned medium with ceftriaxone alleviates bacteria-induced lung injury by targeting the NLRP3 inflammasome. J Cell Physiol 2022, 237(2): 1299-1314.

41. Xu Q, Ye Y, Wang Z, Zhu H, Li Y, Wang J, et al. NLRP3 Knockout Protects against Lung Injury Induced by Cerebral Ischemia-Reperfusion. Oxid Med Cell Longev 2022, 2022: 6260102.

42. Bakhshi S, Shamsi S. MCC950 in the treatment of NLRP3-mediated inflammatory diseases: Latest evidence and therapeutic outcomes. Int Immunopharmacol 2022, 106: 108595.

43. Li H, Guan Y, Liang B, Ding P, Hou X, Wei W, et al. Therapeutic potential of MCC950, a specific inhibitor of NLRP3 inflammasome. Eur J Pharmacol 2022, 928: 175091.

44. Meng L, Lin H, Huang X, Weng J, Peng F, Wu S. METTL14 suppresses pyroptosis and diabetic cardiomyopathy by downregulating TINCR lncRNA. Cell Death Dis 2022, 13(1): 38.

45. Yuan X, Li T, Shi L, Miao J, Guo Y, Chen Y. Human umbilical cord mesenchymal stem cells deliver exogenous miR-26a-5p via exosomes to inhibit nucleus pulposus cell pyroptosis through METTL14/NLRP3. Mol Med 2021, 27(1): 91.

46. Huang H, Weng H, Sun W, Qin X, Shi H, Wu H, et al. Recognition of RNA N(6)-methyladenosine by IGF2BP proteins enhances mRNA stability and translation. Nat Cell Biol 2018, 20(3): 285-295.

1. Meyer NJ, Gattinoni L, Calfee CS. Acute respiratory distress syndrome. Lancet 2021, 398(10300): 622-637.

2. Gorman EA, O'Kane CM, McAuley DF. Acute respiratory distress syndrome in adults: diagnosis, outcomes, long-term sequelae, and management. Lancet 2022, 400(10358): 1157-1170.

3. Bos LDJ, Ware LB. Acute respiratory distress syndrome: causes, pathophysiology, and phenotypes. Lancet 2022, 400(10358): 1145-1156.

4. Gilroy DW, De Maeyer RPH, Tepper M, O'Brien A, Uddin M, Chen J, et al. Treating exuberant, non-resolving inflammation in the lung; Implications for acute respiratory distress syndrome and COVID-19. Pharmacol Ther 2021, 221: 107745.

5. Laffey JG, Matthay MA. Fifty Years of Research in ARDS. Cell-based Therapy for Acute Respiratory Distress Syndrome. Biology and Potential Therapeutic Value. Am J Respir Crit Care Med 2017, 196(3): 266-273.

6. Xian H, Liu Y, Rundberg Nilsson A, Gatchalian R, Crother TR, Tourtellotte WG, et al. Metformin inhibition of mitochondrial ATP and DNA synthesis abrogates NLRP3 inflammasome activation and pulmonary inflammation. Immunity 2021, 54(7): 1463-1477 e1411.

7. Paik S, Kim JK, Silwal P, Sasakawa C, Jo EK. An update on the regulatory mechanisms of NLRP3 inflammasome activation. Cell Mol Immunol 2021, 18(5): 1141-1160.

8. Kelley N, Jeltema D, Duan Y, He Y. The NLRP3 Inflammasome: An Overview of Mechanisms of Activation and Regulation. Int J Mol Sci 2019, 20(13).

9. Swanson KV, Deng M, Ting JP. The NLRP3 inflammasome: molecular activation and regulation to therapeutics. Nat Rev Immunol 2019, 19(8): 477-489.

10. Bauernfeind FG, Horvath G, Stutz A, Alnemri ES, MacDonald K, Speert D, et al. Cutting edge: NF-kappaB activating pattern recognition and cytokine receptors license NLRP3 inflammasome activation by regulating NLRP3 expression. J Immunol 2009, 183(2): 787-791.

11. Cornut M, Bourdonnay E, Henry T. Transcriptional Regulation of Inflammasomes. Int J Mol Sci 2020, 21(21).

12. Baker PJ, De Nardo D, Moghaddas F, Tran LS, Bachem A, Nguyen T, et al. Posttranslational Modification as a Critical Determinant of Cytoplasmic Innate Immune Recognition. Physiol Rev 2017, 97(3): 1165-1209.

13. Moretti J, Blander JM. Increasing complexity of NLRP3 inflammasome regulation. J Leukoc Biol 2021, 109(3): 561-571.

14. Liu Y, Yang D, Liu T, Chen J, Yu J, Yi P. N6-methyladenosine-mediated gene regulation and therapeutic implications. Trends Mol Med 2023, 29(6): 454-467.

15. Zaccara S, Ries RJ, Jaffrey SR. Reading, writing and erasing mRNA methylation. Nat Rev Mol Cell Biol 2019, 20(10): 608-624.

16. Zhao Y, Shi Y, Shen H, Xie W. m(6)A-binding proteins: the emerging crucial performers in epigenetics. J Hematol Oncol 2020, 13(1): 35.

17. Xu H, Lin C, Yang J, Chen X, Chen Y, Chen J, et al. The Role of N(6)-Methyladenosine in Inflammatory Diseases. Oxid Med Cell Longev 2022, 2022: 9744771.

18. Peng Z, Gong Y, Wang X, He W, Wu L, Zhang L, et al. METTL3-m(6)A-Rubicon axis inhibits autophagy in nonalcoholic fatty liver disease. Mol Ther 2022, 30(2): 932-946.

19. Zhang J, Song B, Zeng Y, Xu C, Gao L, Guo Y, et al. m6A modification in inflammatory bowel disease provides new insights into clinical applications. Biomed Pharmacother 2023, 159: 114298.

20. Winkler R, Gillis E, Lasman L, Safra M, Geula S, Soyris C, et al. m(6)A modification controls the innate immune response to infection by targeting type I interferons. Nat Immunol 2019, 20(2): 173-182.

21. Zhang H, Liu J, Zhou Y, Qu M, Wang Y, Guo K, et al. Neutrophil extracellular traps mediate m(6)A modification and regulates sepsis-associated acute lung injury by activating ferroptosis in alveolar epithelial cells. Int J Biol Sci 2022, 18(8): 3337-3357.

22. Jiao Y, Zhang T, Zhang C, Ji H, Tong X, Xia R, et al. Exosomal miR-30d-5p of neutrophils induces M1 macrophage polarization and primes macrophage pyroptosis in sepsis-related acute lung injury. Crit Care 2021, 25(1): 356.

23. Huang X, Zhang H, Guo X, Zhu Z, Cai H, Kong X. Insulin-like growth factor 2 mRNA-binding protein 1 (IGF2BP1) in cancer. J Hematol Oncol 2018, 11(1): 88.

24. Huang H, Weng H, Chen J. m(6)A Modification in Coding and Non-coding RNAs: Roles and Therapeutic Implications in Cancer. Cancer Cell 2020, 37(3): 270-288.

25. Zhu X, Tang H, Yang M, Yin K. N6-methyladenosine in macrophage function: a novel target for metabolic diseases. Trends Endocrinol Metab 2023, 34(2): 66-84.

26. Kumari R, Ranjan P, Suleiman ZG, Goswami SK, Li J, Prasad R, et al. mRNA modifications in cardiovascular biology and disease: with a focus on m6A modification. Cardiovasc Res 2022, 118(7): 1680-1692.

27. Luo J, Xu T, Sun K. N6-Methyladenosine RNA Modification in Inflammation: Roles, Mechanisms, and Applications. Front Cell Dev Biol 2021, 9: 670711.

28. Weng H, Huang H, Wu H, Qin X, Zhao BS, Dong L, et al. METTL14 Inhibits Hematopoietic Stem/Progenitor Differentiation and Promotes Leukemogenesis via mRNA m(6)A Modification. Cell Stem Cell 2018, 22(2): 191-205 e199.

29. Zhang H, Wu D, Wang Y, Guo K, Spencer CB, Ortoga L, et al. METTL3-mediated N6-methyladenosine exacerbates ferroptosis via m6A-IGF2BP2-dependent mitochondrial metabolic reprogramming in sepsis-induced acute lung injury. Clin Transl Med 2023, 13(9): e1389.

30. Qu M, Chen Z, Qiu Z, Nan K, Wang Y, Shi Y, et al. Neutrophil extracellular traps-triggered impaired autophagic flux via METTL3 underlies sepsis-associated acute lung injury. Cell Death Discov 2022, 8(1): 375.

31. Chen Y, Wu Y, Zhu L, Chen C, Xu S, Tang D, et al. METTL3-Mediated N6-Methyladenosine Modification of Trim59 mRNA Protects Against Sepsis-Induced Acute Respiratory Distress Syndrome. Front Immunol 2022, 13: 897487.

32. Chen X, Tang J, Shuai W, Meng J, Feng J, Han Z. Macrophage polarization and its role in the pathogenesis of acute lung injury/acute respiratory distress syndrome. Inflamm Res 2020, 69(9): 883-895.

34. Han W, Tanjore H, Liu Y, Hunt RP, Gutor SS, Serezani APM, et al. Identification and Characterization of Alveolar and Recruited Lung Macrophages during Acute Lung Inflammation. J Immunol 2023, 210(11): 1827-1836.

35. Li Y, Li J, Yu Q, Ji L, Peng B. METTL14 regulates microglia/macrophage polarization and NLRP3 inflammasome activation after ischemic stroke by the KAT3B-STING axis. Neurobiol Dis 2023, 185: 106253.

36. Zheng Y, Li Y, Ran X, Wang D, Zheng X, Zhang M, et al. Mettl14 mediates the inflammatory response of macrophages in atherosclerosis through the NF-kappaB/IL-6 signaling pathway. Cell Mol Life Sci 2022, 79(6): 311.

37. Dang W, Tao Y, Xu X, Zhao H, Zou L, Li Y. The role of lung macrophages in acute respiratory distress syndrome. Inflamm Res 2022, 71(12): 1417-1432.

38. Wang X, Ding Y, Li R, Zhang R, Ge X, Gao R, et al. N(6)-methyladenosine of Spi2a attenuates inflammation and sepsis-associated myocardial dysfunction in mice. Nat Commun 2023, 14(1): 1185.

39. Mangan MSJ, Olhava EJ, Roush WR, Seidel HM, Glick GD, Latz E. Targeting the NLRP3 inflammasome in inflammatory diseases. Nat Rev Drug Discov 2018, 17(8): 588-606.

41. Xu Q, Ye Y, Wang Z, Zhu H, Li Y, Wang J, et al. NLRP3 Knockout Protects against Lung Injury Induced by Cerebral Ischemia-Reperfusion. Oxid Med Cell Longev 2022, 2022: 6260102.

42. Bakhshi S, Shamsi S. MCC950 in the treatment of NLRP3-mediated inflammatory diseases: Latest evidence and therapeutic outcomes. Int Immunopharmacol 2022, 106: 108595.

43. Li H, Guan Y, Liang B, Ding P, Hou X, Wei W, et al. Therapeutic potential of MCC950, a specific inhibitor of NLRP3 inflammasome. Eur J Pharmacol 2022, 928: 175091.

44. Meng L, Lin H, Huang X, Weng J, Peng F, Wu S. METTL14 suppresses pyroptosis and diabetic cardiomyopathy by downregulating TINCR lncRNA. Cell Death Dis 2022, 13(1): 38.

45. Yuan X, Li T, Shi L, Miao J, Guo Y, Chen Y. Human umbilical cord mesenchymal stem cells deliver exogenous miR-26a-5p via exosomes to inhibit nucleus pulposus cell pyroptosis through METTL14/NLRP3. Mol Med 2021, 27(1): 91.

46. Huang H, Weng H, Sun W, Qin X, Shi H, Wu H, et al. Recognition of RNA N(6)-methyladenosine by IGF2BP proteins enhances mRNA stability and translation. Nat Cell Biol 2018, 20(3): 285-295.

47. Cao F, Tian X, Li Z, Lv Y, Han J, Zhuang R, et al. Suppression of NLRP3 Inflammasome by Erythropoietin via the EPOR/JAK2/STAT3 Pathway Contributes to Attenuation of Acute Lung Injury in Mice. Front Pharmacol 2020, 11: 306.
