# Supplementary material for: METTL14 contributes to acute lung injury by stabilizing NLRP3 expression in an IGF2BP2-dependent manner
Source: Cell Death Dis. 2024 Jan 13;15(1):43. doi: 10.1038/s41419-023-06407-6 (PMC10787837; doi:10.1038/s41419-023-06407-6)
Supplement: Supplementary file 7 — Original western blots [file 41419_2023_6407_MOESM7_ESM.pptx]

## Slide 1
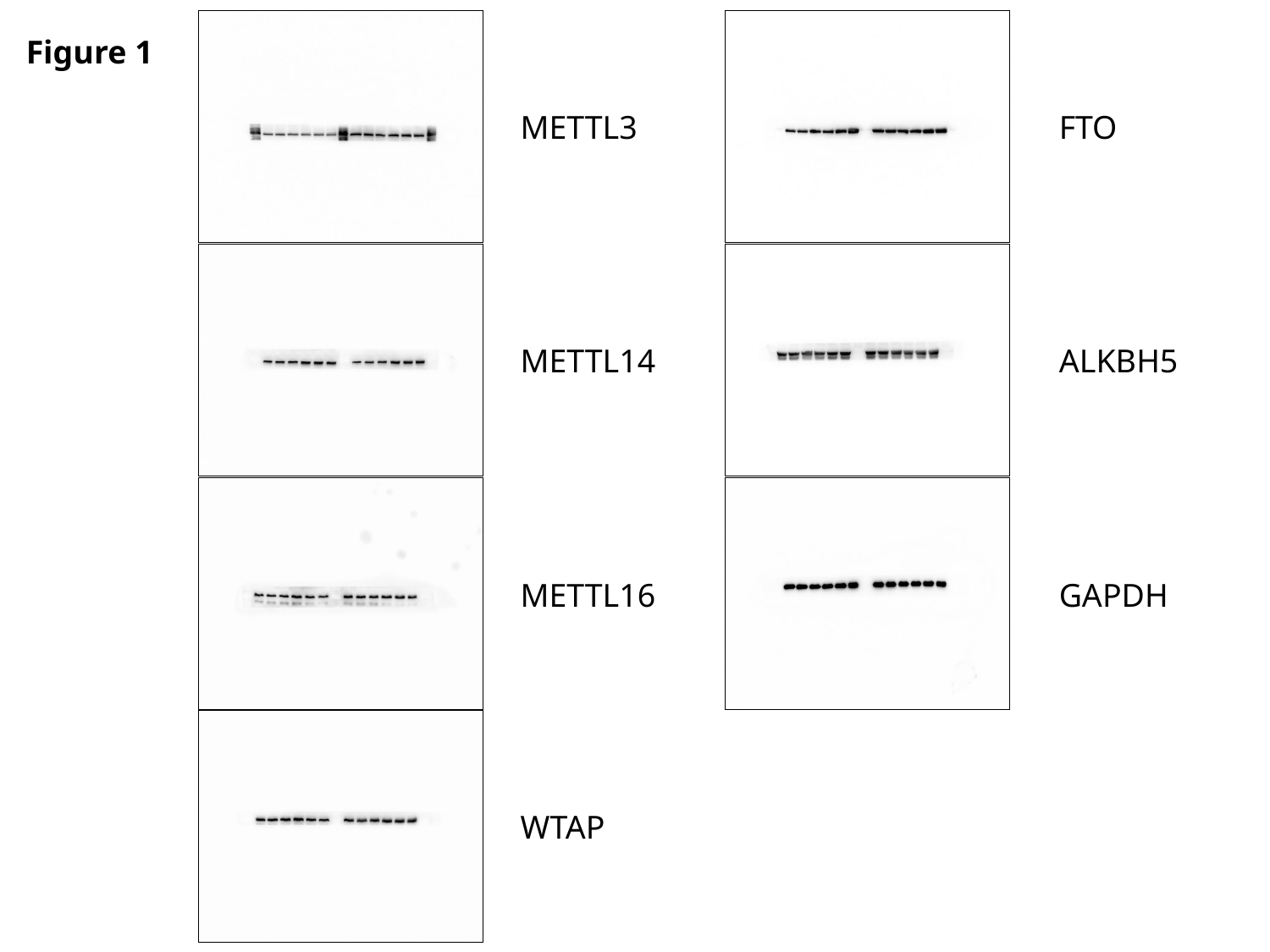

Figure 1
METTL3
FTO
METTL14
ALKBH5
METTL16
GAPDH
WTAP

## Slide 2
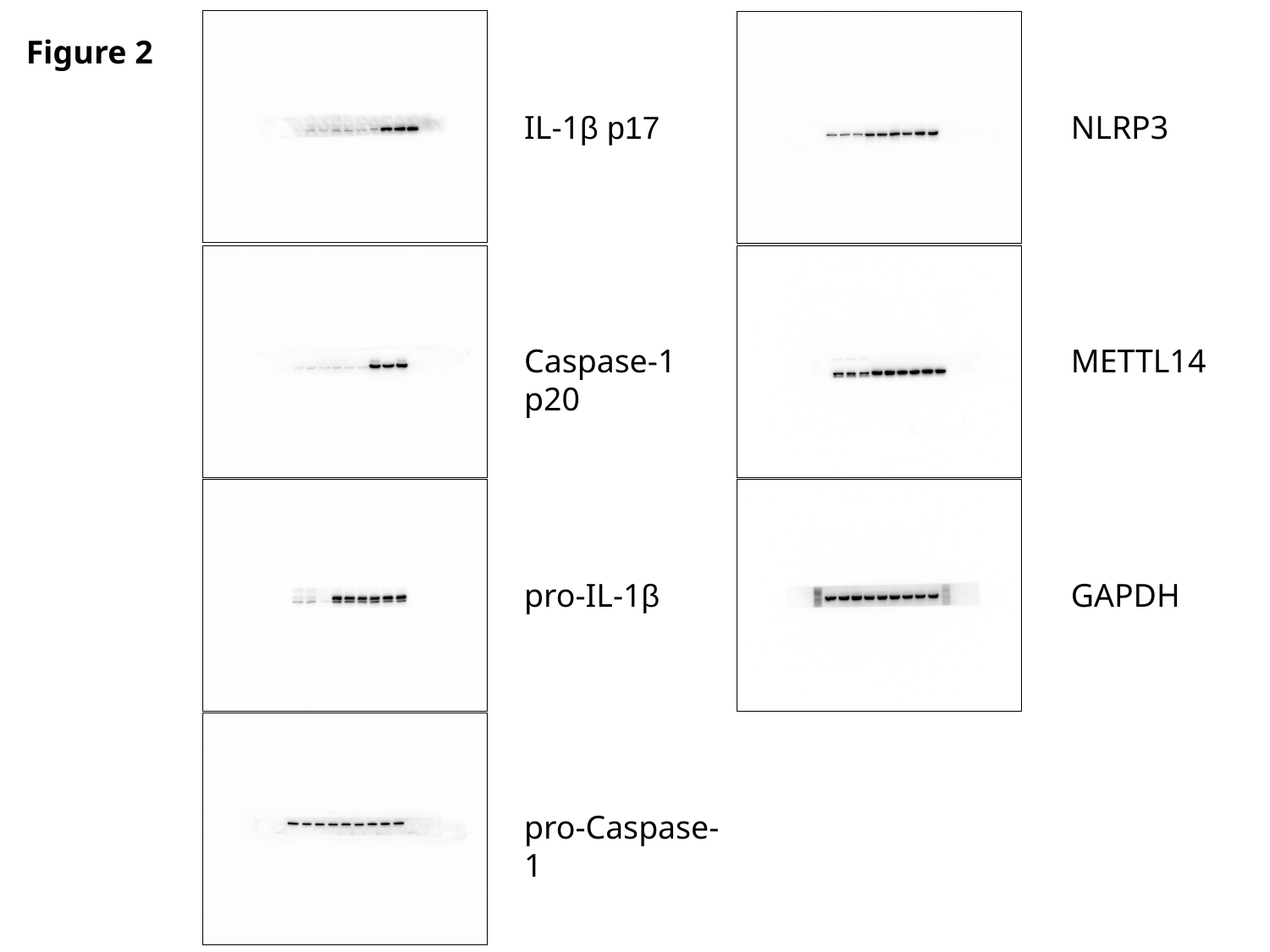

Figure 2
IL-1β p17
NLRP3
Caspase-1 p20
METTL14
pro-IL-1β
GAPDH
pro-Caspase-1

## Slide 3
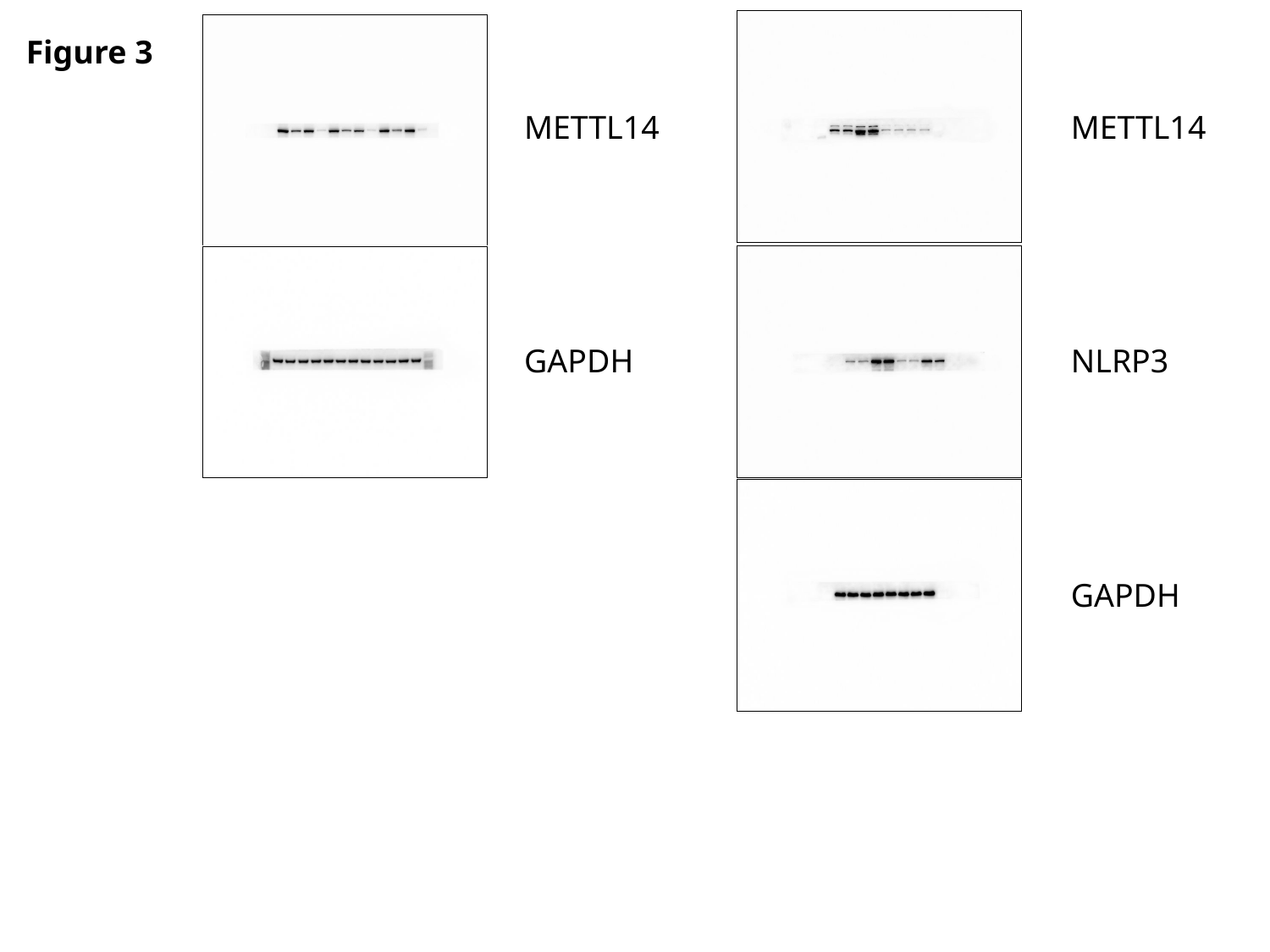

Figure 3
METTL14
METTL14
GAPDH
NLRP3
GAPDH

## Slide 4
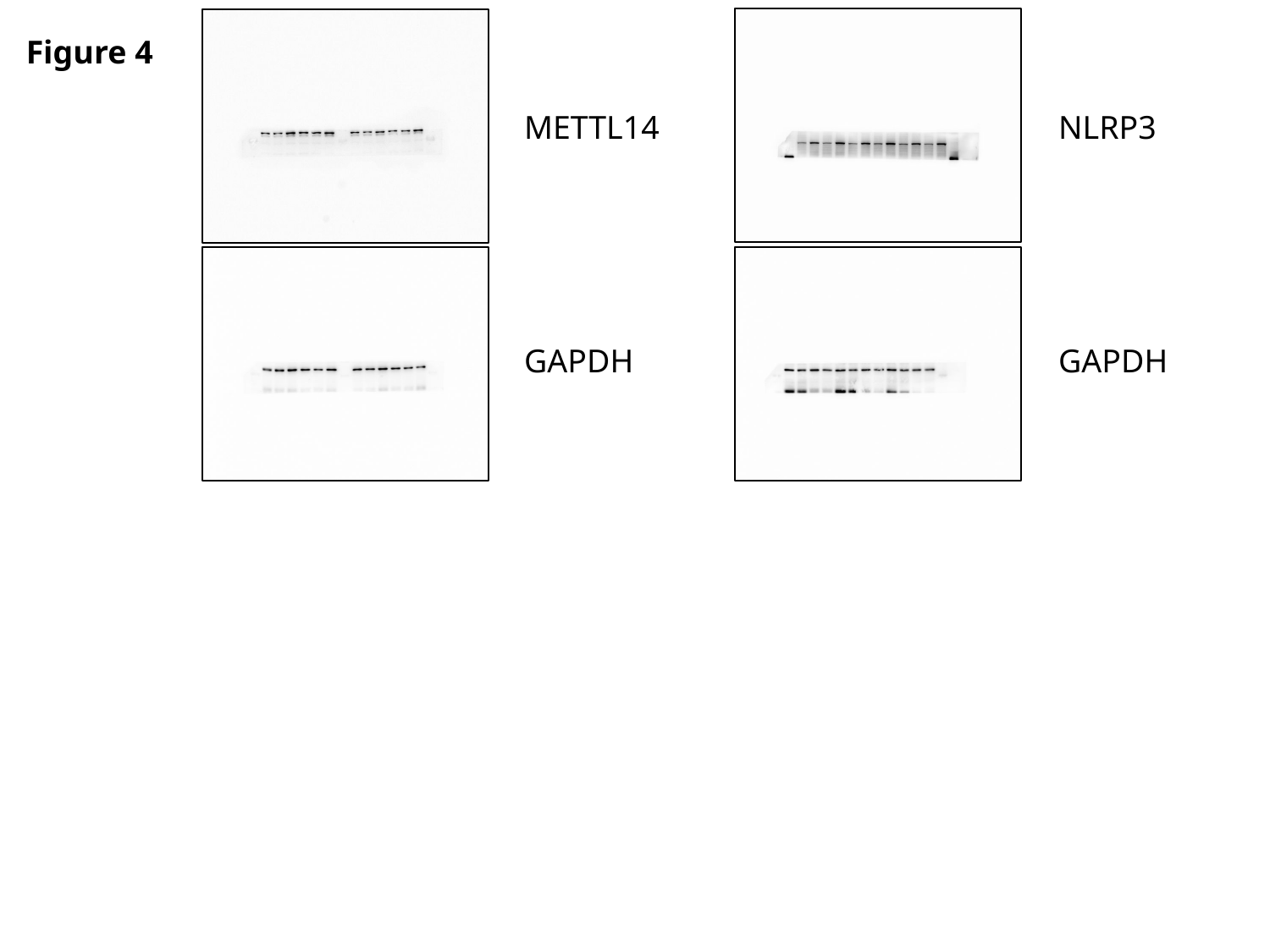

Figure 4
METTL14
NLRP3
GAPDH
GAPDH

## Slide 5
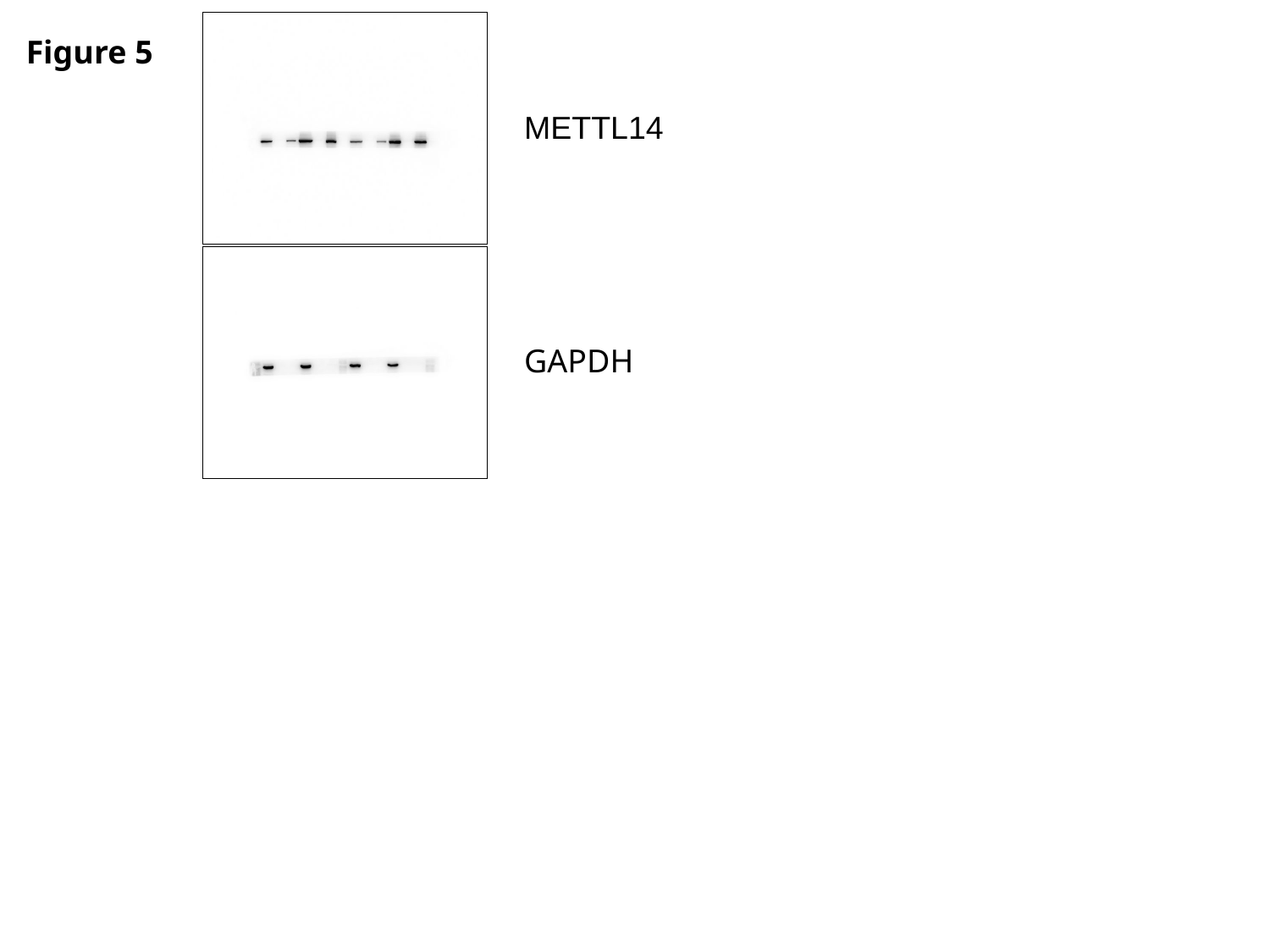

Figure 5
METTL14
GAPDH

## Slide 6
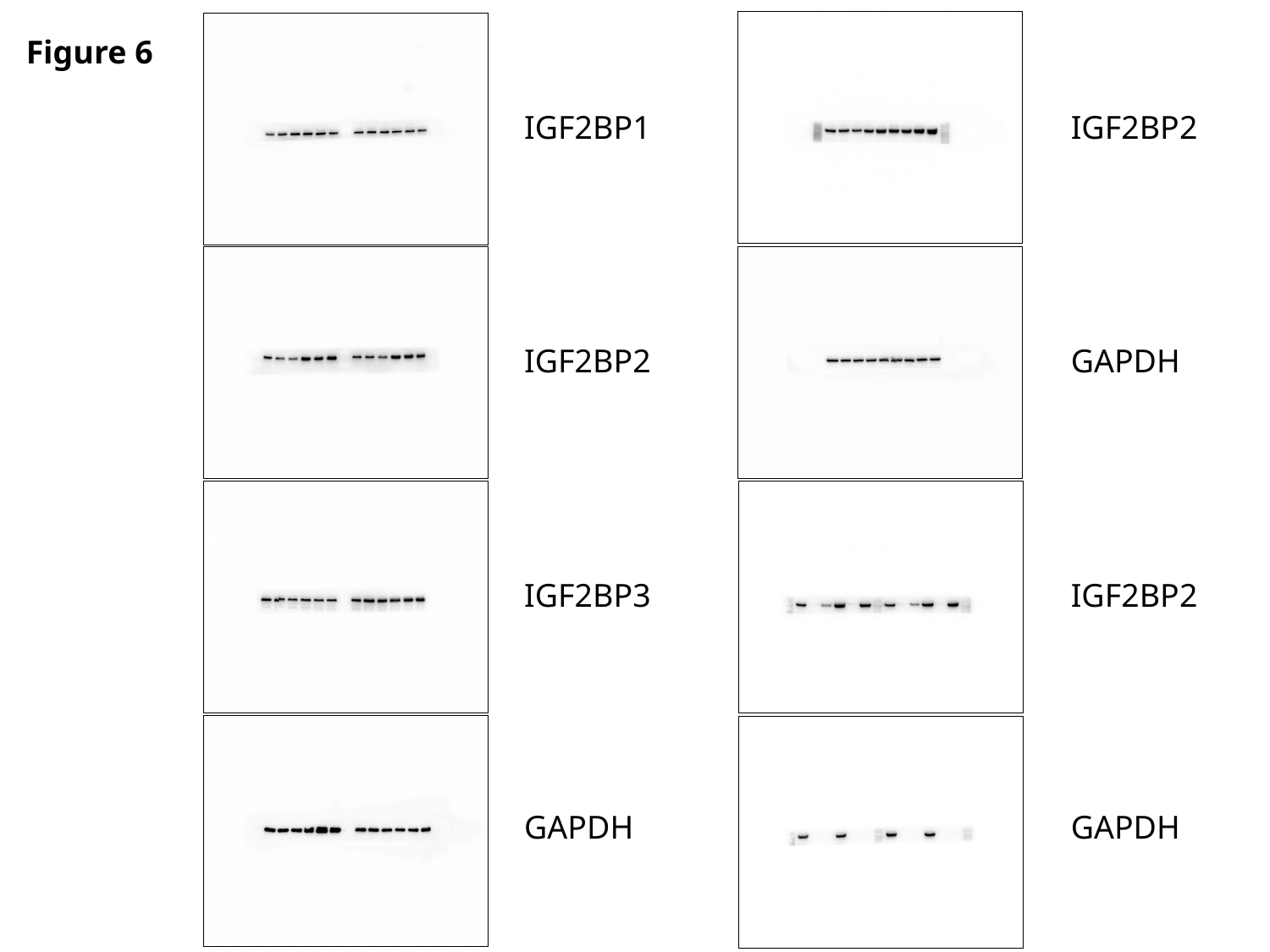

Figure 6
IGF2BP1
IGF2BP2
IGF2BP2
GAPDH
IGF2BP3
IGF2BP2
GAPDH
GAPDH

## Slide 7
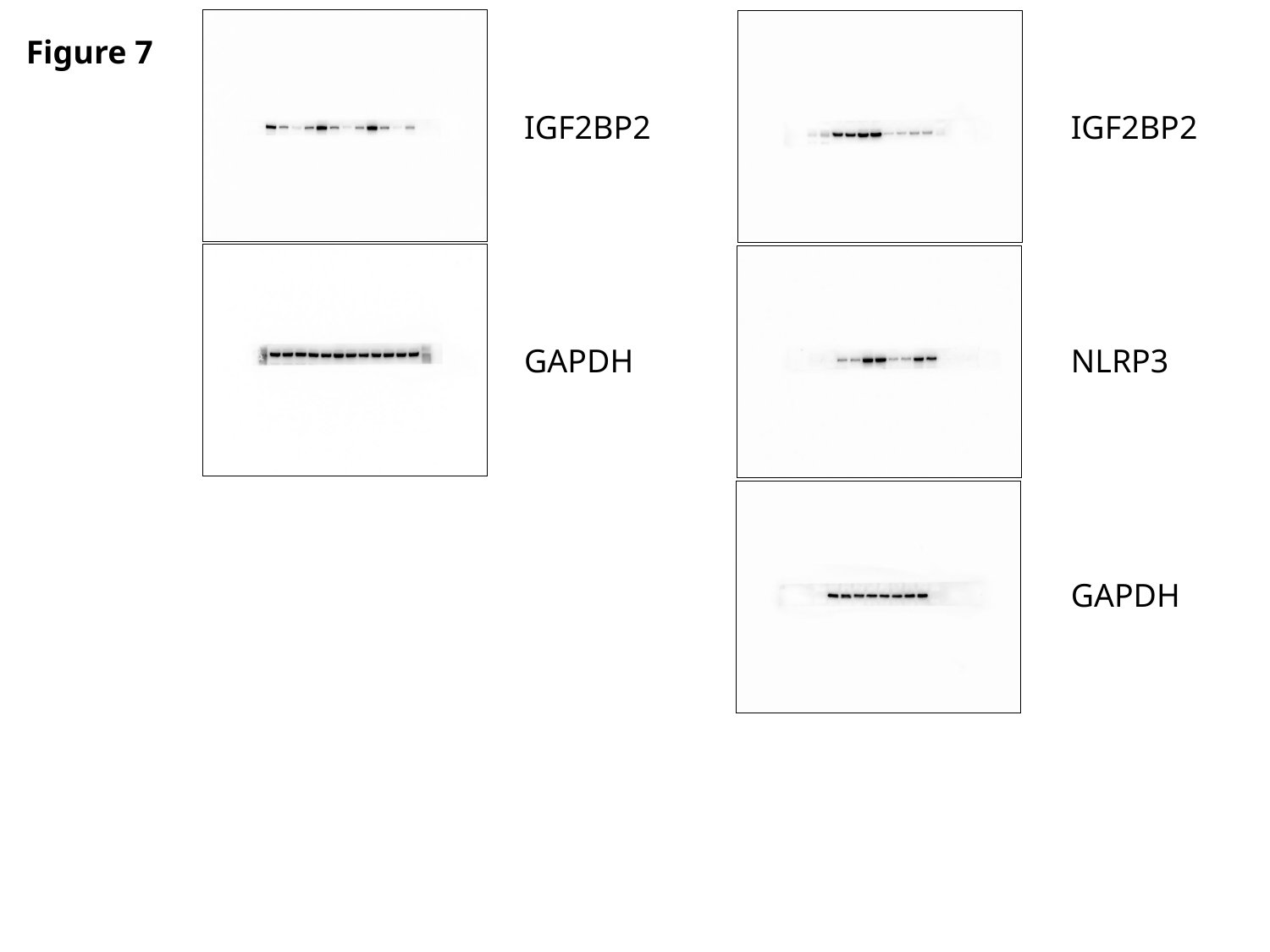

Figure 7
IGF2BP2
IGF2BP2
GAPDH
NLRP3
GAPDH
